# Supplementary material for: The Gene Structure and Expression Level Changes of the GH3 Gene Family in Brassica napus Relative to Its Diploid Ancestors
Source: Genes (Basel). 2019 Jan 17;10(1):58. doi: 10.3390/genes10010058 (PMC6356818; doi:10.3390/genes10010058)
Supplement: Supplementary file 1 [file genes-10-00058-s001.zip › Supplementary files/Supplementary Figures/Figure S1.pdf]

Figure S1 The sequence alignment of 148 proteins

```
AtGH3-1            ............................................................
AtGH3-2            ............................................................
AtGH3-3            ............................................................
AtGH3-4            ............................................................
AtGH3-5            ............................................................
AtGH3-6            ............................................................
AtGH3-7            ............................................................
AtGH3-8            ............................................................
AtGH3-9            ............................................................
AtGH3-10           ............................................................
AtGH3-11           ............................................................
AtGH3-12           ............................................................
AtGH3-13           ............................................................
AtGH3-14           ......................................MFLSPRSAHQKVGLSMLIDPLP
AtGH3-15           ............................................................
AtGH3-16           ............................................................
AtGH3-17           ............................................................
AtGH3-18           ............................................................
AtGH3-19           ............................................................
BraX.GH3-1.a       ............................................................
BraX.GH3-2.a       ............................................................
BraA09.GH3-3.a     ............................................................
BraA01.GH3-5.a     ............................................................
BraA03.GH3-5.b     ............................................................
BraA02.GH3-6.a     ............................................................
BraA03.GH3-6.b     ............................................................
BraA06.GH3-7.a     ............................................................
BraA08.GH3-7.b     ............................................................
BraA02.GH3-8.a     ............................................................
BraA03.GH3-8.b     ............................................................
BraA03.GH3-8.c     ............................................................
BraA03.GH3-8.d     ............................................................
BraA06.GH3-8.e     ............................................................
BraA06.GH3-8.f     ............................................................
BraA09.GH3-8.g     ............................................................
BraA09.GH3-8.h     ............................................................
BraA10.GH3-8.i     ............................................................
BraA09.GH3-10.a    ............................................................
BraA03.GH3-11.a    ............................................................
BraA04.GH3-11.b    ............................................................
BraA05.GH3-11.c    ............................................................
BraA02.GH3-12.a    ............................................................
BraA03.GH3-12.b    ............................................................
BraA06.GH3-12.c    ............................................................
BraA10.GH3-12.d    ............................................................
BraA10.GH3-14.a    ............................................................
BraA03.GH3-15.a    ............................................................
BraA07.GH3-15.b    ............................................................
BraA02.GH3-16.a    ............................................................
BraA07.GH3-17.a    ............................................................
BraA09.GH3-17.b    ............................................................
BraAX.GH3-17.c     ............................................................
BraA04.GH3-19.a    ............................................................
BraA06.GH3-19.b    ............................................................
BraA09.GH3-19.c    ............................................................
BraA09.GH3-19.d    ............................................................
BraAX.GH3-19.e     ............................................................
BolC09.GH3-1.a     ............................................................
BolC01.GH3-2.a     ............................................................
BolC08.GH3-3.a     ............................................................
BolC01.GH3-5.a     ............................................................
BolC07.GH3-5.b     ............................................................
BolC02.GH3-6.a     ............................................................
BolC03.GH3-6.b     ............................................................
BolC06.GH3-7.a     ............................................................
BolC01.GH3-8.a     ............................................................
BolC03.GH3-8.b     ............................................................
BolC07.GH3-8.c     ............................................................
BolC08.GH3-8.d     ............................................................
BolC09.GH3-8.e     ............................................................
BolC09.GH3-10.a    ............................................................
BolC03.GH3-11.a    ............................................................
BolC04.GH3-11.b    ............................................................
BolCX.GH3-11.c     ............................................................
BolC03.GH3-12.a    ............................................................
BolC09.GH3-12.b    ............................................................
BolC09.GH3-14.a    ............................................................
BolC03.GH3-15.a    ............................................................
BolCX.GH3-16.a     ............................................................
BolC07.GH3-17.a    ............................................................
BolC08.GH3-17.b    ............................................................
BolC08.GH3-19.a    ............................................................
BnaA09R.GH3-1.a    ............................................................
BnaCX.GH3-1.a      ............................................................
BnaA01.GH3-2.a     ............................................................
BnaC01.GH3-2.a     ............................................................
BnaA09.GH3-3.a     ............................................................
BnaC08.GH3-3.a     ............................................................
BnaA01.GH3-5.a     ............................................................
BnaA03.GH3-5.b     ............................................................
BnaC01.GH3-5.a     ............................................................
BnaC07.GH3-5.b     ............................................................
BnaA03.GH3-6.a     ............................................................
BnaAX.GH3-6.b      ............................................................
BnaC03.GH3-6.a     ............................................................
BnaCX.GH3-6.b      ............................................................
BnaA06.GH3-7.a     ............................................................
BnaA08.GH3-7.b     ............................................................
BnaC06.GH3-7.a     ............................................................
BnaA03.GH3-8.a     ............................................................
BnaA03.GH3-8.b     ............................................................
BnaA03.GH3-8.c     ............................................................
BnaA06.GH3-8.d     ............................................................
```

```
BnaA06.GH3-8.e       ............................................................
BnaA09.GH3-8.f       ............................................................
BnaA09.GH3-8.g       ............................................................
BnaAX.GH3-8.h        ............................................................
BnaAX.GH3-8.i        ............................................................
BnaC01.GH3-8.a       ............................................................
BnaC02.GH3-8.b       ............................................................
BnaC02.GH3-8.c       ............................................................
BnaC02.GH3-8.d       ............................................................
BnaC07.GH3-8.e       ............................................................
BnaC09.GH3-8.f       ............................................................
BnaCX.GH3-8.g        ............................................................
BnaA05.GH3-9.a       ............................................................
BnaC04.GH3-9.a       ............................................................
BnaA03.GH3-10.a      ............................................................
BnaA09.GH3-10.b      ............................................................
BnaC09.GH3-10.a      ............................................................
BnaA03.GH3-11.a      ............................................................
BnaA04R.GH3-11.b     ............................................................
BnaA05.GH3-11.c      ............................................................
BnaC04.GH3-11.a      MCMKKELQVRNKSEIMHQKLTRELCESNHCSTKSLKDFENERKEHVVEELCDEFAKAVEN
BnaCX.GH3-11.b       ............................................................
BnaA02.GH3-12.a      ............................................................
BnaA03.GH3-12.b      ............................................................
BnaA06.GH3-12.c      ............................................................
BnaA10.GH3-12.d      ............................................................
BnaC02.GH3-12.a      ............................................................
BnaC03.GH3-12.b      ............................................................
BnaC09.GH3-12.c      ............................................................
BnaA10.GH3-14.a      ............................................................
BnaC09.GH3-14.a      ............................................................
BnaA03.GH3-15.a      ............................................................
BnaC03.GH3-15.a      ............................................................
BnaAX.GH3-16.a       ............................................................
BnaC02.GH3-16.a      ............................................................
BnaA07.GH3-17.a      ............................................................
BnaA08.GH3-17.b      ............................................................
BnaA09.GH3-17.c      ............................................................
BnaC08.GH3-17.a      ............................................................
BnaCX.GH3-17.b       ............................................................
BnaCX.GH3-17.c       ............................................................
BnaA05GH3-18.a       ............................................................
BnaCX.GH3-18.a       ............................................................
BnaA06.GH3-19.a      ............................................................
BnaA09.GH3-19.b      ............................................................
consensus>70         ............................................................
```

```
                                                               1        10
AtGH3-1          ..............................................MAVDSNLSSPL.GP
AtGH3-2          ..............................................MAVDSPLQSRMVSA
AtGH3-3          ..............................................MTVDSALRSPMMHS
AtGH3-4          ..............................................MAVDSLLQSGMASP
AtGH3-5          .............................................MPEAPKKESLEVFDL
AtGH3-6          .............................................MPEAPKIAALEVSDE
AtGH3-7          .......................................................MSLTS
AtGH3-8          .........................................................MSL
AtGH3-9          ......................................................MDVMK.
AtGH3-10         ..........................................................ME
AtGH3-11         .............................................MDALKHKRAFKMLEK
AtGH3-12         ..........................................................MK
AtGH3-13         ........................................................MLPK
AtGH3-14         EKNTKSGNSSTILRHRTFRQTTHFVCFLLRQGKWPELIYFARTKQSLVSQKAAQAIMLPK
AtGH3-15         ........................................................MLPK
AtGH3-16         ..........................MTPFICTERERDGSDPIHCSQRKDNRKAAIMLPK
AtGH3-17         ........................................................MIPS
AtGH3-18         ............................................................
AtGH3-19         ............................................................
BraX.GH3-1.a     ..............................................MAVDSSLSSPL.GP
BraX.GH3-2.a     ..............................................MAVDSPLQSR....
BraA09.GH3-3.a   ..............................................MIVDLALKSPMIHT
BraA01.GH3-5.a   .............................................MPEAPKNESLEVFDL
BraA03.GH3-5.b   .............................................MPEAPKYESLDAFDL
BraA02.GH3-6.a   .............................................MPEAPKIEALEVSDQ
BraA03.GH3-6.b   .............................................MPEAPELA...VSDQ
BraA06.GH3-7.a   .........................................................MSI
BraA08.GH3-7.b   .......................................................MSVIS
BraA02.GH3-8.a   .........................................................MSL
BraA03.GH3-8.b   .........................................................MSM
BraA03.GH3-8.c   ...........................................................M
BraA03.GH3-8.d   ..........................................................MN
BraA06.GH3-8.e   ............................................................
BraA06.GH3-8.f   ............................................................
BraA09.GH3-8.g   ............................................................
BraA09.GH3-8.h   ............................................................
BraA10.GH3-8.i   ............................................................
BraA09.GH3-10.a  ........................................MLGLRTLLVFCIAVVPSLME
BraA03.GH3-11.a  ................MGAKSKTKQTFSFEITPKLGDVDYIYFLYG........RMLEKK
BraA04.GH3-11.b  .......................................................MLEKK
BraA05.GH3-11.c  .......................................................MLEKK
BraA02.GH3-12.a  ..........................................................MN
BraA03.GH3-12.b  ..........................................................MK
BraA06.GH3-12.c  .........................................................MSI
BraA10.GH3-12.d  ..........................................................MK
BraA10.GH3-14.a  ........................................................MLPK
BraA03.GH3-15.a  ..........................................MVIGCGLPSTMAAIMLPK
BraA07.GH3-15.b  ........................................................MLTK
BraA02.GH3-16.a  ........................................................MLPR
BraA07.GH3-17.a  ............................................................
BraA09.GH3-17.b  ........................................................MIPS
BraAX.GH3-17.c   .......................................................MMIPS
BraA04.GH3-19.a  ............................................................
BraA06.GH3-19.b  ............................................................
BraA09.GH3-19.c  ............................................................
BraA09.GH3-19.d  ............................................................
BraAX.GH3-19.e   ............................................................
BolC09.GH3-1.a   ..............................................MAVDSSLSSPL.GP
BolC01.GH3-2.a   ..............................................MAVDSPLQSR....
BolC08.GH3-3.a   ..............................................MIVDLALKSPMIHS
BolC01.GH3-5.a   .............................................MPEAPKNESLEVFDL
BolC07.GH3-5.b   .............................................MPEAPKYESLDAFDL
BolC02.GH3-6.a   .............................................MPEAPKIEALEVSDQ
BolC03.GH3-6.b   .............................................MPEAPELAVSELSDQ
BolC06.GH3-7.a   .........................................................MSI
BolC01.GH3-8.a   ...........................................................M
BolC03.GH3-8.b   .........................................................MPC
BolC07.GH3-8.c   ...........................................................M
BolC08.GH3-8.d   ............................................................
BolC09.GH3-8.e   ............................................................
BolC09.GH3-10.a  ..........................................................ME
BolC03.GH3-11.a  .......................................................MLEKK
BolC04.GH3-11.b  .......................................................MLEKK
BolCX.GH3-11.c   .......................................................MLEKK
BolC03.GH3-12.a  ..........................................................MK
BolC09.GH3-12.b  ..........................................................MN
BolC09.GH3-14.a  ........................................................MLPK
BolC03.GH3-15.a  ........................................................MLPK
BolCX.GH3-16.a   ........................................................MLPR
BolC07.GH3-17.a  ........................................................MIPS
BolC08.GH3-17.b  ........................................................MIPS
BolC08.GH3-19.a  .................................................MVGVSGLQTSK
BnaA09R.GH3-1.a  ..............................................MAVDSSLSSPL.GP
BnaCX.GH3-1.a    ..............................................MAVDSSLSSPL.GP
BnaA01.GH3-2.a   ..............................................MAVDSPLQSR....
BnaC01.GH3-2.a   ..............................................MAVDSPLQSR....
BnaA09.GH3-3.a   ..............................................MIVDLALKSPMIHT
BnaC08.GH3-3.a   ..............................................MIVDLALKSPMIHS
BnaA01.GH3-5.a   .............................................MPEAPKNESLEVFDL
BnaA03.GH3-5.b   .............................................MPEAPKYESLDAFDL
BnaC01.GH3-5.a   .............................................MPEAPKNESLEVFDL
BnaC07.GH3-5.b   .............................................MPEAPKYESLDAFDL
BnaA03.GH3-6.a   .............................................MPEAPELAVSEVSDQ
BnaAX.GH3-6.b    .............................................MPEAPKIEAFEVSDQ
BnaC03.GH3-6.a   .............................................MPEAPELAVSELSDQ
BnaCX.GH3-6.b    .............................................MPEAPKIEALEVSDQ
BnaA06.GH3-7.a   .........................................................MSI
BnaA08.GH3-7.b   .......................................................MSVIS
BnaC06.GH3-7.a   .........................................................MSI
BnaA03.GH3-8.a   .........................................................MSM
BnaA03.GH3-8.b   ...........................................................M
BnaA03.GH3-8.c   ..........................................................MN
BnaA06.GH3-8.d   ............................................................
```

```
BnaA06.GH3-8.e      ............................................................
BnaA09.GH3-8.f      ............................................................
BnaA09.GH3-8.g      ............................................................
BnaAX.GH3-8.h       ............................................................
BnaAX.GH3-8.i       ............................................................
BnaC01.GH3-8.a      ...........................................................M
BnaC02.GH3-8.b      .........................................................MSL
BnaC02.GH3-8.c      .........................................................MSL
BnaC02.GH3-8.d      .........................................................MSL
BnaC07.GH3-8.e      ...........................................................M
BnaC09.GH3-8.f      ............................................................
BnaCX.GH3-8.g       ..........................................................MS
BnaA05.GH3-9.a      ......................................................MDVVKI
BnaC04.GH3-9.a      ......................................................MDVLKF
BnaA03.GH3-10.a     ..........................................................ME
BnaA09.GH3-10.b     ..........................................................ME
BnaC09.GH3-10.a     ..........................................................ME
BnaA03.GH3-11.a     ................MGAKSKTKQTFSFDIKLGRNLATSITFTFSMVARSNNIRMLEKK
BnaA04R.GH3-11.b    ...................................................NNIRMLEKK
BnaA05.GH3-11.c     .......................................................MLEKK
BnaC04.GH3-11.a     YEDKEMSEQTKFSNSLFFFSLYKIATSSDFSESLKICFLYLSPPPSFIARSNNIRMLEKK
BnaCX.GH3-11.b      .......................................................MLEKK
BnaA02.GH3-12.a     ..........................................................MD
BnaA03.GH3-12.b     ..........................................................MK
BnaA06.GH3-12.c     .........................................................MSI
BnaA10.GH3-12.d     ..........................................................MK
BnaC02.GH3-12.a     ..........................................................MN
BnaC03.GH3-12.b     ..........................................MLLKSSLTLKNKREASMK
BnaC09.GH3-12.c     ..........................................................MN
BnaA10.GH3-14.a     ........................................................MLPK
BnaC09.GH3-14.a     ........................................................MLPK
BnaA03.GH3-15.a     ..........................................MVIGCGLPSTMAAIMLPK
BnaC03.GH3-15.a     ..........................................MVIGCDLPSTMSAIMLPK
BnaAX.GH3-16.a      .........NYSVTGTRTFRHTTPFVCFIQYLLNLKSPSFFFCYCRTLQIIKNQQTMLPR
BnaC02.GH3-16.a     ........................................................MLPR
BnaA07.GH3-17.a     ........................................................MIPS
BnaA08.GH3-17.b     ........................................................MIPS
BnaA09.GH3-17.c     ........................................................MIPS
BnaC08.GH3-17.a     ............................................................
BnaCX.GH3-17.b      ........................................................MIPS
BnaCX.GH3-17.c      ............................................................
BnaA05GH3-18.a      ............................................................
BnaCX.GH3-18.a      ............................................................
BnaA06.GH3-19.a     ............................................................
BnaA09.GH3-19.b     .................................................MVGVSVLQTSK
consensus>70        ............................................................
```

```
                       20                         30        40        50
                        .                          .         .         .
AtGH3-1           PACEKDAKALRFIE.................EMTRNADTVQENLLAEILARNADTEYLRR
AtGH3-2           TTSEKDVKALKFIE.................EMTRNPDSVQEKVLGEILTRNSNTEYLKR
AtGH3-3           P.STKDVKALRFIE.................EMTRNVDFVQKKVIREILSRNSDTEYLKR
AtGH3-4           TTSETEVKALKFIE.................EITRNPDSVQEKVLGEILSRNSNTEYLKR
AtGH3-5           TLDQKNKQKLQLIE.................ELTSNADQVQRQVLEEILTRNADVEYLRR
AtGH3-6           SLAEKNKNKLQFIE.................DVTTNADDVQRRVLEEILSRNADVEYLKR
AtGH3-7           DLSEKSSDKMKVLE.................DLTSNVTQIQDNVLEEILTLNANTNYLQK
AtGH3-8           CSDLTEKLDEEILE.................DLTSNVKQIQDNVLEEILTLNANTEYLRR
AtGH3-9           ...LDHDSVLKELE.................RITSKAAEVQDNILRGILERNKDTEYLSK
AtGH3-10          TVEAGHDDVIGWFE.................HVSENACKVQSETLRRILELNSGVEYLRK
AtGH3-11          VETFDMNRVIDEFD.................EMTRNAHQVQKQTLKEILLKNQSAIYLQN
AtGH3-12          PIFDINETFEKQLK.................DLTSNVKSIQDNLLEEIITPNTKTEYLQR
AtGH3-13          FDLTDPKASLSLLE.................DVTTNVTQIQDSILEAVLSRNAHTEYLKG
AtGH3-14          FDPTDQKACLSLLE.................DVTTNVKQIQDSVLEAILSRNAHTEYLSG
AtGH3-15          FDPTNQKACLSLLE.................DLTTNVKQIQDSVLEAILSRNAQTEYLRG
AtGH3-16          FDPTNPLATMSVLE.................DVTTNVKKIQDSLLEAILSRNSQTEYLRG
AtGH3-17          YDPNDTEAGLKLLE.................DLTTNAEAIQQQVLHQILSQNSGTQYLRA
AtGH3-18          ..MMNPSLNLMDLE.................ELTSNAKQIQEDVLEEILTLNANTEYLHR
AtGH3-19          ...MSLSVELKDLE.................VLTTNAKQIQDDVLKEILTLNANTEYLKR
BraX.GH3-1.a      PACEKDAKALRFIE.................EMTRNADTVQENLLAEILRRNAETEYLRR
BraX.GH3-2.a      TTSEKDVKALSFIE.................EMTRNPDSVQEKVLGEILSRNSGTEYLKR
BraA09.GH3-3.a    PQSDKDLKALRFIE.................EMTRKVDFVQKKVIKEILSRNSETEYLKR
BraA01.GH3-5.a    TLDEKNKRRLQLIE.................ELTSNADQVQRRVLEEILTRNADVEYLRR
BraA03.GH3-5.b    TLDEKNKRKLQLIE.................ELTSNADQVQKRVLEEILIRNADVEYLRR
BraA02.GH3-6.a    TLAEKNKNRLQFIE.................EVTSNADDVQRRVLEEILSRNADVEYLKR
BraA03.GH3-6.b    ILEEKNKNKLQFIE.................EVTSNADEVQRQVLEEILSRNADVEYLKR
BraA06.GH3-7.a    ISYYNEEEKGKVLE.................DLTWNVKQIQDDLLKEILTLNSGTEYLQN
BraA08.GH3-7.b    VLTEKANDKLKILE.................DLTSNVKQIQDNVLEEILTLNTNTEYLQR
BraA02.GH3-8.a    CSDLCDKLDENVLE.................ELASNVKQVQDNVLEEILTLNAGTEYLRR
BraA03.GH3-8.b    WADLSDKLDEKVLE.................DLTSNVKQMQDDVLKEILTLNANTEYLRP
BraA03.GH3-8.c    INLTEEDAGLAMLE.................KLTSNVKQIQDAVLKEILTCDANTEYLRS
BraA03.GH3-8.d    NLTKEEEAGLAVLE.................NLTSNVKQIQDEVLKEILTCDANTEYLRS
BraA06.GH3-8.e    ...MSLGCDLTLLE.................ELTSNAKQIQEDVLNKILKANANTEYLQR
BraA06.GH3-8.f    ...MSLDCDLTVLE.................ELTSNAKQIQDDVLTKILKANANTEYLQR
BraA09.GH3-8.g    ..........MELM.................NLTSNVKQIQDDVLKEILTLNANTEYLRG
BraA09.GH3-8.h    ...MSLGCDLSVLE.................ELTSNAKQIQDDVLTKILKANTNTEYLSR
BraA10.GH3-8.i    ..........MDLK.................KLTSNVKHIQDDVLKEILTLNAKTEYLRG
BraA09.GH3-10.a   PAEAGHADVIGWFE.................HVSEKACKAQRETLRRILELNSRVEYLRK
BraA03.GH3-11.a   VETFNMNRVIDEFE.................EMTRNADQVQKQTLKEILHKNQSAIYLQN
BraA04.GH3-11.b   VETFNMNRVIEDFE.................EMSRNADQVQIQTLKDILLKNHSAIYLKN
BraA05.GH3-11.c   AETFNMNRVIDDFD.................EMTRNADQVQKQTLKEILHKNKSAIYLRN
BraA02.GH3-12.a   PNSDNNQRWEAKLK.................DLTSNVQQIQDNLLEEILTPNLNTEYLQR
BraA03.GH3-12.b   PISNNNERWEEKLK.................DLTFNVKEIQDKLLEEILTPNLKTEYLQR
BraA06.GH3-12.c   ISDFNDEEKEKVLE.................DLTSNVKQIQDDLLKEILTLNSGTEYLQS
BraA10.GH3-12.d   PISNNDETWDATLK.................DLTSNVKEIQDNVLEEIITPNLKTEYLQR
BraA10.GH3-14.a   FDPANHKACLSVLE.................DVTTNAKQIQDSVLEAILSRNAKTEYLRG
BraA03.GH3-15.a   FDPTNSKACLSILE.................DVTTNAKQVQDSVLEAILSRNAQTEYLKG
BraA07.GH3-15.b   FDPTNQKACLSLLE.................DITSNANQIQDSVLEAILTRNAHTEYLRG
BraA02.GH3-16.a   FDPTNPLACMSVLE.................DVTSNAKQIQDSVLEAILSRNAQTEYLKG
BraA07.GH3-17.a   ............................................................
BraA09.GH3-17.b   YDPNDTEAGLKLLE.................DLTTNAEVIQEQVLHQILSQNCETQYLQA
BraAX.GH3-17.c    YDPNDTEAGLQLLE.................DLTTNADAIQQQVLHQILSQNSETEYLRS
BraA04.GH3-19.a   ...MNQNSELKALE.................KLSSNAKQIQEDMLEEILRSSANTEYLRR
BraA06.GH3-19.b   ...MSLGCDLTVLE.................ELTSNAKQIQDDVLNKILNANANTEYLKR
BraA09.GH3-19.c   ...MSLGCDLSVLE.................ELTSNAKQIQDDVLTKILKANANTEYLSR
BraA09.GH3-19.d   ...MRLGCDLSVLE.................ELTSNAKQIQDDVLNKILKANANTEYLSR
BraAX.GH3-19.e    ...MSSIVELSSLE.................ELTLNAEQIQDDLLEKILRLNSNTEYLRR
BolC09.GH3-1.a    PACEKDAKALRFIE.................EMTRNADTVQENLLAEILRRNAETEYLRR
BolC01.GH3-2.a    TTSEKDAKALSFIE.................EMTRNPDSVQEKVLGEILSRNSGTEYLKR
BolC08.GH3-3.a    PQSDKDLKALRFIE.................EMTRNVDFVQKKVIKEILSRNSETEYLKR
BolC01.GH3-5.a    TLDEKNKRRLQLIE.................ELTSNADQVQRRVLEEILTRNADVEYLKR
BolC07.GH3-5.b    TLDEKNKRKLQLIE.................ELTSNADQVQRRVLEEILTRNADVEYLRR
BolC02.GH3-6.a    TLAEKNKNRLQFIE.................EVTSNADDVQRRVLEEILSRNADVEYLKR
BolC03.GH3-6.b    TLAEKNKNKLQFIE.................EVTSNADDVQRRVLEEILSRNADVEYLKR
BolC06.GH3-7.a    ISYYNEEEKGKVLE.................DLTWNVKQIQDDLLKEILTLNSGTEYLQS
BolC01.GH3-8.a    NTLTDKDVAMSLLE.................DLTSNVKQIQDEVLEEILRCDANTEYLQS
BolC03.GH3-8.b    LHDAIDVNKIRVIEFPIAENAKRQIRREGFGRLDVQCQQIQDDVLKEILTLSANTEYLRP
BolC07.GH3-8.c    INLTEEDAGLAVLE.................KLTSNVKQIQDAVLKEILTCDANTEYLRS
BolC08.GH3-8.d    ...MSQGCDLSILE.................ELTSNAKKRQDNVLNKILKANANTEYLSR
BolC09.GH3-8.e    ..................................................MPEQNLYL..
BolC09.GH3-10.a   PAEAGHADVIGWFE.................HVSEKACKAQRETLRRILELNSGVEYLRK
BolC03.GH3-11.a   VENFNMNRVIDEFE.................EMTRNADQVQKQTLKEILHKNKSAIYLQN
BolC04.GH3-11.b   FETFNMNRVIEDFE.................EMSRNADQVQIQTLKDILLKSQSAIYLKN
BolCX.GH3-11.c    AETFNMNRVIDEFD.................EMTRNADQVQKQTLKEILHKNKSAIYLRN
BolC03.GH3-12.a   PISNNNERWEEKLK.................DLSFNVKQIQDNLLEEILTPNLKTEYLQR
BolC09.GH3-12.b   PNSDNNQRWEAKLK.................DLTSNVQQIQDNLLKEILTPNLNTEYLQR
BolC09.GH3-14.a   FDPANHKACLSVLE.................DVTTNAKQIQDSVLEAILSRNAKTEYLRG
BolC03.GH3-15.a   FDPTNSKACLSILE.................DVTTNAKQVQDSVLEAILSRNAQTEYLKG
BolCX.GH3-16.a    FDPTNPLACMSVLE.................DVTSNAKQIQDSVLEAILSRNAQTEYLKG
BolC07.GH3-17.a   YDPNDTEAGLKLLE.................DLTSNAEAIQEQVLHEILSQNSGTQYLRA
BolC08.GH3-17.b   YDPNDTEAGLKLLE.................DLTTNADAIQQQVLHQILSQNSETEYLRS
BolC08.GH3-19.a   IANMSQGCDLSVLE.................ELTSNAKKIQDDVLTKILKANANTEYLSL
BnaA09R.GH3-1.a   PACEKDAKALRFIE.................EMTRNADTVQENLLAEILRRNAETEYLRR
BnaCX.GH3-1.a     PACEKDAKALRFIE.................EMTRNADTVQENLLAEILRRNAETEYLRR
BnaA01.GH3-2.a    TTSEKDVKALSFIE.................EMTRNPDSVQEKVLGEILSRNSGTEYLKR
BnaC01.GH3-2.a    TTSEKDAKALSFIE.................EMTRNSDSVQEKVLGEILSRNSGTEYLKR
BnaA09.GH3-3.a    PQSDKDLKALRFIE.................EMTRNVDFVQKKVIKEILSRNSETEYLKR
BnaC08.GH3-3.a    PQSDKDLKALRFIE.................EMTRNVDFVQKKVIKEILSRNSETEYLKR
BnaA01.GH3-5.a    TLDEKNKRRLQLIE.................ELTSNADQVQRRVLEEILTRNADVEYLRR
BnaA03.GH3-5.b    TLDEKNKRKLQLIE.................ELTSNADQVQKRVLEEILIRNADVEYLRR
BnaC01.GH3-5.a    TLDEKNKRRLQLIE.................ELTSNADQVQRRVLEEILTRNADVEYLKR
BnaC07.GH3-5.b    TLDEKNKRKLQLIE.................ELTSNADQVQRRVLEEILTRNADVEYLRR
BnaA03.GH3-6.a    ILEEKNKNKLQFIE.................EVTSNADEVQRQVLEEILSRNADVEYLKR
BnaAX.GH3-6.b     TLAEKNKNRLQFIE.................EVTSNADDVQRRVLEEILSRNADVEYLKR
BnaC03.GH3-6.a    SLAEKNKNKLQFIE.................EVTSNADDVQRRVLEEILSRNADVEYLKR
BnaCX.GH3-6.b     TLAEKNKNRLQLIE.................EVTSNADDVQRRVLEEILSRNADVEYLKR
BnaA06.GH3-7.a    ISYYNEEEKGKVLE.................DLTWNVKQIQDDLLKEILTLNSGTEYLQN
BnaA08.GH3-7.b    VLTEKANDKLKILE.................DLTSNVKQIQDNVLEEILTLNTNTEYLQR
BnaC06.GH3-7.a    ISYYNEEEKGKVLE.................DLTWNVKQIQDDMLKEILTLNSGTEYLQN
BnaA03.GH3-8.a    WADLSDKLDEKVLE.................DLTSNVKQMQDDVLKEILTLNANTEYLRP
BnaA03.GH3-8.b    INLTEEDAGLAMLE.................KLTSNVKQIQDAVLKEILTCDANTEYLRS
BnaA03.GH3-8.c    NLTKEEEAGLAVLE.................NLTSNVKQIQDEVLKEILTCDANTEYLRS
BnaA06.GH3-8.d    ...MNLGCDLTVLE.................ELTSNARQIQDDVLNKILKANANTEYLQR
```

```
BnaA06.GH3-8.e      ...MSLDCDLTVLE.................ELTSNAKQIQDDVLTKILKANANTEYLQR
BnaA09.GH3-8.f      ...MSLSCDLSVLE.................ELTSNAKQIQDDVLTKILKANANTEYLSR
BnaA09.GH3-8.g      ...MSQRCDLSVLE.................ELTSNAKQIQDDVLTKILKSSANTEYLSR
BnaAX.GH3-8.h       ...............................................................
BnaAX.GH3-8.i       ...MSQGCDLSVLE.................ELTSNAKQIQDDVLNKILKANANTEYLSR
BnaC01.GH3-8.a      NTLTDKDVAMSLLE.................DLTSNVKQIQDEVLEEILRCDANTEYLQS
BnaC02.GH3-8.b      CSDLCDKLDENVLE.................ELTSNVKQVQDNVLEEILTLNAGTEYLRR
BnaC02.GH3-8.c      CSDLCDKLDENVLE.................ELTSNVKQVQDNVLEEILTLNAGTEYLRR
BnaC02.GH3-8.d      CSDLCDKLDENVLE.................ELTSNVKQVQDNVLEEILTLNAGTEYLRR
BnaC07.GH3-8.e      INLTEEDAGLAVLE.................KLTSNVKQIQDAVLKEILTCDANTEYLRS
BnaC09.GH3-8.f      ..........MELM.................KLTSNVKQIQDDVLKEILTLNANTEYLRG
BnaCX.GH3-8.g       RTNMSLDCDLTVLE.................ELTSNAKQIQDDVLTKILKANANTVYLGR
BnaA05.GH3-9.a      DYNYKGDNALKELE.................RITSKAAEVQDNILCGILEQNKDTQYLSK
BnaC04.GH3-9.a      DYNHKGDNALKELE.................RITSKAAEVQDNMLSGILERNKDTQYLRK
BnaA03.GH3-10.a     TAEAGHADVIGWFE.................HVSENACKAQRETLRRILELNCGVDYLRK
BnaA09.GH3-10.b     PAEAGHADVIGWFE.................HVSEKASKAQSETLRRILELNSRVEYLRK
BnaC09.GH3-10.a     PAEAGHADVIGWFE.................HVSEKACKTQRETLRRILELNSGVEYLRK
BnaA03.GH3-11.a     VETFNMNRVIDEFE.................EMTRNADQVQKQTLKEILHKNQSAIYLQN
BnaA04R.GH3-11.b    VETFNMNRVIEDFE.................EMSRNADQVQIQTLKDILLKNHSAIYLKN
BnaA05.GH3-11.c     AETFNMNRVIDDFD.................EMTRNADQVQKQTLKEILHKNESAIYLRN
BnaC04.GH3-11.a     FETFNMNRVIEDFE.................EMSRNADQVQIQTLKDILLKSQSAIYLKN
BnaCX.GH3-11.b      VENFNMNRVIDEFE.................EMTRNADQVQKQTLKEILHKNKSAIYLQN
BnaA02.GH3-12.a     PNSDNNQRWEAKLK.................DLTSNVQQIQDNLLEEILTPNLNTEYLQR
BnaA03.GH3-12.b     PISNNNERWEEKLK.................DLTFNVKEIQDKLLEEILTPNLKTEYLQR
BnaA06.GH3-12.c     ISDFNDEEKEKVLE.................DLTSNVKQIQDDLLKEILTLNSGTEYLQS
BnaA10.GH3-12.d     PISNNDETWDATLK.................DLTSNVKETQDNVLEEIITPNLKTEYLQR
BnaC02.GH3-12.a     PNSDNNQRWEAKLK.................DLTSNVQQIQDSLLEEILTPNLNTEYLQR
BnaC03.GH3-12.b     PISNNNERWEEKLK.................DLSFNVKQIQDNLLEEILTPNLKTEYLQR
BnaC09.GH3-12.c     PNSDNNQRWEAKLK.................DLTSNVQQIQDNLLKEILTPNLNTEYLQR
BnaA10.GH3-14.a     FDPANHKACLSVLE.................DVTTNAKQIQDSVLEAILSRNAKTEYLRG
BnaC09.GH3-14.a     FDPANHKACLSVLE.................DVTTNAKQIQDSVLEAILSRNAKTEYLRG
BnaA03.GH3-15.a     FDPTNSKACLSILE.................DVTTNAKQVQDSVLEAILSRNAQTEYLKG
BnaC03.GH3-15.a     FDPTNSKACLSILE.................DVTTNAKQVQDSVLEAILSRNAQTEYLKG
BnaAX.GH3-16.a      FDPTNPLACMSVLE.................DVTSNAKQIQDSVLEAILSRNAQTEYLKG
BnaC02.GH3-16.a     FDPTNPLACMSVLE.................DVTSNAKQIQDSVLEAILSRNAQTEYLKG
BnaA07.GH3-17.a     YDPNDTEAGLKLLE.................DLTSNAEAIQEQVLHEILSQNSGTQYLRA
BnaA08.GH3-17.b     YDPNDTEAGLQLLE.................DLTTNADAIQQQVLHQILSQNSETEYLRS
BnaA09.GH3-17.c     YDPNDTEAGLKLLE.................DLTTNAEVIQEQVLHQILSQNCETQYLQA
BnaC08.GH3-17.a     ...............................................................
BnaCX.GH3-17.b      YDPNDTEAGLKLLE.................DLTSNAEAIQEQVLHEILSQNSGTQYLRA
BnaCX.GH3-17.c      ...............................................................
BnaA05GH3-18.a      ...MSSIVELSSLE.................ELTLNAEQIQDDLLEKILRLNSNTEYLRR
BnaCX.GH3-18.a      ...MSQGCDLSVLE.................ELTSNAKQIQDDVLNKILKANANTEYLSR
BnaA06.GH3-19.a     ...MSLGCDLTVLE.................ELTSNAKQIQDDVLNKILKANANTEYLKR
BnaA09.GH3-19.b     IANMSLGCDLSVLE.................ELTSNAKQIQDDVLTKILKANANTEYLSR
consensus>70        .............e.................elt.n..qiq...l..il..n..teyl..
```

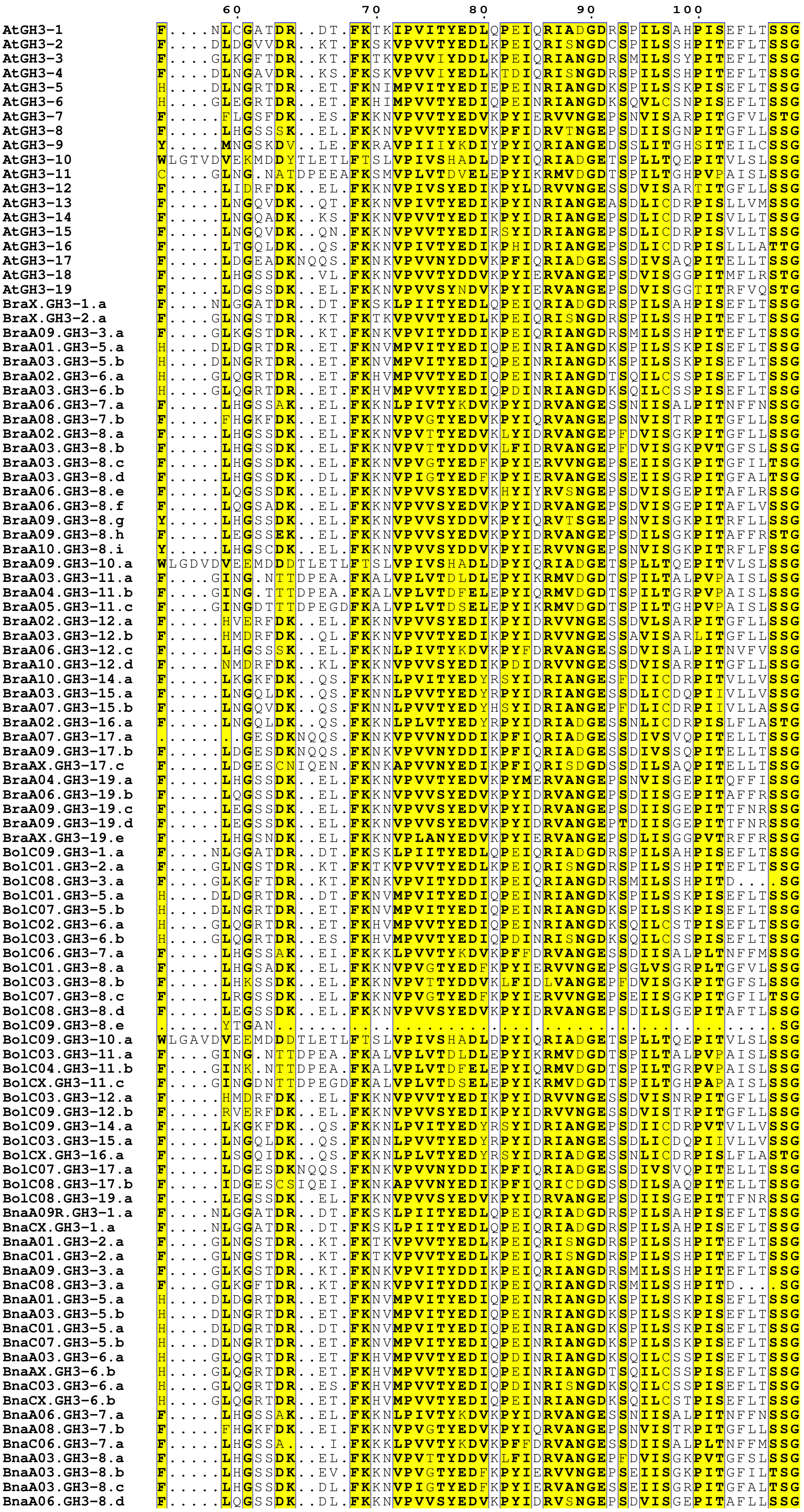

```
BnaA06.GH3-8.e      F.....LQGSADK..EL.FKKNVPVVSYEDVKPYIDRVANGEPSDVISGEPITAFLVSSG
BnaA09.GH3-8.f      F.....LEGSSEK..EL.FKKNVPVVSYEDVKPYIDRVANGEPSDILSGKPITAFFRSTG
BnaA09.GH3-8.g      F.....LEGSSEK..EL.FKKNVPVVSYEDVKPYIDRIANGEPSDILSGEPITAFILSSG
BnaAX.GH3-8.h       ............................................................
BnaAX.GH3-8.i       F.....LEGSSDK..EL.FKKNVPVVSYEDVKPYIDRVANGEPSDILSGEPITAFTLSSG
BnaC01.GH3-8.a      F.....LHGSADK..EL.FKKNVPVGTYEDFKPYIERVVNGEPSGLVSGRPLTGFVLSSG
BnaC02.GH3-8.b      F.....LHGSSDK..EL.FKKNVPVTTYEDVKLYIGRVANGEPFDVISGKPITGFLLSSG
BnaC02.GH3-8.c      F.....LHGSSDK..EL.FKKNVPVTTYEDVKLYIGRVANGEPFDVISGKPITGFLLSSG
BnaC02.GH3-8.d      F.....LHGSSDK..EL.FKKNVPVTTYEDVKLYIGRVANGEPFDVISGKPITGFLLSSG
BnaC07.GH3-8.e      F.....LRGSSDK..EL.FKKNVPVGTYEDFKPYIERVVNGEPSEIISGKPITGFILTSG
BnaC09.GH3-8.f      Y.....LHGAYDK..EL.FKKNVPVVSYDDVKPYIERVANGEPSNVISGKPITRFLLSSG
BnaCX.GH3-8.g       F.....LQGSADK..EL.FKKNVPVVSYEDLKPYLDRVANGEPSDVISGEPITAFLVSSG
BnaA05.GH3-9.a      Y.....MKGSKDV..LE.FKRSVPIITYKDVCPYIQRIANGEDSSLITGHLITEILCSSG
BnaC04.GH3-9.a      Y.....MKGSKDV..ME.FKRSVPVITYKDVCPYIQRIANGEDSSLITGHPITEILCSSG
BnaA03.GH3-10.a     WLGSVDVKEIDDNSLETLFTSLVPIVSHADMDSYIQRIANGETSPLLTQEPITVLSLSSG
BnaA09.GH3-10.b     WLGDVDVEEMDDDTLETLFTSLVPIVSHADLDPYIQRIADGETSPLLTQEPITVLSLSSG
BnaC09.GH3-10.a     WLGAVDVEEMDDDTLETLFTSLVPIVSHADLDPYIQRIADGETSPLLTQEPITVLSLSSG
BnaA03.GH3-11.a     F....GING.NTTDPEA.FKALVPLVTDLDLEPYIKRMVDGDTSPILTALPVPAISLSSG
BnaA04R.GH3-11.b    F....GING.TTTDPEA.FKALVPLVTDFELEPYIQRMVDGDTSPILTGRPVPAISLSSG
BnaA05.GH3-11.c     F....GINGDTTTDPEGDFKALVPLVTDSELEPYIKRMVDGDTSPILTGHPVPAISLSSG
BnaC04.GH3-11.a     F....GINK.NTTDPEA.FKALVPLVTDFELEPYIQRMVDGDTSPILTGRPVPAISLSSG
BnaCX.GH3-11.b      F....GING.NTTDPEA.FKALVPLVTDLDLEPYIKRMVDGDTSPILTALPVPAISLSSG
BnaA02.GH3-12.a     F.....HVERFDK..EL.FKKNVPVVSYEEIKPYIDRVVNGESSDVLSARPITGFLLSSG
BnaA03.GH3-12.b     F.....HMDRFDK..QL.FKKNVPVVTYEDIKPYIDRVVNGESSAVISARLITGFLLSSG
BnaA06.GH3-12.c     F.....LHGSSSK..EL.FKKNLPIVTYKDVKPYFDRVANGESSDVISALPITNVFVSSG
BnaA10.GH3-12.d     F.....NMDRFDK..KL.FKKNVPVVSYEDIKPDIDRVVNGESSDVISARPITGFLLSSG
BnaC02.GH3-12.a     F.....HVERFDK..EL.FKKNVPVVSYEDIKPYIDRVVNGETSEVLSARPITGFLLSSG
BnaC03.GH3-12.b     F.....HMDRFDK..EL.FKKNVPVVTYEDIKPYIDRVVNGESSDVISNRPITGFLLSSG
BnaC09.GH3-12.c     F.....RVERFDK..EL.FKKNVPVVSYEDIKPYIDRVVNGESSDVISTRPITGFLLSSG
BnaA10.GH3-14.a     F.....LKGKFDK..QS.FKKNLPVITYEDYRSYIDRIANGESSDIICDRPITVLLVSSG
BnaC09.GH3-14.a     F.....LKGKFDK..QT.FKKNLPVITYEDYRSYIDRIANGEPSDIICDRPVTVLLVSSG
BnaA03.GH3-15.a     F.....LNGQLDK..QS.FKNNLPVVTYEDYRPYIDRIANGESSDLICDQPIIVLLVSSG
BnaC03.GH3-15.a     F.....LNGQLDK..QS.FKNNLPVVTYEDYRPYIDRIANGESSDLICDQLIIVLLVSSG
BnaAX.GH3-16.a      F.....LNGQLDK..QS.FKKNLPLVTYEDYRPYIDRIADGESSNLICDRPISLFLASTG
BnaC02.GH3-16.a     F.....LSGHIDK..QS.FKKNLPLVTYEDYRSYINRIADGESSNLICDRPISLFLASTG
BnaA07.GH3-17.a     F.....LDGESDKNQQS.FKNKVPVVNYDDIKPFIQRIADGESSDIVSVQPITELLTSSG
BnaA08.GH3-17.b     F.....LDGESCNIQEN.FKNKAPVVNYEDIKPFIQRISDGDSSDILSAQPITELLTSSG
BnaA09.GH3-17.c     F.....LDGESDKNQQS.FKNKVPVVNYDDIKPFIQRIADGESSDIVSSQPITELLTSSG
BnaC08.GH3-17.a     ............................................................
BnaCX.GH3-17.b      F.....LDGESDKNQQS.FKNKVPVVNYDDIKPFIQRIADGESSDIVSVQPITELLTSSG
BnaCX.GH3-17.c      ............................................................
BnaA05GH3-18.a      F.....LHGSNDK..EL.FKKNVPLANYEDVKPYIERVANGEPSDLISGGPVTRFFRSSG
BnaCX.GH3-18.a      F.....LEGSSDK..EL.FKKNVPVVSYEDVKPYIDRVANGEPSDIISGEPITTFNRSSG
BnaA06.GH3-19.a     F.....LQGSSDK..EL.FKKNVPVVSYEDVKPYIDRVANGEPSDIISGEPITAFFRSSG
BnaA09.GH3-19.b     F.....LEGSSDK..EL.FKKNVPVVSYEDVKPYIDRVANGEPSDIISGEPITTFNRSSG
consensus>70        f.....l.g..d...e..fk...pvv.yed..pyidri.nge.s.i....pi.....ssg
```

```
                    110       120       130       140                 150
AtGH3-1             TSAGERKLMPTIKEELDRRQLLYSLLMPVMNL.........YV..PGLDKGKGMYFLFVK
AtGH3-2             TSAGERKLMPTIEEDLDRRQLLYSLLMPVMNL.........YV..PGLDKGKGLYFLFVK
AtGH3-3             TSAGERKLMPTIDEDMDRRQLLYSLLMPVMNL.........YV..PGLDKGKALYFLFVK
AtGH3-4             TSAGERKLMPTIEEDINRRQLLGNLLMPVMNL.........YV..PGLDKGKGLYFLFVK
AtGH3-5             TSGGERKLMPTIEEELDRRSLLYSLLMPVMSQ.........FV..PGLENGKGMYFLFIK
AtGH3-6             TSGGERKLMPTIEEELDRRSLLYSLLMPVMDQ.........FV..PGLDKGKGMYFLFIK
AtGH3-7             TSGGAQKMMPWNEKYLDNLTFMYDLRMHIISN.........NV..KDVEKGKAMMFYFTK
AtGH3-8             TSGGKQKMFPRNNKYLENIKFIFYYRSLVISK.........HI..DGLEHGKGMVFNFCT
AtGH3-9             TSAGEPKLMPTIPEDLDRRTFLYNLIIPIVNK.........YI..TGLDKGKAMYLNFVK
AtGH3-10            TTEGRQKYVPFTRHSAQTTLQIFRLSAAYRSR.........FY..PIREGGRILEFIYAG
AtGH3-11            TSQGRPKFIPFTDELMENTLQLFRTAFAFRNR.........DF..PIDDNGKALQFIFSS
AtGH3-12            TSGGAQKMMPWNNKYLDNLTFIYDLRMQVITK.........HV..KGVEEGKGMMFLFTK
AtGH3-13            TTAGIQNLIPLTTEDGEQRIMFGSLYRSLLYK.........YV..EGIREGKSLTFYFVN
AtGH3-14            TSGGVPKLIPLTTEELEQRISFASLYRPLLYK.........YI..EGIRERKSFMLYFVT
AtGH3-15            TSGGVPKLIPLTTEDLEQRISFSSLYAPLLYK.........HI..DGLSEGKSLIFYFVT
AtGH3-16            TSGGIPKLIPLTAEELEQRILFGFLYVPLVFK.........HI..EGLTQGKSLMFYFVT
AtGH3-17            TSAGKPKLMPSTAEELERKTFFYSMLVPIMNK.........YV..DGLDEGKGMYLLFIK
AtGH3-18            TSGGKQKVFPVNDKYIEKLGYVIALRSLAMSK.........HF.DSGGEQGKAMEFHCTK
AtGH3-19            TSGGIHKIFPVNDKYIENLGYLLAVSSLITSN............DKVDEKGKKMAFLYNR
BraX.GH3-1.a        TSAGERKLMPTIQEELDRRQLLYSLLMPVMNL.........YV..PGLDKGKGLYFLFVK
BraX.GH3-2.a        TSAGERKLMPTIDEDLDRRQLLYSLLMPVMNL.........YV..PGLDKGKGLYFLFVK
BraA09.GH3-3.a      TSAGERKLMPTIEEDMDRRQLLYSLQMPVMNL.........YV..PGLGKGKALHFLFVK
BraA01.GH3-5.a      TSGGERKLMPTIEEELDRRSFLYSFLMPVMSQ.........FV..PGLDKGKGMYFLFIK
BraA03.GH3-5.b      TSGGERKLMPTIEEELDRRSLLYSFLMPVMSQ.........FV..PGLDKGKGMYFLFIK
BraA02.GH3-6.a      TSGGERKLMPTIEEELDRRSLLYSLLMPVMSQ.........FV..PGLDKGKGMYFLFIK
BraA03.GH3-6.b      TSGGERKLMPTIEEELDRRSLLYSLLMPVMSQ.........FV..PGLDKGKGMYFLFIK
BraA06.GH3-7.a      TSGGANKILPSNSKYLDGSAFSTDLIAHVIRK.........HV..KGVERGKGMLFFLTG
BraA08.GH3-7.b      TSGGAQKIMPWNEKFLDYLTFMYDLRMHVITN.........YV..KGLEKGKAMMFYFTK
BraA02.GH3-8.a      TSGGKRKMFPRNNKYLENLKFIYFYRSLVITK.........HI..DGLEHGKGMVFNFCT
BraA03.GH3-8.b      TSGGKRKMFPCNKKYLENLNFIYCYRSLVISK.........HF..DGLEHGKGMVFNFCV
BraA03.GH3-8.c      TSGGKQKLIPLNNKYLENARLLFDLRYLVLSK.........HV..DGHNEGKGLHLIFLK
BraA03.GH3-8.d      TSGGKQKLIPLNNKYVENARFLSDIRYLVLSK.........HI..EGHNKGKGLHLLFLK
BraA06.GH3-8.e      TSSGNPKIFPANNIFFKNVQIIYTLCSVVMSK.........HV..EGFKEGKVIWF...Q
BraA06.GH3-8.f      TSSGNQKIFPANNIFFKNIQIFYTLGSLVMSK.........CF..DGYKQGKVIRFTFTH
BraA09.GH3-8.g      TSGGKQKIFPVNNKFFEDMAFIYALRSFLISK.........HT..EGDEKGKVVMLFFAR
BraA09.GH3-8.h      TSSGEKKIFPAND.NFEDILLGLALSSLVMSK.........HV..DGYKQGKVMIFKFIQ
BraA10.GH3-8.i      TTAGKQKIFPVNNKFFEDMSFIIALRLFVISK.........HF..EGGQKGKALTLFFTR
BraA09.GH3-10.a     TTEGRQKYVPFTCHSSQTTLQIFRLSAAYRSR.........FY..PIKEGGRILEFIYAG
BraA03.GH3-11.a     TSQGRPKFIPFTDELMENTLQLFRTAFAFRNR.........DF..PIDDNGRALQFIFSS
BraA04.GH3-11.b     TSQGRPKFVPFTDELMENTIQLFRTAFVFRNR.........EF..PIDDNGRALQFIFSS
BraA05.GH3-11.c     TSQGRPKFVPFTDELMENTLQLFRTAFAFRNR.........EF..PIDDNGRALQFIFSS
BraA02.GH3-12.a     TSGGAQKMMPWNNKYLDNLTFAYDLRMHVITK.........HV..KGLEQGKGMMFLFTK
BraA03.GH3-12.b     TSGGAQKMMPWNHKYLDNLTFAYDLRMHVITK.........HV..KGLEEGKGMMFLFTK
BraA06.GH3-12.c     TSGGANKILPSNNKFLDGYAFSNDLLVHIISK.........HV..KGVERGKGMIFSLTG
BraA10.GH3-12.d     TSGGAQKMMPWNNKYLDNLTFAYDLRMHVITK.........HV..KGLEEGKGMMFLFTK
BraA10.GH3-14.a     TSGGVPKLIPLTAEDMEQRNLFVSLYRPLPFK.........HI..KGLSEGKSLLFYFLT
BraA03.GH3-15.a     TSGGVPKLIPMTAEELEQRMLFSSLHRPLISK.........QI..EGISEGKSLSFFFVT
BraA07.GH3-15.b     TSGGVPKLIPFTAEELEQRMLFMSLYRPILFK.........HI..EGRSEEKSLMFYFVT
BraA02.GH3-16.a     TSGGYPKLIPLTAEDMEQRILFASLYAPLVFK.........HI..GGLTEGKSLMFYFVT
BraA07.GH3-17.a     TSAGKPKLMPSTAKELDRKTFFYSMLVPVMNK.........YV..NGLDEGKGMYLLFIK
BraA09.GH3-17.b     TSAGKPKLMPSTAEELERKTIFYSMLVPVMNK.........YV..NGLDEGKGMYLLFIK
BraAX.GH3-17.c      TSGGKPKFMPSTAEELDRKTFFYNMLVPVMNK.........FV..NGLDEGKGMYLLFIK
BraA04.GH3-19.a     TSGGKQKIYALNN..................K.........HI..KGVEDGKVMTFLNTR
BraA06.GH3-19.b     TTSGNQKIFPANNITFENILFAFSLSSVVMSK.........HV..EVYKQGKVMSFTFTQ
BraA09.GH3-19.c     TSSGNQKIYPTNNIYFENLLFGYALSSVVMSK.........HV..DGYKQGKMMIFRFTH
BraA09.GH3-19.d     TSSGNQKIYPANNIFIENTLFGFGLSSIVMSK.........HV..DGYKQGKVMVFSFTH
BraAX.GH3-19.e      TSGGKQKIFPVNDEAIEQLGYVLALRSLVMSK.........HFSDNGVEQGKTMEFHYTK
BolC09.GH3-1.a      TSAGERKLMPTIQEELDRRQLLYSLLMPVMNL.........YV..PGLDKGKGLYFLFVK
BolC01.GH3-2.a      TSAGERKLMPTIDEDLDRRQLLYSLLMPVMNL.........YV..PGLDKGKGLYFLFVK
BolC08.GH3-3.a      TSAGERKLMPTIEEDMDRRQLLYSLQMPVMNL.........YV..PGLGKGKALHFLFVK
BolC01.GH3-5.a      TSGRERKLMPTIEEELDRRSFLYSFLMPVMSQ.........FV..PGLDKGKGMYFLFIK
BolC07.GH3-5.b      TSGGERKLMPTIEEELDRRSLLYSFLMPVMSQ.........FV..PGLDKGKGMYFLFVK
BolC02.GH3-6.a      TSGGERKLMPTIEEELDRRSLLYSLLMPVMSQ.........FV..PGLDKGKGMYFLFIK
BolC03.GH3-6.b      TSGGERKLMPTIEEELDRRSLLYSLLMPVMSQ.........FV..PGLDKGKGMYFLFIK
BolC06.GH3-7.a      TSGGANKLLPSNNKFLDGYAFVNDLLAHVISK.........HV..EGVEKGKGMVFLLTG
BolC01.GH3-8.a      TSSGKQKLIPMNDKYLENTKLLTDFRSIVLSK.........HV..DGHNQGKSLKLLFTG
BolC03.GH3-8.b      TSGGKRKMFPCNKKYLENLNFIYCYRSLVISK.........HF..DGLEHGKGMVFNVCV
BolC07.GH3-8.c      TSGGKQKLIPLNNKYLENARLLFDLRYLVLSK.........HV..DGHNEGKGLHLIFLK
BolC08.GH3-8.d      TSSGNPKIFPATD.NFENIKSAGSLNSLIMSK.........HV..DGYNQGKVMIFRFTH
BolC09.GH3-8.e      TTAGKQKIFPVNNKFFEDMSFIIALRLFVISK.........HF..EGGQKGKALTLFFTR
BolC09.GH3-10.a     TTEGRQKYVPFTRHSSQTTLQIFRLSAAYRSR.........FY..PIKEGGRILEFIYAG
BolC03.GH3-11.a     TSQGRPKFIPFTDELMENTLQLFRTAFAFRNR.........DF..PIDDNGRALQFIFSS
BolC04.GH3-11.b     TSQGRPKFIPFTDELMENTLQLFRTAFAFRNR.........EF..PIDDNGRALQFIFSS
BolCX.GH3-11.c      TSQGRPKFVPFTDELMENTLQLFRTAFAFRNR.........EF..PIDDNGRALQFIFSS
BolC03.GH3-12.a     TSGGAQKMMPWNHKYLDNLTFAYDLRMHVITK.........HV..KGLEEGKGMMFLFTK
BolC09.GH3-12.b     TSGGAQKMMPWNNKYLDNLTFAYDLRMHVITK.........HV..KGLEVGKGMMFLFTK
BolC09.GH3-14.a     TSGGVPKLIPLTAEDMEQRNLFVSLYRPLPFK.........HI..KGLSEGKSLLFYFLT
BolC03.GH3-15.a     TSGGVPKLIPMTAEELEQRMLFSSLHRPLISK.........QI..EGISEGKSLSFFFVT
BolCX.GH3-16.a      TSGGYPKLIPLTAEDMEQRILFASLYAPLVFK.........HI..GGLTEGKSLMFYFVT
BolC07.GH3-17.a     TSAGKPKLMPSTAKELDRKTFFYSMLVPVMNK.........YV..NGLDEGKGMYLLFIK
BolC08.GH3-17.b     TSGGKPKLMPSTAEELDRKTFFYNMLVPVMNK.........FV..NGLDEGKGMYLLFIK
BolC08.GH3-19.a     TSSGNQKIYPANNIYFENMRSGYALSSVVMSK.........HV..DGYKQGKMMIFRFTH
BnaA09R.GH3-1.a     TSAGERKLMPTIQEELDRRQLLYSLLMPVMNL.........YV..PGLDKGKGLYFLFVK
BnaCX.GH3-1.a       TSAGERKLMPTIQEELDRRQLLYSLLMPVMNL.........YV..PGLDKGKGLYFLFVK
BnaA01.GH3-2.a      TSAGERKLMPTIDEDLDRRQLLYSLLMPVMNL.........YV..PGLDKGKGLYFLFVK
BnaC01.GH3-2.a      TSAGERKLMPTIDEDLDRRQLLYSLLMPVMNL.........YV..PGLDKGKGLYFLFVK
BnaA09.GH3-3.a      TSAGERKLMPTIEEDMDRRQLLYSLQMPVMNL.........YV..PGLGKGKALHFLFVK
BnaC08.GH3-3.a      TSAGERKLMPTIEEDMDRRQLLYSLQMPVMNL.........YV..PGLGKGKALHFLFVK
BnaA01.GH3-5.a      TSGGERKLMPTIEEELDRRSFLYSFLMPVMSQ.........FV..PGLDKGKGMYFLFIK
BnaA03.GH3-5.b      TSGGQRKLMPTIEEELDRRSLLYSFLMPVMSQ.........FV..PGLDKGKGMYFLFIK
BnaC01.GH3-5.a      TSGGERKLMPTIEEELDRRSFLYSFLMPVMSQ.........FV..PGLDKGKGMYFLFIK
BnaC07.GH3-5.b      TSGGERKLMPTIEEELDRRSLLYSFLMPVMSQ.........FV..PGLDKGKGMYFLFVK
BnaA03.GH3-6.a      TSGGERKLMPTIEEELDRRSLLYSLLMPVMSQ.........FV..PGLDKGKGMYFLFIK
BnaAX.GH3-6.b       TSGGERKLMPTIEEELDRRSLLYSLLMPVMSQ.........FV..PGLDKGKGMYFLFIK
BnaC03.GH3-6.a      TSGGERKLMPTIEEELDRRSLLYSLLMPVMSQ.........FV..PGLDKGKGMYFLFIK
BnaCX.GH3-6.b       TSGGERKLMPTIEEELDRRSLLYSLLMPVMSQ.........FV..PGLDKGKGMYFLFIK
BnaA06.GH3-7.a      TSGGANKILPSNSKYLDGSAFSTDLIAHVIRK.........HV..KGVERGKGMLFFLTG
BnaA08.GH3-7.b      TSGGAQKIMPWNEKFLDYLTFMYDLRMHVITN.........YV..KGLEKGKAMMFYFTK
BnaC06.GH3-7.a      TSGGANKLLPSNNKFLDGYAFVNYLLAHVISK.........HV..EGVEKGKGMVFLLTG
BnaA03.GH3-8.a      TSGGKRKMFPCNKKYLENLNFIYCYRSLVISK.........HF..DGLEHGKGMVFNFCV
BnaA03.GH3-8.b      TSGGKQKLIPLNNKYLENARLLFDLRYLVLSK.........HV..DGHNEGKGLHLIFLK
BnaA03.GH3-8.c      TSGGKQKLIPLNNKYVENARFLSDIRYLVLSK.........HI..EGHNKGKGLHLLFLK
BnaA06.GH3-8.d      TSSGNQKIFPANNIFFKNIHIISTLSSLVISK.........HV..DGFKEGKVIRFAFIH
```

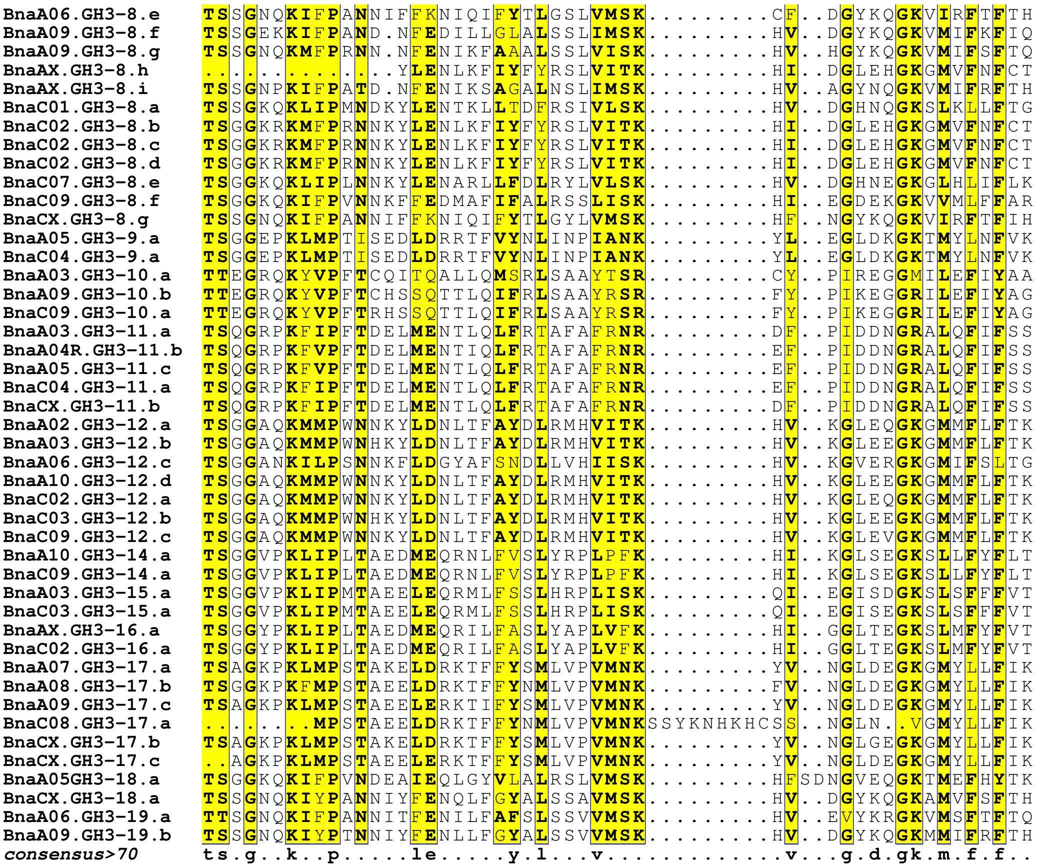
BnaA06.GH3-8.e TSSGNQKIFPANNIFFKNIQIFYTLGSLVMSK.........CF..DGYKQGKVIRFTFTH
BnaA09.GH3-8.f TSSGEKKIFPAND.NFEDILLGLALSSLIMSK.........HV..DGYKQGKVMIFKFIQ
BnaA09.GH3-8.g TSSGNQKMFPRNN.NFENIKFAAALSSLVISK.........HV..HGYKQGKVMIFSFTQ
BnaAX.GH3-8.h ..............YLENLKFIYFYRSLVITK.........HI..DGLEHGKGMVFNFCT
BnaAX.GH3-8.i TSSGNPKIFPATD.NFENIKSAGALNSLIMSK.........HV..AGYNQGKVMIFRFTH
BnaC01.GH3-8.a TSSGKQKLIPMNDKYLENTKLLTDFRSIVLSK.........HV..DGHNQGKSLKLLFTG
BnaC02.GH3-8.b TSGGKRKMFPRNNKYLENLKFIYFYRSLVITK.........HI..DGLEHGKGMVFNFCT
BnaC02.GH3-8.c TSGGKRKMFPRNNKYLENLKFIYFYRSLVITK.........HI..DGLEHGKGMVFNFCT
BnaC02.GH3-8.d TSGGKRKMFPRNNKYLENLKFIYFYRSLVITK.........HI..DGLEHGKGMVFNFCT
BnaC07.GH3-8.e TSGGKQKLIPLNNKYLENARLLFDLRYLVLSK.........HV..DGHNEGKGLHLIFLK
BnaC09.GH3-8.f TSGGKQKIFPVNNKFFEDMAFIFALRSSLISK.........HI..EGDEKGKVVMLFFAR
BnaCX.GH3-8.g TSSGNQKIFPANNIFFKNIQIFYTLGYLVMSK.........HF..NGYKQGKVIRFTFIH
BnaA05.GH3-9.a TSGGEPKLMPTISEDLDRRTFVYNLINPIANK.........YL..EGLDKGKTMYLNFVK
BnaC04.GH3-9.a TSGGEPKLMPTISEDLDRRTFVYNLINPIANK.........YL..EGLDKGKTMYLNFVK
BnaA03.GH3-10.a TTEGRQKYVPFTCQITQALLQMSRLSAAYTSR.........CY..PIREGGMILEFIYAA
BnaA09.GH3-10.b TTEGRQKYVPFTCHSSQTTLQIFRLSAAYRSR.........FY..PIKEGGRILEFIYAG
BnaC09.GH3-10.a TTEGRQKYVPFTRHSSQTTLQIFRLSAAYRSR.........FY..PIKEGGRILEFIYAG
BnaA03.GH3-11.a TSQGRPKFIPFTDELMENTLQLFRTAFAFRNR.........DF..PIDDNGRALQFIFSS
BnaA04R.GH3-11.b TSQGRPKFVPFTDELMENTIQLFRTAFAFRNR.........EF..PIDDNGRALQFIFSS
BnaA05.GH3-11.c TSQGRPKFVPFTDELMENTLQLFRTAFAFRNR.........EF..PIDDNGRALQFIFSS
BnaC04.GH3-11.a TSQGRPKFIPFTDELMENTLQLFRTAFAFRNR.........EF..PIDDNGRALQFIFSS
BnaCX.GH3-11.b TSQGRPKFIPFTDELMENTLQLFRTAFAFRNR.........DF..PIDDNGRALQFIFSS
BnaA02.GH3-12.a TSGGAQKMMPWNNKYLDNLTFAYDLRMHVITK.........HV..KGLEQGKGMMFLFTK
BnaA03.GH3-12.b TSGGAQKMMPWNHKYLDNLTFAYDLRMHVITK.........HV..KGLEEGKGMMFLFTK
BnaA06.GH3-12.c TSGGANKILPSNNKFLDGYAFSNDLLVHIISK.........HV..KGVERGKGMIFSLTG
BnaA10.GH3-12.d TSGGAQKMMPWNNKYLDNLTFAYDLRMHVITK.........HV..KGLEEGKGMMFLFTK
BnaC02.GH3-12.a TSGGAQKMMPWNNKYLDNLTFAYDLRMHVITK.........HV..KGLEQGKGMMFLFTK
BnaC03.GH3-12.b TSGGAQKMMPWNHKYLDNLTFAYDLRMHVITK.........HV..KGLEEGKGMMFLFTK
BnaC09.GH3-12.c TSGGAQKMMPWNNKYLDNLTFAYDLRMHVITK.........HV..KGLEVGKGMMFLFTK
BnaA10.GH3-14.a TSGGVPKLIPLTAEDMEQRNLFVSLYRPLPFK.........HI..KGLSEGKSLLFYFLT
BnaC09.GH3-14.a TSGGVPKLIPLTAEDMEQRNLFVSLYRPLPFK.........HI..KGLSEGKSLLFYFLT
BnaA03.GH3-15.a TSGGVPKLIPMTAEELEQRMLFSSLHRPLISK.........QI..EGISDGKSLSFFFVT
BnaC03.GH3-15.a TSGGVPKLIPMTAEELEQRMLFSSLHRPLISK.........QI..EGISEGKSLSFFFVT
BnaAX.GH3-16.a TSGGYPKLIPLTAEDMEQRILFASLYAPLVFK.........HI..GGLTEGKSLMFYFVT
BnaC02.GH3-16.a TSGGYPKLIPLTAEDMEQRILFASLYAPLVFK.........HI..GGLTEGKSLMFYFVT
BnaA07.GH3-17.a TSAGKPKLMPSTAKELDRKTFFYSMLVPVMNK.........YV..NGLDEGKGMYLLFIK
BnaA08.GH3-17.b TSGGKPKFMPSTAEELDRKTFFYNMLVPVMNK.........FV..NGLDEGKGMYLLFIK
BnaA09.GH3-17.c TSAGKPKLMPSTAEELERKTIFYSMLVPVMNK.........YV..NGLDEGKGMYLLFIK
BnaC08.GH3-17.a .........MPSTAEELDRKTFFYNMLVPVMNKSSYKNHKHCSS..NGLN..VGMYLLFIK
BnaCX.GH3-17.b TSAGKPKLMPSTAKELDRKTFFYSMLVPVMNK.........YV..NGLGEGKGMYLLFIK
BnaCX.GH3-17.c ..AGKPKLMPSTAEELERKTFFYSMLVPVMNK.........YV..NGLDEGKGMYLLFIK
BnaA05GH3-18.a TSGGKQKIFPVNDEAIEQLGYVLALRSLVMSK.........HFSDNGVEQGKTMEFHYTK
BnaCX.GH3-18.a TSSGNQKIYPANNIYFENQLFGYALSSAVMSK.........HV..DGYKQGKAMVFSFTH
BnaA06.GH3-19.a TTSGNQKIFPANNITFENILFAFSLSSVVMSK.........HV..EVYKRGKVMSFTFTQ
BnaA09.GH3-19.b TSSGNQKIYPTNNIYFENLLFGYALSSVVMSK.........HV..DGYKQGKMMIFRFTH
consensus>70 ts.g..k..p.....le.....y.l...v.............v...g.d.gk.m.f.f..

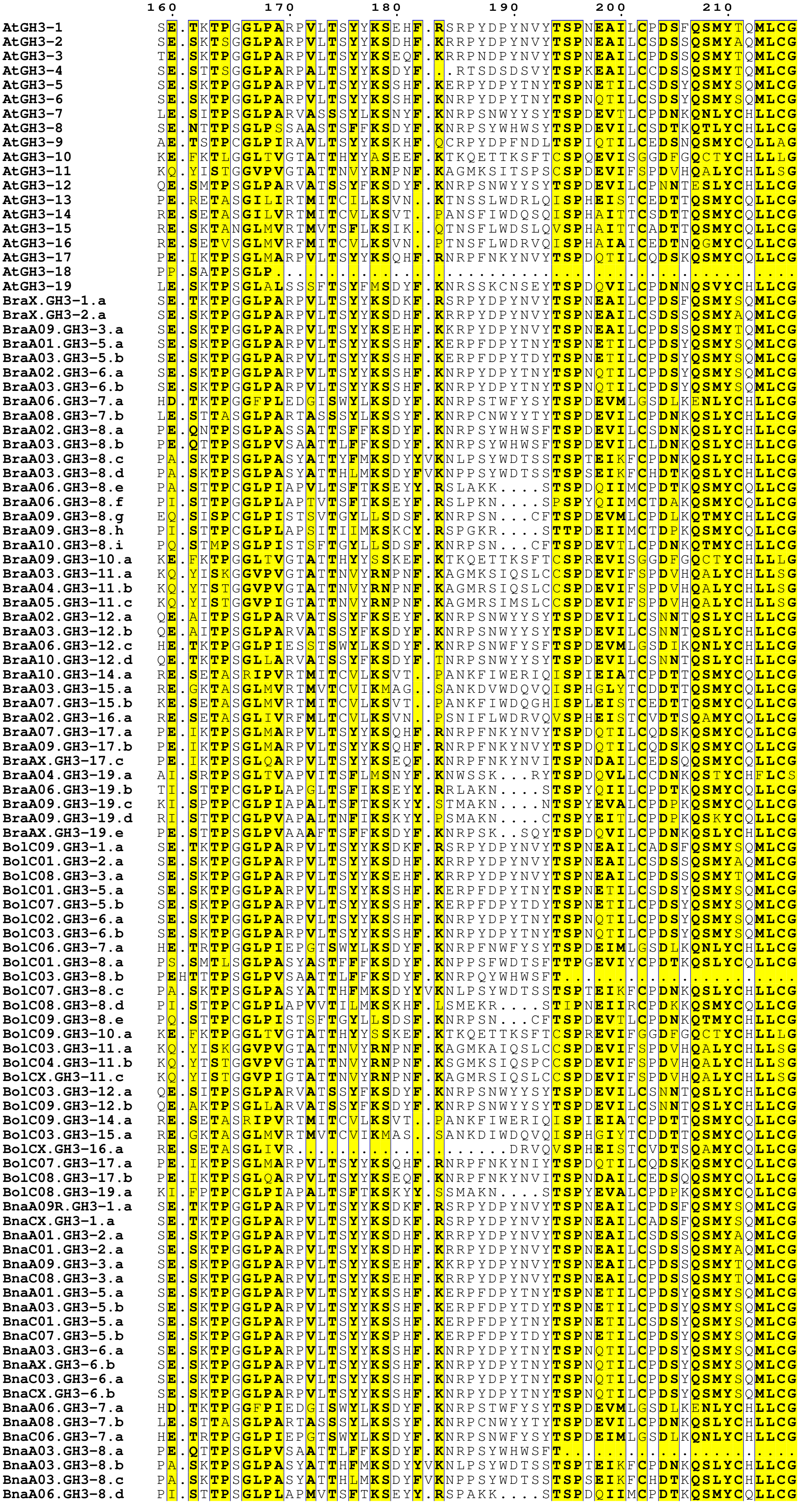
160
170
180
190
200
210
AtGH3-1
AtGH3-2
AtGH3-3
AtGH3-4
AtGH3-5
AtGH3-6
AtGH3-7
AtGH3-8
AtGH3-9
AtGH3-10
AtGH3-11
AtGH3-12
AtGH3-13
AtGH3-14
AtGH3-15
AtGH3-16
AtGH3-17
AtGH3-18
AtGH3-19
BraX.GH3-1.a
BraX.GH3-2.a
BraA09.GH3-3.a
BraA01.GH3-5.a
BraA03.GH3-5.b
BraA02.GH3-6.a
BraA03.GH3-6.b
BraA06.GH3-7.a
BraA08.GH3-7.b
BraA02.GH3-8.a
BraA03.GH3-8.b
BraA03.GH3-8.c
BraA03.GH3-8.d
BraA06.GH3-8.e
BraA06.GH3-8.f
BraA09.GH3-8.g
BraA09.GH3-8.h
BraA10.GH3-8.i
BraA09.GH3-10.a
BraA03.GH3-11.a
BraA04.GH3-11.b
BraA05.GH3-11.c
BraA02.GH3-12.a
BraA03.GH3-12.b
BraA06.GH3-12.c
BraA10.GH3-12.d
BraA10.GH3-14.a
BraA03.GH3-15.a
BraA07.GH3-15.b
BraA02.GH3-16.a
BraA07.GH3-17.a
BraA09.GH3-17.b
BraAX.GH3-17.c
BraA04.GH3-19.a
BraA06.GH3-19.b
BraA09.GH3-19.c
BraA09.GH3-19.d
BraAX.GH3-19.e
BolC09.GH3-1.a
BolC01.GH3-2.a
BolC08.GH3-3.a
BolC01.GH3-5.a
BolC07.GH3-5.b
BolC02.GH3-6.a
BolC03.GH3-6.b
BolC06.GH3-7.a
BolC01.GH3-8.a
BolC03.GH3-8.b
BolC07.GH3-8.c
BolC08.GH3-8.d
BolC09.GH3-8.e
BolC09.GH3-10.a
BolC03.GH3-11.a
BolC04.GH3-11.b
BolCX.GH3-11.c
BolC03.GH3-12.a
BolC09.GH3-12.b
BolC09.GH3-14.a
BolC03.GH3-15.a
BolCX.GH3-16.a
BolC07.GH3-17.a
BolC08.GH3-17.b
BolC08.GH3-19.a
BnaA09R.GH3-1.a
BnaCX.GH3-1.a
BnaA01.GH3-2.a
BnaC01.GH3-2.a
BnaA09.GH3-3.a
BnaC08.GH3-3.a
BnaA01.GH3-5.a
BnaA03.GH3-5.b
BnaC01.GH3-5.a
BnaC07.GH3-5.b
BnaA03.GH3-6.a
BnaAX.GH3-6.b
BnaC03.GH3-6.a
BnaCX.GH3-6.b
BnaA06.GH3-7.a
BnaA08.GH3-7.b
BnaC06.GH3-7.a
BnaA03.GH3-8.a
BnaA03.GH3-8.b
BnaA03.GH3-8.c
BnaA06.GH3-8.d

```
BnaA06.GH3-8.e      PI.STTPGGLPLAPTVTSFTKSEYF.RSLPKN....SPSPYQIIMCTDAKQSMYCQLLCG
BnaA09.GH3-8.f      PI.STTPCGLPLAPSITIIMKSKNY.RSPGKR....STTPDEIIMCTDPKQSMYCQLLCG
BnaA09.GH3-8.g      PL.STTPCGLPLAPATTILLKSKNY.RSPAKN....STSPDEIILCTDAKQSMYCQLLCG
BnaAX.GH3-8.h       PE.QNTPSGLPASSATTSFFKSDYF.KNRPSYWHWSFTSPDEVILCSDNKQSLYCHLLCG
BnaAX.GH3-8.i       PI.STTPCGLPLASVVTILMKSKHF.LSMEKR....STIPKEIIRCPDKKQSMYCQLLFG
BnaC01.GH3-8.a      PS.SMTLSGLPASYASTFFFKSDYF.KNPPSFWDTSFTTPGEVIYCPDTKQSLYCQLLCG
BnaC02.GH3-8.b      PE.QNTPSGLPASSATTSFFKSDYF.KNRPSYWHWSFTSPDEVILCSDNKQSLYCHLLCG
BnaC02.GH3-8.c      PE.QNTPSGLPASSATTSFFKSDYF.KNRPSYWHWSFTSPDEVILCSDNKQSLYCHLLCG
BnaC02.GH3-8.d      PE.QNTPSGLPASSATTSFFKSDYF.KNRPSYWHWSFTSPDEVILCSDNKQSLYCHLLCG
BnaC07.GH3-8.e      PA.SKTPSGLPASYATTHFMKSDYYVKNLPSYWDTSSTSPTEIKFCPDNKQSLYCHLLCG
BnaC09.GH3-8.f      EQ.SITPCGLPISTSVTGYLLSDSF.KNRPSN...CFTSPDEVTLCPDVKQTMYCHLLCG
BnaCX.GH3-8.g       PT.STTPCGLPLAPTVTSFTKSEFF.RSLPKN....SPSPYQIILCPDAKQSMYCQLLCG
BnaA05.GH3-9.a      AE.TSTPCGLPIRTVLTSYYKSKHF.QCRPYDPFNDLTSPIQTILCEDSNQSMYCQLLAA
BnaC04.GH3-9.a      AE.TSTPCGLPIRTVLTSYYKSKHF.QCRPYDPFNDLTSPIQTILCEDSNQSMYCQLLAA
BnaA03.GH3-10.a     KV.FKTPGGLDVGTATTLYYASKEF.KTKQEATKSFTCSPQEVISGGDFVQCTYCHLLLG
BnaA09.GH3-10.b     KE.FKTPGGLTVGTATTHYYSSKEF.KTKQETTKSFTCSPREVISGGDFGQCTYCHLLLG
BnaC09.GH3-10.a     KE.FKTPGGLTVGTATTHYYSSKEF.KTKQETTKSFTCSPREVISGGDFGQCTYCHLLLG
BnaA03.GH3-11.a     KQ.YISKGGVPVGTATTNVYRNPNF.KAGMKSIQSLCCSPDEVIFSPDVHQALYCHLLSG
BnaA04R.GH3-11.b    KQ.YTSTGGVPVGTATTNVYRNPNF.KSGMRSIQSLCCSPDEVIFSPDVHQALYCHLLSG
BnaA05.GH3-11.c     KQ.YISTGGVPIGTATTNVYRNPNF.KAGMRSIMSLCCSPDEVIFSPDVHQALYCHLLSG
BnaC04.GH3-11.a     KQ.YTSTGGVPVGTATTNVYRNPNF.KSGMKSIQSPCCSPDEVIFSPDVHQALYCHLLSG
BnaCX.GH3-11.b      KQ.YISKGGVPVGTATTNVYRNPNF.KAGMKAIQSLCCSPDEVIFSPEVHQALYCHLLSG
BnaA02.GH3-12.a     QE.AITPSGLPARVATSSYFKSEYF.KNRPSNWYYSYTSPDEVILCSNNTQSLYCHLLCG
BnaA03.GH3-12.b     QE.AITPSGLPARVATSSYFKSDYF.KNRPSNWYYSYTSPDEVILCSNNTQSLYCHLLCG
BnaA06.GH3-12.c     HE.TKTPGGLPIESSTSWYLKSDYF.KNRPSNWFYSFTSPDEVMLGSDIKQNLYCHLLCG
BnaA10.GH3-12.d     QE.TKTPSGLLARVATSSYFKSDYF.TNRPSNWYYSYTSPDEVILCSNNTQSLYCHLLCG
BnaC02.GH3-12.a     QE.AITPSGLPARVATSSYFKSDYF.KNRPSNWYYSYTSPDEVILCSNNTQSLYCHLLCG
BnaC03.GH3-12.b     QE.SITPSGLPARVATSSYFKSDYF.KNRPSNWYYSYTSPDEVILCSNNTQSLYCHLLCG
BnaC09.GH3-12.c     QE.AKTPSGLLARVATSSYFKSDYF.KNRPSNWYYSYTSPDEVILCSNNTQSLYCHLLCG
BnaA10.GH3-14.a     RE.SETASRIPVRTMITCVLKSVT..PANKFIWERIQISPIEIATCPDTIQSMYCQLLCG
BnaC09.GH3-14.a     RE.SETASRIPVRTMITCVLKSVT..PANKFIWERIQISPIEIATCPDTTQSMYCQLLCG
BnaA03.GH3-15.a     RE.GKTASGLMVRTMVTCVIKMAG..SANKDIWDQVQISPHGLYTCDDTTQSMYCQLLCG
BnaC03.GH3-15.a     RE.GKTASGLMVRTMVTCVIKMAG..SANKDIWDQVQISPHGIYTCDDTTQSMYCQLLCG
BnaAX.GH3-16.a      RE.SETASGLIVRFMLTCVLKSVN..PSNIFLWDRVQVSPHEISTCVDTSQAMYCQLLCG
BnaC02.GH3-16.a     RE.SETASGLIVRFMLTCVLKSVN..PSNIFLWDRVQVSPHEISTCVDTSQAMYCQLLCG
BnaA07.GH3-17.a     PE.IKTPSGLMARPVLTSYYKSQHF.RNRPFNKYNVYTSPDQTILCQDSKQSMYCQLLCG
BnaA08.GH3-17.b     PE.IKTPAGLQARPVLTSYYKSEQF.KNRPFNKYNVITSPNDAILCEDSQQSMYCQLLCG
BnaA09.GH3-17.c     PE.IKTPSGLMARPVLTSYYKSQHF.RNRPFNKYNVYTSPDQTILCQDSKQSMYCQLLCG
BnaC08.GH3-17.a     PE.IKTPSGLQARPVLTSYYKSEQF.KNRPFNKYNVITSPNDAILCEDSQQSMYCQLLCG
BnaCX.GH3-17.b      PE.IKTPSGLMARPVLTSYYKSQHF.RNRPFNKYNIYTSPDQTILCQDSKQSMYCQLLCG
BnaCX.GH3-17.c      PE.IKTPSGLMARPVLTSYYKSQHF.RNRPFNKYNVYTSPDQTILCQDSKQSMYCQLLCG
BnaA05GH3-18.a      PE.STTPSGLPVAAAFT...........................................
BnaCX.GH3-18.a      RI.STTLCGLP.................................................
BnaA06.GH3-19.a     TI.STTPCGLPLAPGLTSFIKSEYY.RRLAKN....STSPYQIILCPDTKQSMYCQLLCG
BnaA09.GH3-19.b     KI.SPTPCGLPIAPALTSFTKSKYY.STMAKN....NTSPYEVALCPDPKQSMYCQLLCG
consensus>70        .e...tp.glp.....t.y.ks..f............tsp.e.i.c.d..qsmyc.llcg
```

```
                    220       230       240       250       260       270
AtGH3-1             LLDRLSVLRVGAVFASGLLRAIRFLQLHWSRFAHDIELGCLDSEITDPSIRQCMSGIL.K
AtGH3-2             LLMRHEVLRLGAVFASGLLRAISFLQNNWKELARDISTGTLSSRIFDPAIKNRMSKILTK
AtGH3-3             LLMRHEVLRLGAVFASGLLRAIGFLQTNWKELADDISTGTLSSRISDPAIKESMSKILTK
AtGH3-4             LLMRHEVNRLGAVFPSGLLRAISFLQNNWKELSQDISTGTLSSKIFDHAIKTRMSNILNK
AtGH3-5             LCQHQEVLRVGAVFASGFIRAIKFLEKHWIELVRDIRTGTLSSLITDPSVREAVAKIL.K
AtGH3-6             LCQHKEVLRVGAVFASGFIRAIKFLEKHWPELARDIRTGTLSSEITDSSVREAVGEIL.K
AtGH3-7             LVQRNEVTRMGSIFASVMVRAIKFLEDSWEELCSNIRSGQLSEWITDIGCRDSVSLVLGG
AtGH3-8             LVQRDDVVKVGAAFVTILVRAINLLENSWKEICTNIRFGHLSEWITDISCRDSVSKILGE
AtGH3-9             LIHRHKVMRLGAVFASAFLRAISYLEKKWSQLCEDIRTGSLNPMITDPGCQMAMSCLLMS
AtGH3-10            LHYSSQVEFVASAFSYTIVQAFSFFEEIWREICADIKEGNLSSRITLPKMRKAVLALIR.
AtGH3-11            ILFRDQVQYVFAVFAHGLVHAFRTFEQVWEEIVTDIKDGVLSNRITVPSVRTAMSKLLT.
AtGH3-12            LVQRDEVVRTGSIFASVMVRAIEVLKNSWEELCSNIRSGHLSNWVTDLGCQNSVSLVLGG
AtGH3-13            LLQRDNVARLGAPFASVFIRVIKYLEGHWQELCSNIRTGRLSDWITDPQCVSGISKFLTA
AtGH3-14            LLQRDNVGRLGAPFASSFLKIIKFLEDHWPEFCSNIRTGCLSDWITDPQCVSGIGKFLTA
AtGH3-15            LLERDNVARLGAPFASSFLKVIKFLEDHWPELCSNIRTGRLSDWITDATCTSGIGKFLTA
AtGH3-16            LLQREHVARLGAPYASSFLKVIKFLEDHWPELCSNIRTGRLSDWITDAQCVSGIGNFLTA
AtGH3-17            LVQRSHVLRVGAVFASAFLRAVKFLEDHYKELCADIRTGTVTSWITDSSCRDSVLSILNG
AtGH3-18            ...RDEVVRVGAAFAFVLVRAIDFLEKHWKELCSNIRSGHVSEWITDLEGRNAVSTILRG
AtGH3-19            LSQREKVVGVSATFAHALIKAINALQIYWKELSSNIRSGHVSEWITDLDCKNAVSAILGG
BraX.GH3-1.a        LLDRLSVLRVGAVFASGLLRAIRFLQLHWSRLAYDIETGSLDSEITDPSIRQCMSDIL.K
BraX.GH3-2.a        LLMRHDVLRLGAVFASGLLRAISFLQNNWKELARDISTGTLSSRISDHAIKNRMSKILTK
BraA09.GH3-3.a      LLMRHEVLRLGAVFASGLLRAIRFLQTNWHELVNDISTGTLSSRISDPGIRESMSKILTK
BraA01.GH3-5.a      LCQHHEVLRVGAVFASGFIRAIKFLEKRWTELVRDIRTGTLSSSITDPSVREAVAKIL.K
BraA03.GH3-5.b      LCQHQEVLRVGAVFASGFIRAIKFLEKHWTELVRDIRTGTLSSLITDPSVREAVAKIL.K
BraA02.GH3-6.a      LCQHKEVLRVGAVFASGFIRAIKFLEKHWPELTRDIRTGTLSSEITDLSVREAVGEIL.K
BraA03.GH3-6.b      LCQHSEVLRVGAVFASGFIRAIKFLEKHWPQLVHDIRTGTLNSEITDLSVREAVEEIL.K
BraA06.GH3-7.a      LVQRDEVTRIGSTFASGMVRVIKVLEDSWKELCSNIRSGNLSEWITDSGCRNSVSLVLGG
BraA08.GH3-7.b      LVQRNEVTRMGSIFASVMVRAIKFLEDSWEELCSNIRSGQLSEWITDPGCRDSVSMVLGG
BraA02.GH3-8.a      IVQRDEVVKVGAAFVSILVRAITFLEKFWKEICTNIRCGHLSEWITDISCRDSVSKILGE
BraA03.GH3-8.b      IVLRDEVVKVGTAFVSTLLRVITFLEKHWKEICTNIRYGHLSEWITDISCRDSVSKILGE
BraA03.GH3-8.c      LVLRDEVTRVSANFASILVQGITFLENFWKEMCSNIRSGQLSDWITD.SCKGSVSMILGG
BraA03.GH3-8.d      LVMRDEVTRVSSGFASVLVQGITFLEKFWKEMCSNIRSGQLSDWITNLSCRDSVSKVLGG
BraA06.GH3-8.e      LVQRDDVVSVGAVFASVLVGVIHFLENYWKELASNIRSGHLSEWITDLGCRDYVSILLGD
BraA06.GH3-8.f      LVQRDEVASVGAVFAAVLVQVIHFLENYWKELASNIRFGHVSEWITDLSCRDSVSTVLVE
BraA09.GH3-8.g      LRQRDEVVAVAASFASSLVGAITFLESYWKEICNNIRSGHVSEWITDLSCRDAVTNILGG
BraA09.GH3-8.h      LVQRDEVVSVGALFASVLVQIIRFLEKYWKELCSNIRSGHVSDWITDHCCRDSVSTILGE
BraA10.GH3-8.i      LRLRDEVVNVTATFASSLVGAITFLESYWKEMCSNIRYGHVSEWITDLSCRDAVNNILGG
BraA09.GH3-10.a     LHFSKQVEFVASAFSYTIVQAFSLFEDTWRDICADIKEGSLSSRITLPRMRKAVLALIR.
BraA03.GH3-11.a     ILFRDQVQYVFASFAHGLVHAFRTFEQLWEEIVTDIEDGVLSSRITVPAVRTAMTKLLRG
BraA04.GH3-11.b     ILFRDQVQYVFASFAHGLVHAFRTFEQVWEEIVADIKEGVLTSRITVPSVRSAMSKLLR.
BraA05.GH3-11.c     ILFRDQVQYVFAPFAHGLVHAFRTFEHVWEEIVTDIKHGVLSSRITVPSVRSAMSKPITA
BraA02.GH3-12.a     LVQRDEVVRMGSIFASVMVRAIKFLETYWEELCSNIRSGHLSEWITDLGCRSSVSLVLGG
BraA03.GH3-12.b     LLQRDEVVRMGSIFASVMVRAIKFLETYWEELCSNIRSGHLSEWITDRGCRSSVSLVLEG
BraA06.GH3-12.c     LVQRDEVMRIGSTFASGMVRVIKVLQDSWKELCSNIRSGHLSEWITDSGCRNAVSMVTGG
BraA10.GH3-12.d     LLQRDEVVRMGSIFASVMVRAIKFLETYWEELCSNIRSGRLSEWITDLGCRSSVSLVLGG
BraA10.GH3-14.a     LIQRGNVSRLGAPFASSFLKVINFLGDHWSELCSNIKTGHVSPWIKDAQCLSGISKFLSA
BraA03.GH3-15.a     LLQRENVSRLGAPFASSFLKVIKFLEDHWKELCSNITTGRVSDWITDPQCLSGVGKFLGA
BraA07.GH3-15.b     LLERGNVSRLGAPFASTFLKVIRFLENHWSELCSNIRTGRVSDWITDANCVSGIGKFLEA
BraA02.GH3-16.a     LVQRENVARLGAPFASSFLKVINFLEDHWTALCSNIRTGRVSDWITDAQCVSGVSKFLTA
BraA07.GH3-17.a     LVQRSHVLRVGAVFASAFLRAVKFLEDHYKELCADIRTGTITSWITDSACRDSVLSILDG
BraA09.GH3-17.b     LVQRSHVLRVGAVFASAFLRAVKFLEDHYKELCADIRTGTVTSWITEPACRDSVLSVLQG
BraAX.GH3-17.c      LVQRSDVLRLGAIFASAFLRAVKFLENHFTELCADIRTGTVASWITDSGCRDSVLSILNG
BraA04.GH3-19.a     LVQREEVVSIFVPFACSLIDAIKFLETHWKELCNDIRSGHVSEWITDLGCRDSVSNILGV
BraA06.GH3-19.b     LVQRDEVVSVGSVFASVLVQVIHFLENYWKELASNIRSGHLSEWITDLSCRESVSTILVE
BraA09.GH3-19.c     LVHRDEVVSVGTTFPSVLVQIVHFLENYWKELSSNIRSGHVSDWITDLSCRDSVSIILGE
BraA09.GH3-19.d     LVQRDEVVGVGATFPSVLVRVINFLEKYWKELASNIRSGTVSDWITDRSCRDSVSTILGE
BraAX.GH3-19.e      LSQRDEVVRFGATFAHALVRAIDFLESNWKELCGNIRLGHVSEWITDLGCRSSVSAIIGG
BolC09.GH3-1.a      LLDRLSVLRVGAVFASGLLRAIRFLQLHWSRLANDIESGSLDSEITDPSIRQCMSDIL.K
BolC01.GH3-2.a      LVMRHDVLRLGAVFASGLLRAISFLQNNWKELARDISTGTLSSRISDHAIKTRMSKILTK
BolC08.GH3-3.a      LLMRHEILRLGAVFASGLLRAIRFLQTNWQELVSDISTGTLSSRISDPGIRESMSKILTE
BolC01.GH3-5.a      LCQHHEVLRVGAVFASGFIRAIKFLEKHWTELVRDIRTGTLSSSITDPSVREAVAKIL.K
BolC07.GH3-5.b      LCQHQEVLRVGAVFASGFIRAIKFLEKHWTELVRDIRTGTLSSLITDPSVREAVAKIL.K
BolC02.GH3-6.a      LCQHKEVLRVGAVFASGFIRAIKFLEKHWPELTRDIRTGTLSSEITDLSVREAVGEIL.K
BolC03.GH3-6.b      LCQHNEVLRVGAVFASGFIRAIKFLEKHWPELVHDIRTGTLNSEITDLSVREAVEEIL.K
BolC06.GH3-7.a      LVQRDEVVRISSTFASGMVRVIKVLEDSWKELCLNIRSGYLSEWITDSGCRNAVSMVLGG
BolC01.GH3-8.a      LVMRDEVTGIGAVFASIFVQGMIFLEKTWKDMCSNIRSGQLSDWITDLGCRESVSKILGG
BolC03.GH3-8.b      .IQRDEVVKVGTSFVSTLVRVITFLEKHWKEICTNTRYGHLSEWITD.SCRDSVSKILGE
BolC07.GH3-8.c      LVLRDEVTRVSANFASILVQGITFLENFWKEMCSNIRSGQLSYWITD.NCKDSVSMILGG
BolC08.GH3-8.d      LVQRDEVVSVGASFPSVLVQVINFLEKYWKELARNIRSGHLSEWITDLSCRDSVSIILGE
BolC09.GH3-8.e      LRLRDEVVNVTATFASSLVGAITFLESYWKEMCSNIRNGHVSEWITDLSCRDAVTNILGG
BolC09.GH3-10.a     LHFSKQVEFVASAFSYTIVQAFSLFEDTWRDICADIKEGSLSSRITLPKMRKAVLALIR.
BolC03.GH3-11.a     ILFRDQVQYVFASFAHGLVHAFRTFEQLWEEVVTDIEDGVLSSRITVPSVRTAMAKLLSG
BolC04.GH3-11.b     ILFRDQVQYVFASFAHGLVHAFRTFEQVWEEIVTDIKEGVLTSRITVPSVRSAMSKLLR.
BolCX.GH3-11.c      ILFRDQVQYVFAPFAHGLVHAFRTFEHVWEEIVTDIKHGVLSSRITVPSVRTAMSKLLTA
BolC03.GH3-12.a     LVQRDEVVRMGSIFASVMVRAIKFLETYWEELCSNIRSGHLSEWITDHGCRSSVSLVLGG
BolC09.GH3-12.b     LLQRDEVVRMGSIFASVMVRAIKFLETYWEELCSNIRTGRLSEWITDLGCRSSVSLVLGG
BolC09.GH3-14.a     LIQRGNVSRLGAPFASSFLKVINFLGDHWSELCSNIRTGRVSPWIKDAQCLSGISTFLSA
BolC03.GH3-15.a     LLQRESVSRLGAPFASSFLKVIKFLEDHWKELCSNIRTGRVSDWITDPQCLSGVGKFLTA
BolCX.GH3-16.a      LVQRENVARLGAPFASSFLKVINFLEDHWTELCSNIRTGRVSDWITDAQCVSGVSKFLTA
BolC07.GH3-17.a     LVQRSHVLRVGAVFASAFLRAVKFLEDHYKELCADIRTGTITSWITDSACRDSVLSILEG
BolC08.GH3-17.b     LVQRSHVLRLGAIFASAFLRAVKFLENHFTELCADIRTGTVASWITDSGCRDSVLSILNG
BolC08.GH3-19.a     LVQRDEVVSVGTTFPSVLVQIVHFLENYWKELSSNIRSGHVSDWITDLSCRDSVSVILGE
BnaA09R.GH3-1.a     LLDRLSVLRVGAVFASGLLRAIRFLQLHWSRLANDIESGSLDSEITDPSIRQCMSDIL.K
BnaCX.GH3-1.a       LLDRLSVLRVGAVFASGLLRAIRFLQLHWSRLANDIESGSLDSEITDPSIRQCMSVIL.K
BnaA01.GH3-2.a      LLMRHDVLRLGAVFASGLLRAISFLQNNWKELARDISTGTLSSRISDHAIKNRMSKILTK
BnaC01.GH3-2.a      LVMRHDVLRLGAVFASGLLRAISFLQNNWKELARDISTGTLNSRISDHAIKTRMSKILTK
BnaA09.GH3-3.a      LLMRHEVLRLGAVFASGLLRAIRFLQTNWHELVNDISTGTLSSRISDPGIRESMSKILTK
BnaC08.GH3-3.a      LLMRHEVLRLGAVFASGLLRAIRFLQTNWQELVSDISTGTLSSRISDPGIRESMSKILTE
BnaA01.GH3-5.a      LCQHHEVLRVGAVFASGFIRAIKFLEKRWTELVRDIRTGTLSSSITDPSVREAVAKIL.K
BnaA03.GH3-5.b      LCQHQEVLRVGAVFASGFIRAIKFLEKHWIELVRDIRTGTLSSLITDPSVREAVAKIL.K
BnaC01.GH3-5.a      LCQHHEVLRVGAVFASGFIRAIKFLEKHWTELVRDIRTGTLSSSITDPSVREAVAKIL.K
BnaC07.GH3-5.b      LCQYQEVLRVGAVFASGFIRAIKFLEKHWTELVRDIRTGTLSSLITDPSVREAVAKIL.K
BnaA03.GH3-6.a      LCQHNEVLRVGAVFASGFIRAIKFLEKHWPQLVHDIRTGTLNSEITDLSVREAVEEIL.K
BnaAX.GH3-6.b       LCQHKEVLRVGAVFASGFIRAIKFLEKHWPELTRDIRTGTLSSEITDLSVREAVGEIL.K
BnaC03.GH3-6.a      LCQHNEVLRVGAVFASGFIRAIKFLEKHWPELVHDIRTGTLNSEITDLSVREAVEEIL.K
BnaCX.GH3-6.b       LCQHKEVLRVGAVFASGFIRAIKFLEKHWPELTRDIRTGTLSSEITDLSVREAVGEIL.K
BnaA06.GH3-7.a      LVQRDEVTRIGSTFASGMVRVIKVLEDSWKDLCFDIRSGNLSEWITDSGCRNSVSLVLGG
BnaA08.GH3-7.b      LVQRNEVTRMGSIFASVMVRAIKFLEDSWEELCSNIRSGQLSEWITDPGCRDSVSMVLGG
BnaC06.GH3-7.a      LVQRDEVVRISSTFASGMVRVIKVLEDSWKELCLNIRSGYLSEWITDSGCRNAVSMVLGG
BnaA03.GH3-8.a      .ILRDEVVKVGTAFVSTLLRVITFLEKHWKEICTNIRYGHLSEWITDISCRDSVSKILGE
BnaA03.GH3-8.b      LVLRDEVTRVSANFASILVQGITFLENFWKEMCSNIRSGQLSDWITD.SCKGSVSMILGG
BnaA03.GH3-8.c      LVMRDEVTRVSSGFASVLVQGITFLEKFWKEMCSNIRSGQLSDWITNLSCRDSVSKVLGG
BnaA06.GH3-8.d      LVQRDDVVSVGAVFASVLVGVIHFLENYWKELASNIRSGHLSEWITDLGCRDYVSILLGD
```

```
BnaA06.GH3-8.e      LVQRDEVASVGAVFAAVLVQVIHFLENYWKELASNIRFGHVSEWITDLSCRDSVSTVLVE
BnaA09.GH3-8.f      LVQRDEVVSVGALFASVLVQIIRILEKYWKELCSNIRSGRLSDWITDHCCRDSVSIILGE
BnaA09.GH3-8.g      LVQRDEIVSVGAPFASLLVQVIHFLGNYWKELCSNIRSGHVSEWITDLSCRDSVSMIVGE
BnaAX.GH3-8.h       IVQRDEVVKVGAAFVSILVRAITFLEKFWKEICTNIRCGHLSEWITDISCRDSVSKTLGE
BnaAX.GH3-8.i       LVQRD.........................ELARNIRSGHLSEWITDLSCRDSVSIILGE
BnaC01.GH3-8.a      LVMRDEVTGIGAVFASIFVQGMIFLEKTWKDMCSNIRSGQLSDWITDLGCRESVSKILGG
BnaC02.GH3-8.b      IVQRDEVVKVGAAFVSILVRAITFLEKFWKEICINIRCGHLSEWITDISCRDSVSKILGE
BnaC02.GH3-8.c      IVQRDEVVKVGAAFVSILVRAITFLEKFWKEICINIRCGHLSEWITDISCRDSVSKILGE
BnaC02.GH3-8.d      IVQRDEVVKVGAAFVSILVRAITFLEKFWKEICINIRCGHLSEWITDISCRDSVSKILGE
BnaC07.GH3-8.e      LVLRDEVTRVSANFASILVQGITFLENFWKEMCSNIRSGQLSYWITD.NCKDSVSMILGG
BnaC09.GH3-8.f      LRQRDEVVAMAASFASSLVGAVTFFESYWKEICSNIRSGNVSEWITDLSCREAVTNILGG
BnaCX.GH3-8.g       LVQRDEVVSVGAVFASVLVQVIHFLENYWKELASNIRCGHVSEWITDLSCRETVTTILVE
BnaA05.GH3-9.a      LIHRHKVMRLGAVFASAFLRAISYLERKWSQLCQDIRTGHLSPMITDPGCQTAMSSLLAS
BnaC04.GH3-9.a      LIHRHKVMRLGAVFASAFLRAISYLERKWSQLCQDIRTGHLSPMITDPGCQTAMTSLLAS
BnaA03.GH3-10.a     LHFSSQVDFVASGFAYTIVQAFSFLEENWREICADIKEGNLSSRITLPNMRTAVLALIR.
BnaA09.GH3-10.b     LHFSKQVEFVASAFSYTIVQAFSLFEDTWRDICADIKEGSLSSRITLPRMRKAVLALIR.
BnaC09.GH3-10.a     LHFSRQVEFVASAFSYTIVQAFSLFEDTWRDICVDIKEGSLSSRITLPKMRKAVLALIR.
BnaA03.GH3-11.a     ILFRDQVQYVFASFAHGLVHAFRTFEQLWEEIVTDIEDGVLSSRITVPSVRTAMAKLLRG
BnaA04R.GH3-11.b    ILFRHQVQYVFASFAHGLVHAFRTFEQVWEEIVADIKEGVLTSRITVPSVRSAMSKLLR.
BnaA05.GH3-11.c     ILFRDQVQYVFAPFAHGLVHAFRTFEHVWEEIVTDIKHGVLSSRITVPSLRAAMSKLITA
BnaC04.GH3-11.a     ILFRDQVQYVFASFAHGLVHAFRTFEQVWEEIVTDIKEGVLTSRITVPSVRSAMSKLLR.
BnaCX.GH3-11.b      ILFRDQVQYVFASFAHGLVHAFRTFEQLWEEVVTDIEDGVLSSRITVPSVRTAMAKLLSG
BnaA02.GH3-12.a     LVQRDEVVRMGSIFASVMVRAIKFLETYWEELCSNIRSGHLSEWITDLGCHSSVSLVLGG
BnaA03.GH3-12.b     LLQRDEVVRMGSIFASVMVRAIKFLETYWEELCSNIRSGHLSEWITDRGCRSSVSLVLEG
BnaA06.GH3-12.c     LVQRDEVMRIGSTFASGMVRVIKVLQDSWKELCSNIRSGHLSEWITDSGCRNAVSMVTGG
BnaA10.GH3-12.d     LLQRDEVVRMGSIFASVMVRAIKFLETYWEELCSNIRSGRLSEWITDLGCRSSVSLVLGG
BnaC02.GH3-12.a     LVQRDEVVRMGSIFASVMVRAIKFLETYWEELCSNIRSGHLSEWITDLGCRSSVSLVLGG
BnaC03.GH3-12.b     LVQRDEVVRMGSIFASVMVRAIKFLETYWEELCSNIRSGHLSEWITDHGCRSSVSLVLGG
BnaC09.GH3-12.c     LLQRDEVVRMGSIFASVMVRAIKFLETYWEELCSNIRTGRLSEWITDLGCRSSVSLVLGG
BnaA10.GH3-14.a     LIQRGNVSRLGAPFASSFLKVINFLGDHWSELCSNIKTGSVSPWIKDAQCLSGISKFLSA
BnaC09.GH3-14.a     LIQRGNVSRLGAPFASSFLKVINFLGDHWSELCSNIRTGRVSPWIKDAHCLSGISTFLSA
BnaA03.GH3-15.a     LLQRENVSRLGAPFASSFLKVIKFLEDHWKELCSNITTGRVSDWITDPQCLSGVGKFLSA
BnaC03.GH3-15.a     LLQRESVSRLGAPFASSFLKVIKFLEDHWKELCSNIRTGHVSDWITDPQCLSGVGKFLTA
BnaAX.GH3-16.a      LVQRENVARLGAPFASSFLKVINFLEDHWTELCSNIRTGRVSDWITDAQCVSGVSKFLTA
BnaC02.GH3-16.a     LVQRENVARLGAPFASSFLKVINFLEDHWTELCSNIRTGRVSDWITDAQCVSGVSKFLTA
BnaA07.GH3-17.a     LVQRSHVLRVGTVFASAFLRAVKFLEDHYKELCADIRTGTVTSWITEPACRDSVLSVLQG
BnaA08.GH3-17.b     LVQRSHVLRLGAIFASAFLRAVKFLENHFTELCADIRTGTVASWITDSGCRDSALSILNG
BnaA09.GH3-17.c     LVQRSHVLRVGAVFASAFLRAVKFLEDHYKELCADIRTGTVTSWITEPACRDSVLSVLQG
BnaC08.GH3-17.a     LVQRSHVLRLGAIFASAFLRAVKFLENHFTELCADIRTGTVASWITDSGCRDSVLSILNG
BnaCX.GH3-17.b      LVQRSHVLRVGAVFASAFLRAVKFLEDHYKELCADIRTGTITSWITDSACRDSVLSILDG
BnaCX.GH3-17.c      LVQRSHVLRVGTVFASAFLRAVKFLEDHYKELCADIRTGTVTSWITEPACRDSVLSVLQG
BnaA05GH3-18.a      ....NEVVRFGATFAHALVRAIDFLESNWKELCGNIRLGHVSEWITDLGCRSSVSAIIGG
BnaCX.GH3-18.a      ...RNEVVRVGATFPSVLVRVINFLEKYWKELASNIRSGTVSDWITDLSCRDSVSTILGE
BnaA06.GH3-19.a     LVQRDEVVSVGSVFASVLVQVIHFLENYWKELASNIRSGHLSEWITDLSCRESVSTILVE
BnaA09.GH3-19.b     LVQRDEVVSVGTTFPSVLVQIVHFLENYWKELSSNIRSGHVSDWITDLSCRDSVSIILGE
consensus>70        l..r.ev...ga.fas.....i.fle..w.el..#ir.G.ls..!td...r..v..il..
```

```
                      280       290          300       310       320
AtGH3-1           .PDPVLAEFIREC.KSDN...WEKIITRIWPNTKYLDVIVTGAMAQYIPTLEYYS.GG.
AtGH3-2           .PDQELAEFLVGVC.SQEN...WEGIITKIWPNTKYLDVIVTGAMAQYIPTLEYYS.GG.
AtGH3-3           .PDQELADFITSVC.GQDNS..WEGIITKIWPNTKYLDVIVTGAMAQYIPMLEYYS.GG.
AtGH3-4           .PDQELAEFLIGVC.SQEN...WEGIITKIWPNTKYLDVIVTGAMAEYIPMLEYYS.GG.
AtGH3-5           .PSPKLADFVEFEC.KKSS...WQGIITRLWPNTKYVDVIVTGTMSQYIPTLDYYS.NG.
AtGH3-6           .PDPKLADFVESEC.RKTS...WQGIITRLWPNTKYVDVIVTGTMSQYIPTLDYYS.NG.
AtGH3-7           .PHPEAADTIEQIC.NQKC...WKGIITRLWPKAKYIETIVTGSMVQYVPTLNYYS.NNM
AtGH3-8           .PNPELADLIENEC.NNKS...WEGIVPRLWPKAKFIECIATGQMAQHIPTLEFYS.NK.
AtGH3-9           .PNPELASEIEEIC.GRSS...WKGILCQLWPKAKFIEAVVTGSMAQYIPALEFFSQGK.
AtGH3-10          .PNPSLASHIEEICLELETNLGWFGLISKLWPNAKFISSIMTGSMLPYLNKLRHYA.GG.
AtGH3-11          .PNPELAETIRTKCMSLSN...WYGLIPALFPNAKYVYGIMTGSMEPYVPKLRHYA.GD.
AtGH3-12          .PRPELADTIEEIC.NQNS...WKGIVKRLWPNTKYIETVVTGSMGQYVPMLNYYC.ND.
AtGH3-13          .PNPDLASLIEQEC.SKTS...WEAIVKRLWPKAKCVEAVVTGSMAQYIPLLEFYG.GG.
AtGH3-14          .PNPELASLIEQEC.SQTS...WEAIVKRLWPKAKCIEAIVTGTMAQYNPLLEFYS.GG.
AtGH3-15          .PNPELASLIEQEC.SKTS...WEAILKRLWPKAKCIESIITGTMAQYIPLLEFYS.GG.
AtGH3-16          .PDPDLANLIEQEC.SKTS...WEAILSRIWPKAKCIEAVITGTMAQYIPLLEFYG.GG.
AtGH3-17          .PNQELADEIESEC.AEKS...WEGILRRIWPKAKYVEVIVTGSMAQYIPTLEFYS.GG.
AtGH3-18          .PDSILADVIEQEC.SHKS...WEGIITRLWPKAKYIDCIITGQMSQYIPMLEFYS.NK.
AtGH3-19          .PDPELADVIEQEC.SHKS...WEGIITRLWPKAKFIECIVTGQMAQYIPTLDFYS.NK.
BraX.GH3-1.a      .PDQHLAEFIRQEC.QTEN...WEGIITRIWPNTKYLDVIVTGAMAQYIPTLEYYS.GG.
BraX.GH3-2.a      .PDQELAEFLVEVC.SQDN...WEGIVTKIWPNTKYLDVIVTGAMAQYIPTLEYYS.GG.
BraA09.GH3-3.a    .PDQELADYIASVC.CHDSN..WEGVITKIWPNTKYLDVIVTGAMAQYIPMLEYYS.GG.
BraA01.GH3-5.a    .QSPKLAEFVESEC.KKKS...WQGIITRLWPNTKYVDVIVTGTMSQYIPTLDYYS.NG.
BraA03.GH3-5.b    .PSPKLADFVEFEC.KKKS...WHGIITRLWPNTKYVDVIVTGTMSQYIPTLDYYS.NG.
BraA02.GH3-6.a    .PDPKLADFIESEC.MKNS...WQGIITRLWPNTKYVDVIVTGTMSQYIPTLDYYS.NG.
BraA03.GH3-6.b    .PDPRLADFIETEC.RKTS...WQGIITRLWPNTKYVDVIVTGTMSQYIPTLDYYS.NG.
BraA06.GH3-7.a    QPRHKLSDEIESIC.SQKS...WKGIMKKLWPQTLCIEAIATGSMAQYVPTLKHYS.GD.
BraA08.GH3-7.b    .PHPEVANTVEKIC.NEKC...WKGILKRLWPRTKYIETIVTGSMAQYVPTLNYYC.NDM
BraA02.GH3-8.a    .PNPELADLIENEC.NQKS...WEGIIPRLWPKTKFIESIATGQMAQHIPTLKFYS.NN.
BraA03.GH3-8.b    .PNPELADLIENEC.NQKS...WEGIIQRLWPKTKFIESIATGQMAQHIPTLEFYS.NK.
BraA03.GH3-8.c    .PNPQLADIIEDIC.NQKS...WKGIIPQLWPKTKYIECIVTGQMAQHVPLLEFYV.ND.
BraA03.GH3-8.d    .PNPQLAVTIEDIC.NQKS...WKCIIPQLWPRTKFIECIFTGQMAQHIPLLEFYG.ND.
BraA06.GH3-8.e    .PNPELADLIENVC.GQKS...WQGIVSRLWPKAKCIDAIITGSMAQYIPALEFYSNNE.
BraA06.GH3-8.f    .PNPELADLIENVC.GKKS...WQGIVSLLWPKAKCIEAIFTGSMAQYIPTLEFYTNNE.
BraA09.GH3-8.g    .GNSELADKIEEEC.KKKS...WKCIIPRLWPNVKFIQSIVTGQNSQYIPMLEFYS.NK.
BraA09.GH3-8.h    .PNPELADLIENEC.GQES...WQGIVSRLWPKTKCVETIVTGIMAQYIPALEFYS.DK.
BraA10.GH3-8.i    .GNSELADRIEEEC.NTKS...WEGIITRLWPKVKFIQSTVTGQNSQCIPMLEFYS.NN.
BraA09.GH3-10.a   .PNPSLASRIEEVCTELESNVGWLGLIPKLWPNAKYVSSIMTGSMLPYLTKLRHYA.GG.
BraA03.GH3-11.a   .PNPELAETIRTKCMSLSN...WYGLIPALFPNAKYVYGIMTGSMEPYVKKLRHYA.GE.
BraA04.GH3-11.b   .PNPELADMIRTKCLSLSN...WYGLIPALFPNAKYVYGIMTGSMEPYVKKLRHYA.GE.
BraA05.GH3-11.c   .PNPELEDTISDKCLSLSN...WYGLIPALFPNAKYVYGIMTGSMEPYVKKLRHYA.GD.
BraA02.GH3-12.a   .PRPDLADTIETIC.NQSS...WKGIVTRLWPNTKYIETVVTGSMGQYVPTMNYYC.ND.
BraA03.GH3-12.b   .PRPDLADTIETIC.NKNS...WEGIVKRLWPNTKYIETVVTGSMGQYVPTLNYYC.SD.
BraA06.GH3-12.c   QPRPKLSDEIENIC.SLKS...WKGIMKKLWPQTMYIEAIVTGSMAQYVPTLNHYC.DD.
BraA10.GH3-12.d   .PRPDLADTIETIC.NQSS...WKGIVTRIWPNTKYIETVVTGSMGQYVPTLNYYC.SD.
BraA10.GH3-14.a   .PNPELASIIEKEC.SKTS...WEAIVRRLWPNAKCIEAVVTGSMSQYIPMLEFYG.GG.
BraA03.GH3-15.a   .PNPELASLIEKEC.GKTS...WEAIVRRLWPNAKCIEAVVTGSMAQYIPMMDFYC.GG.
BraA07.GH3-15.b   .PNPELASLIEQEC.SKTS...WEAIVRRLWPNAKCIEAVVTGSMAQYIPMMEFYC.GG.
BraA02.GH3-16.a   .PNPDLASLIEKEC.SKTS...WEGIVKRLWPKAKCIEAVVTGSMVQYIPLLEFYG.GG.
BraA07.GH3-17.a   .PNQELADEIESEC.AEKS...WEGILRRLWPKAKYVEVIVTGSMAQYIPTLEFYS.GG.
BraA09.GH3-17.b   .PNQELADEIESEC.AEKS...WEGILRRLWPKAKYVEVIVTGSMAQYIPTLEFYS.GG.
BraAX.GH3-17.c    .PNQELADEIEALF.NAKS...REGILKRIWPKTKYIEVIVTGSMAQYIPTLEFYS.GG.
BraA04.GH3-19.a   .PNAELADKIEREC.CQTS...WEGIIARLWPNIKFIQSVVTGQMSQCIPILEFYS.SK.
BraA06.GH3-19.b   .PNPELADLIENEC.GQTS...WQGIVTRLWPKAKCMEAIITGSMAQHISALEFYS.NK.
BraA09.GH3-19.c   .PNAELADLIEKEC.GQES...WQGIIKRLWPKTKCIETIVTGTMSQHIPALDYYS.NK.
BraA09.GH3-19.d   .PNAELADLIEHECSEQKS...WQGIITRLWPKTKYIETIVTGTMSQHIPALDYYS.NE.
BraAX.GH3-19.e    .PNPELADLIEQEC.SHKS...WEGIVTRLWPKAKRIECVLTGQMAQYIPILDFYC.NK.
BolC09.GH3-1.a    .PDQHLAEFIRQEC.QTEN...WEGIITRIWPNTKYLDVIVTGAMAQYIPTLEYYS.GG.
BolC01.GH3-2.a    .PDQELAEFLVEVC.SQDN...WEGIVTKIWPNTKYLDVIVTGAMAQYIPTLEYYS.GG.
BolC08.GH3-3.a    .PDQELADYITSVC.CHDNN..WEGVITKIWPNTKYLDVIVTGAMAQYIPMLEYYS.GG.
BolC01.GH3-5.a    .PSPKLAEFVESEC.KKKS...WQGIITRLWPNTKYVDVIVTGTMSQYIPTLDYYS.NG.
BolC07.GH3-5.b    .PSPKLADFVESEC.KKKS...WQGIITRLWPNTKYVDVIVTGTMSQYIPTLDYYS.NG.
BolC02.GH3-6.a    .PDPKLADFIESEC.KKNS...WQGIITRLWPNTKYVDVIVTGTMSQYIPTLDYYS.NG.
BolC03.GH3-6.b    .PDPKLADFVETEC.RKTS...WQGIITRLWPNTKYVDVIVTGTMSQYIPTLDYYS.NG.
BolC06.GH3-7.a    QPRPNLSDEIESIC.SQKS...WKGIMKKLWPQTKYIEAIVTGSMVQYIPMLEHYC.SD.
BolC01.GH3-8.a    .PNPQLADQIQDIC.SQKS...WKGIIPQLWPNTKFVEGVITGQMAQYVPALEFYV.DDQ
BolC03.GH3-8.b    .PNPELADLIENEC.DQKS...WEGIIQRLWPKTKFM...TGQMAQHIPTLEFYS.NK.
BolC07.GH3-8.c    .PNPQLADIIEDIC.NQKS...WKGIIPQLWPKTKYIECIVTGQMAQHVPLLEFYV.ND.
BolC08.GH3-8.d    .PNPELADLIEKECTGQES...WQGIITRLWPKTKCIETIVTGTLAQYIPVLEFYS.NK.
BolC09.GH3-8.e    .GNSELADRIEKEC.NTKS...WEGIFTRLWPKVKFIQSTVTGQNSQCIPMLEFYS.NN.
BolC09.GH3-10.a   .PNPSLASRIEDVCAELESNVGWFGLIPKLWPNAKYVSSIMTGSMLTYLTKLRHYA.GG.
BolC03.GH3-11.a   .PNPELAETIRTKCMSLSN...WYGLIPALFPNAKYVYGIMTGSMEPYVKKLRHYA.GE.
BolC04.GH3-11.b   .PNPELAETIRAKCLSLSN...WYGLIPALFPNAKYVYGIMTGSMEPYVKKLRHYA.GE.
BolCX.GH3-11.c    .PNPELAETIRDKCLSLSN...WYGLIPALFPNAKYVYGIMTGSMEPYVKKLRHYA.GD.
BolC03.GH3-12.a   .PRLDLADTIETIC.NKNS...WEGIVKRLWPNTKYIETVVTGSMGQYVPTLNYYC.SD.
BolC09.GH3-12.b   .PRPDLADTIETIC.NQSS...WKGIVTRIWPNTKYIETVVTGSMGQYVPTLNYYC.SD.
BolC09.GH3-14.a   .PNPELASIIEQEC.SKKS...WEAIVRRLWPNAKCIEAVVTGSMSQYIPMLEFYG.GG.
BolC03.GH3-15.a   .PNPELASLIEQEC.GKKS...WEAIVRRLWPNAKCIEAVVTGSMAQYIPMMDFYC.GG.
BolCX.GH3-16.a    .PNPDLASLIEKEC.SKIS...WEGIVKRLWPKAKCIEAVVTGSMVQYIPLLEFYG.GG.
BolC07.GH3-17.a   .PNQELADEIESEC.AEKS...WEGILRRLWPKAKYVEVIVTGSMAQYIPTLEFYS.GG.
BolC08.GH3-17.b   .PNQELADEIEALF.NAKS...REGILKRVWPKTKYIEVIVTGSMAQYIPTLEFYS.GG.
BolC08.GH3-19.a   .PNAELADLIEKEC.GQES...WQGIITRLWPKTKCIETIVTGTMSQHIPALDYYS.NK.
BnaA09R.GH3-1.a   .PDQHLAEFIRQEC.QMEN...WDGIITRIWPNTKYLDVIVTGAMAQYIPTLEYYS.GG.
BnaCX.GH3-1.a     .PDQHLAEFIRQGC.QTEN...WEGIITRIWPNTKYLDVIVTGAMAQYIPTLEYYS.GG.
BnaA01.GH3-2.a    .PDQELAEFLVEVC.SQDN...WEGIVTKIWPNTKYLDVIVTGAMAQYIPTLEYYS.GG.
BnaC01.GH3-2.a    .PDQELAEFLVEVC.SQDN...WEGIVTKIWPNTKYLDVIVTGAMAQYIPTLEYYS.GG.
BnaA09.GH3-3.a    .PDQELADYIASVC.CHDNN..WEGVITKIWPNTKYLDVIVTGAMAQYIPMLEYYS.GG.
BnaC08.GH3-3.a    .PDQELADYITSVC.CHDNN..WEGVITKIWPNTKYLDVIVTGAMAQYISMLEYYS.GG.
BnaA01.GH3-5.a    .QSPKLAEFVESEC.KKKS...WQGIITRLWPNTKYVDVIVTGTMSQYIPTLDYYS.NG.
BnaA03.GH3-5.b    .PSPKLADFVEFEC.KKKS...WQGIITRLWPNTKYVDVIVTGTMSQYIPTLDYYS.NG.
BnaC01.GH3-5.a    .PSPKLAEFVESEC.KKKS...WQGIITRLWPNTKYVDVIVTGTMSQYIPTLDYYS.NG.
BnaC07.GH3-5.b    .PSPKLADFVESEC.QKKS...WQGVITRLWPNTKYVDVIVTGTMSQYIPTLDYYS.NG.
BnaA03.GH3-6.a    .PDPKLADFIETEC.RKTS...WQGIITRLWPNTKYVDVIVTGTMSQYIPTLDYYS.NG.
BnaAX.GH3-6.b     .PDPKLADFIESEC.KKNS...WQGIITRLWPNTKYVDVIVTGTMSQYIPTLDYYS.NG.
BnaC03.GH3-6.a    .PDPKLADFVETEC.RKTS...WQGIITRLWPNTKYVDVIVTGTMSQYIPTLDYYS.NG.
BnaCX.GH3-6.b     .PDPKLADFIESEC.KKNS...WQGIITRLWPNTKYVDVIVTGTMSQYIPTLDYYS.NG.
BnaA06.GH3-7.a    QPRHKLSDEIESIC.SQKS...WKGIMKKLWPQTLCIEAIATGSMAQYVPTLKHYC.GD.
BnaA08.GH3-7.b    .PHPEVANTVEKIC.NEKC...WKGILKRLWPRTKYIETIVTGSMAQYVPTLNYYC.NDM
BnaC06.GH3-7.a    QPRPNLSDEIESIC.SQKS...WKGIMKKLWPQTKYIEAIVTGSMVQYIPMLEHYC.SD.
BnaA03.GH3-8.a    .PNPELADLIENEC.NQKS...WEGIIQRLWPKTKFIESIATGQMAQHIPTLEFYS.NK.
BnaA03.GH3-8.b    .SNPQLADIIEDIC.NQKS...WKGIIPQLWPKTKYIECIVTGQMAQHVPLLEFYV.ND.
BnaA03.GH3-8.c    .PNPQLAVTIEDIC.NQKS...WKCIIPQLWPRTKFIECIFTGQMAQHIPLLEFYG.ND.
BnaA06.GH3-8.d    .PNPELADLIENVC.GEKS...WQGIVSRLWPKAKCIDAIITGSMAQYIPALEFYSNNE.
```

```
BnaA06.GH3-8.e      .PNPELADLIENVC.GKKS...WQGIVSLLWPKAKCIEAIFTGSMAQYIPTLEFYTNNE.
BnaA09.GH3-8.f      .PNPELADLIENEC.GQES...WQGIVSRLWPKTKCVETIVTGIMAQYIPALEFYS.DK.
BnaA09.GH3-8.g      .PNLELADLIENEC.GQES...WQGIISRLWPKTKCIEAIVTGTMAQYIPALEFYS.NK.
BnaAX.GH3-8.h       .PNPELADLIENEC.NQKS...WEGIIPRLWPKTKFIESIATGQMAQHIPTLEFYS.NN.
BnaAX.GH3-8.i       .PNPELADLIKKECTGQES...WQGIITRLWPKTKCIETIVTGTLAQYIPVLEFYS.NK.
BnaC01.GH3-8.a      .PNPQLADQIQDIC.SQKS...WKGIIPQLWPNTKFVEGVITGQMAQYVPALEFYV.DDQ
BnaC02.GH3-8.b      .PNPELADLIENEC.NQKS...WEGIIPRLWPKTKFIESIATGQMAQHIPTLEFYS.NN.
BnaC02.GH3-8.c      .PNPELADLIENEC.NQKS...WEGIIPRLWPKTKFIESIATGQMAQHIPTLEFYS.NN.
BnaC02.GH3-8.d      .PNPELADLIENEC.NQKS...WEGIIPRLWPKTKFIESIATGQMAQHIPTLEFYS.NN.
BnaC07.GH3-8.e      .PNPQLADIIEDIC.NQKS...WKGIIPQLWPKTKYIECIVTGQMAQHVPLLEFYV.ND.
BnaC09.GH3-8.f      .GNSELADNIEEEC.NKKS...WKGIIPRLWPNVKFIQSIVTGQNSQYIPMLEFYS.NK.
BnaCX.GH3-8.g       .PNPELADLIENEC.RQES...WQ.......................ALEFYT.NK.
BnaA05.GH3-9.a      .PNPDLADEIEEIC.GRPS...WKGILCQLWPQAKFIEAVVTGSMAQYIPALEFFSQGK.
BnaC04.GH3-9.a      .PNPDLADEVEEIC.GRPS...WKGILCQLWPQAKFIEAVVTGSMAQYIPALEFFSQGK.
BnaA03.GH3-10.a     .PNPSLASRIEEMCMELESNLGWFGLIPKLWPTAKYVSSIMTGSMLSYLKKLRHYA.GG.
BnaA09.GH3-10.b     .PNPSLASRIEEVCTELESNMGWLGLIPKLWPNAKYVSSIMTGSMLPYLTKLRHYA.GG.
BnaC09.GH3-10.a     .PNPSLASLIEDVCAELESNVGWFGLIPKLWPNAKYVSSIMTGSMLTYLTKLRLYA.GG.
BnaA03.GH3-11.a     .PNPELAETIRNKCMSLSN...WYGLIPALFPNAKYVYGIMTGSMEPYVKKLRHYA.GE.
BnaA04R.GH3-11.b    .PNPELADTFRTKCLSLSN...WYGLIPALFPNAKYVYGIMTGSMEPYVKKLRHYA.GE.
BnaA05.GH3-11.c     .PNPELAETIRDKCLSLSN...WYGLIPALFPNAKYVYGIMTGSMEPYVKKLRHYA.GD.
BnaC04.GH3-11.a     .PNPELAETIRAKCLSLSN...WYGLIPALFPNAKYVYGIMTGSMEPYVKKLRHYA.GE.
BnaCX.GH3-11.b      .PNPELAETIRTKCMSLSN...WYGLIPALFPNAKYVYGIMTGSMEPYVKKLRHYA.GE.
BnaA02.GH3-12.a     .PRPDLADTIETIC.NQSS...WKGIVTRLWPNTKYIETVVTGSMGQYVPTMNYYC.ND.
BnaA03.GH3-12.b     .PRPDLADTIETIC.NKNS...WEGIVKRLWPNTKYIETVVTGSMGQYVPTLNYYC.SD.
BnaA06.GH3-12.c     QPRPKLSDEIENIC.SLKS...WKGIMKKLWPQTMYIEAIVTGSMAQYVPTLNHYC.DD.
BnaA10.GH3-12.d     .PRPDLADTIETIC.NQSS...WKGIVTRIWPNTKYIETVVTGSMGQYVPTLNYYC.SD.
BnaC02.GH3-12.a     .PRPDLADTIETIC.NQSS...WKGIVTRLWPNTKYIETVVTGSMGQYVPTMNYYC.DD.
BnaC03.GH3-12.b     .PRLDLADTIETIC.NKNS...WEGIVKRLWPNTKYIETVVTGSMGQYVPTLNYYC.SD.
BnaC09.GH3-12.c     .PRPDLADTIETIC.NQSS...WKGIVTRIWPNTKYIETVVTGSMGQYVPTLNYYC.SD.
BnaA10.GH3-14.a     .PNPELASIIEKEC.SKTS...WEAIVRRLWPNAKCIEAVVTGSMSQYIPMLEFYG.GG.
BnaC09.GH3-14.a     .PNPELASIIEQEC.SKKS...WEAIVRRLWPNAKCIEAVVTGSMSQYIPMIEFYG.GG.
BnaA03.GH3-15.a     .PNPELASLIEQEC.GKTS...WEAIVRRLWPNAKCIEAVVTGSMAQYIPMMDFYC.GG.
BnaC03.GH3-15.a     .PNPELASLIEQEC.GKKS...WEAIVRRLWPNAKCIEAVVTGSMAQYIPMMDFYC.GG.
BnaAX.GH3-16.a      .PNPDLASLIEKEC.SKTS...WEGIVKRLWPKAKCIEAVVTGSMVQYIPLLEFYG.GG.
BnaC02.GH3-16.a     .PNPDLASLIEKEC.SKTS...WEGIVKRLWPKAKCIEAVVTGSMVQYIPLLEFYG.GG.
BnaA07.GH3-17.a     .PNQELADEIESEC.AEKS...WEGILRRLWPKAKYVEVIVTGSMAQYIPTLEFYS.GG.
BnaA08.GH3-17.b     .PNQELADEIEALF.NAKS...REGILKRIWPKTKYIEVIVTGSMAQYIPTLEFYS.GG.
BnaA09.GH3-17.c     .PNQELADEIESEC.AEKS...WEGILRRLWPKAKYVEVIVTGSMAQYIPTLEFYS.GG.
BnaC08.GH3-17.a     .PNQELADEIEALF.NAKS...REGILKRVWPKTKYIEVIVTGSMAQYIPTLEFYS.GG.
BnaCX.GH3-17.b      .PNQELADEIESEC.AEKS...WEGILRRLWPKAKYVEVIVTGSMAQYIPTLEFYS.GG.
BnaCX.GH3-17.c      .PNQELADEIESEC.AEKS...WEGILRRLWPKAKYVEVIVTGSMAQYIPTLEFYS.GG.
BnaA05GH3-18.a      .PNPELADLIEQEC.SHKS...WEGIVTRLWPKAKRIECVLTGQMAQYIPILDFYC.NK.
BnaCX.GH3-18.a      .PNAELADLIEHECGGQKS...WQGIITRLWPKTKYIETIVTGTMSQHIPALDYYS.NK.
BnaA06.GH3-19.a     .PNPELADLIENEC.GQTS...WQGIVTRLWPKTKCMEAIITGSMAQHISALEFYS.NK.
BnaA09.GH3-19.b     .PNAELADLIEKEC.GQES...WQGIIKRLWPKTKCIETIVTGTMSQHIPALDYYS.NK.
consensus>70        .pnpelad.ie..c....s...w.gii..lwp..kyie.ivtg.m.qyip.lef%.....
```

```
                  330       340       350       360       370
                    .         .         .         .         .
AtGH3-1           LPMACTMYASSECYFGLNLNPMSKPSEVSYTIMPNMAYFEFIPL................
AtGH3-2           LPMACTMYASSESYFGINLKPMCKPSEVSYTIMPNMAYFEFLPH....NHDGD.......
AtGH3-3           LPMACTMYASSESYFGINLKPMCKPSEVSYTIMPNMAYFEFLPH....HEVPT.......
AtGH3-4           LPMASMIYASSESYFGINLNPMCKPSEVSYTIFPNMAYFEFLPH....NHDGD.......
AtGH3-5           LPLVCTMYASSECYFGVNLRPLCKPSEVSYTLIPSMAYFEFLPV....HRNNGVTNSINL
AtGH3-6           LPLVCTMYASSECYFGVNLRPLCKPSEVSYTLIPNMAYFEFLPV....HRNSGVTSSISL
AtGH3-7           LPLISTIYASSETQFGLNLNPMCKPEDVSYTFMPNVSYFEFIPV................
AtGH3-8           LPSISSSYVSSETMFGINMSPLCKPENVSYTFLPNLSYFEFLLV................
AtGH3-9           IPLVCPMYASSETYFGVNVEPLSKPSDVVFTLLPNMCYFEFIPLGKNGTLSFDLDDDEQV
AtGH3-10          LPLVSADYGSTESWIGVNVDPHLPPEDVSFAVIPTFSYFEFIPL................
AtGH3-11          LPLVSHDYGSSEGWIAANVTPRLSPEEATFAVIPNLGYFEFLPV................
AtGH3-12          LPLVSTTYGSSETTFGINLDPLCKPEDVSYTFMPNMSYFEFIPM................
AtGH3-13          LPLISSWYGSSECFMGVNVNPLCKPSDVSYTIIPSMAYFEFLEV................
AtGH3-14          LPVISTFYGSSECFFGLNLNPLSKPNEVSYTIIPCMAYFEFLEV................
AtGH3-15          LPLTSSFYGSSECFMGVNFNPLCKPSDVSYTIIPCMGYFEFLEV................
AtGH3-16          LPLVSSWYGSSECFIGINLNPLSKPSDVSYTIIPSMGYFEFIEV................
AtGH3-17          LPLVSTMYASSECYFGINLNPLCDPADVSYTLLPNMAYFEFLPVDDKSHEEIHFATHSNT
AtGH3-18          LPIVSTTYGSSESTFGMNVDPLCKPQDTSYTCAPNISYFEFLPV................
AtGH3-19          LPIVSMVYGSSESIFGVNVDPLSKPQDVSYTFLPNISYFEFLPI................
BraX.GH3-1.a      LPMACTMYASSECYFGLNLNPMSKPSEVSYTIMPNMAYFEFIPL................
BraX.GH3-2.a      LPMACTMYASSESYFGINLKPMCKPSEVSYTIMPNMAYFEFLPH........D.......
BraA09.GH3-3.a    LPMACTMYASSESYFGINLKPMCKPSEVSYTIMPNMAYFEFLPH....EDATE.......
BraA01.GH3-5.a    LPLVCTMYASSECYFGVNLRPLCKPSEVSYTLIPTMAYFEFLPV....HRNTGVTNSINL
BraA03.GH3-5.b    LPLVCTMYASSECYFGVNLRPLCKPSEVSYTLIPTMAYFEFLPV....HRNTGVTNSINL
BraA02.GH3-6.a    LPLVCTMYASSECYFGVNLRPLCKPSEVSYTLIPTMAYFEFLPV....HRNNGVTSSISL
BraA03.GH3-6.b    LPLVCTMYASSECYFGVNLRPVCKPSEVSYTLIPTMAYFEFLPV....HRNSGVTSSISL
BraA06.GH3-7.a    VPLVSTIYASSESMFGINTDPLCQPENTSYTLMPNISYFEFIPT................
BraA08.GH3-7.b    LPLVSTIYASSETTFGLNLNPLCKPEDVSYTIMPNVSYFEFIPL................
BraA02.GH3-8.a    LPLISSSYVSSETMFGINMNPLCKPQDVSYTFMPNFSYFEFLLV................
BraA03.GH3-8.b    LPLISSSYVSSETMFGININPLCKPQDVSYTFMPHFSYFEFLLV................
BraA03.GH3-8.c    LPLVSPNYASSEAMFGVNLNPLCKPQDVSYTFLPNMSYFEFIPV................
BraA03.GH3-8.d    LPLVSRNYMSSEAFFGVNLNPLCKPQDVSYTFMPNMSYFEFIPV................
BraA06.GH3-8.e    LPLVSLRYASSEAYFGLNLEPLSKPQHVSYTFLPNMSYFEFIDV................
BraA06.GH3-8.f    LSLVSLRYASSEAFFGLNLEPLSK..NVSYTFLPNMSYFEFLDV................
BraA09.GH3-8.g    VHLFSPAYGSSETMFGVNVNPLCKPEDVSYTFMPNISYFEFILA................
BraA09.GH3-8.h    LPIVSSIYGSSETFFGLNVNPLCKPQHVSYTFLPNMSYFEFIHV................
BraA10.GH3-8.i    VPLISTVYASSETMFGINMNPFCKPQDVSYTFIPTLSYFEFILA................
BraA09.GH3-10.a   LPLVSADYGSTESWIGVNVDPQLPPEDVSFAVIPTFSYFEFIPL................
BraA03.GH3-11.a   LPLVSHDYGSSEGWIAANVRPRLSPEEATFAVVPNLGYFEFLPV................
BraA04.GH3-11.b   LPLVSHDYGSSEGWIAANVTPRLSPEEATFAVVPNLGYFEFLPV................
BraA05.GH3-11.c   LPLVSHDYGSSEGWIAANVTPRLCPEEATFAVIPNLGYFEFLPV................
BraA02.GH3-12.a   LPLVSTTYGSSETTFGINVDPLCEPEDVSYAFMPNMSYFEFITM................
BraA03.GH3-12.b   LPLVSTTYGSSETTFGINVDPLCKPEDVSYAFMPNMSYFEFITM................
BraA06.GH3-12.c   LPLVSTTYASSESIFGINIDPLCKPEDISYTLMPNISYFEFIPV................
BraA10.GH3-12.d   LPLVSTTYGSSETTFGINVDPLCKPEDVSYAFMPNMSYFEFITM................
BraA10.GH3-14.a   LPVISLFYGSSECLFGLNVNPLCKPCDVSYTTVPSMAYFEFLEV................
BraA03.GH3-15.a   LPLVSSFYASSECFLGLNLNTLRKPSDAAYTIIPSMAYFEFIEV................
BraA07.GH3-15.b   LPLISTFYVSSECSVGLNLDPLSKPCDVSYTTIPSMAYFEFFEI................
BraA02.GH3-16.a   LPLISSWYGASECFIGINVDPLSKPCDVSYTIVPSMAYFEFLEV................
BraA07.GH3-17.a   LTLVSTMYASSECYFGINLNPLCDPSDVSYTLLPNMAYFEFLPVDDKSHEEIHFASQSNA
BraA09.GH3-17.b   LPLVSTMYASSECYFGINLNPLCDPSDVSYTLLPNMAYFEFLPVDDKSHEEIHFASHSNT
BraAX.GH3-17.c    LPLVSVMYASSECFFGLNINPLCKPSDVSYTLLPNMAYFEFLPVDDKSHEETHLESRST.
BraA04.GH3-19.a   LPLVSLNYSASETLFGVNVNPLCKPQDVSYTCVPNTSYFEFLPV................
BraA06.GH3-19.b   LPLVSPIYGSSEAFFGLNLEPLCKPQHVSYTFLPNMSYFEFIDV................
BraA09.GH3-19.c   LPLVSKVYASSEVFYGLNLNPLSKPQHVSYTFLPNMSYFEFIRV................
BraA09.GH3-19.d   LPLVSKVYASSEAFFGINVNPLSKPQHVSYTFLPNMSYFEFKEV................
BraAX.GH3-19.e    LPLVSTVYGSSESIFGFNVDPLCKPQDVSYTFVPNTSYFEFLPV................
BolC09.GH3-1.a    LPMACTMYASSECYFGLNLNPMSKPSEVSYTIMPNMAYFEFIPL................
BolC01.GH3-2.a    LPMACTMYASSESYFGINLKPMCKPSEVSYTIMPNMAYFEFLPH........E.......
BolC08.GH3-3.a    LPMACTMYASSESYFGINLKPMCKPSEVSYTIMPNMAYFEFLPH....EDAP........
BolC01.GH3-5.a    LPLVCTMYASSECYFGVNLRPLCKPSEVSYTLIPTMAYFEFLPV....HRNTGVTNSINL
BolC07.GH3-5.b    LPLVCTMYASSECYFGVNLRPLCKPSEVSYTLIPTMAYFEFLPV....YRNTGVTNSINL
BolC02.GH3-6.a    LPLVCTMYASSECYFGVNLRPLCKPSEVSYTLIPTMAYFEFLPV....HRNNGVTSSISL
BolC03.GH3-6.b    LPLVCTMYASSECYFGVNLRPLCKPSEVSYTLIPTMAYFEFLPV....HRNSGVTSSISL
BolC06.GH3-7.a    LPVVSTIYASSESIFGINTYPLCKPEDISYTLMPNISYFEFIPM................
BolC01.GH3-8.a    LPLYSPAYSSSESPFAVNMNPLCKPQDISYTFIPNMSYFEFLPI................
BolC03.GH3-8.b    LPLISSSYVSSETMFGINMNPLCKPQDVSYTFMPHFSYFEFLLV................
BolC07.GH3-8.c    LPLVSPNYASSEAMFGVNLNPLCKPQDVSYTFLPNMSYFEFIPV................
BolC08.GH3-8.d    LPLVSKNYASSETFFGINLNPLSKPEHVSYTFLPNMAYFEFIDV................
BolC09.GH3-8.e    VPLISTVYASSETMFGINMNPFCKPQDVSYTFIPTLSYFEFILA................
BolC09.GH3-10.a   LPLVSADYGSTESWIGVNVDPQLPPEDVSFAVIPTFSYFEFIPL................
BolC03.GH3-11.a   LPLVSHDYGSSEGWIAANVKPRLSPEEATFAVVPNLGYFEFLPV................
BolC04.GH3-11.b   LPLVSHDYGSSEGWIAANVTPRLSPEEATFAVVPNLGYFEFLPV................
BolCX.GH3-11.c    LPLVSHDYGSSEGWIAANVTPRLSPEEATFAVIPNLGYFEFLLV................
BolC03.GH3-12.a   LPLVSTTYGSSETTFGINVDPLSKPEDVSYAFMPNMSYFEFITM................
BolC09.GH3-12.b   LPLVSTTYGSSETTFGINVDPLCKPEDVSYAFMPNMSYFEFITM................
BolC09.GH3-14.a   LPVISLFYGSSECLFGLNINPLSKPCDVSYTTVPSMAYFEFLEV................
BolC03.GH3-15.a   LPLISSFYASSECFLGLNLNTLRKPSDAAYTIIPSMAYFEFIEV................
BolCX.GH3-16.a    LPLISSWYGASECFIGINVDPLTKPCDVSYTIVPSMAYFEFLEV................
BolC07.GH3-17.a   LPLVSTMYASSECYFGINLNPLCDPSDVSYTLLPNMAYFEFLPVDDKSHEEIHFASQSNA
BolC08.GH3-17.b   LPLVSVMYASSECFFGLNINPLCKPSDVSYTLLPNMAYFEFLPVDDKSHEETQLESRST.
BolC08.GH3-19.a   LPLVSKVYASSEAFYGLNVNPLSKPQHVSYTFLPNMSYFEFIRV................
BnaA09R.GH3-1.a   LPMACTMYASSECYFGLNLNPMSKPSEVSYTIMPNMAYFEFIPL................
BnaCX.GH3-1.a     LPMACTMYASSECYFGLNLNPMSKPSEVSYTIMPNMAYFEFIPL................
BnaA01.GH3-2.a    LPMACTMYASSESYFGINLKPMCKPSEVSYTIMPNMAYFEFLPH........D.......
BnaC01.GH3-2.a    LPMACTMYASSESYFGINLKPMCKPSEVSYTIMPNMAYFEFLPH........E.......
BnaA09.GH3-3.a    LPMACTMYASSESYFGINLKPMCKPSEVSYTIMPNMAYFEFLPH....EDATE.......
BnaC08.GH3-3.a    LPMACTMYASSESYFGINLKPMCKPSEVSYTIMPNMAYFEFLPH....EDAP........
BnaA01.GH3-5.a    LPLVCTMYASSECYFGVNLRPLCKPSEVSYTLIPTMAYFEFLPV....HRNT........
BnaA03.GH3-5.b    LPLVCTMYASSECYFGVNLRPLCKPSEVSYTLIPTMAYFEFLPV....HRNTGVTNSINL
BnaC01.GH3-5.a    LPLVCTMYASSECYFGVNLRPLCKPSEVSYTLIPTMAYFEFLPV....HRNTGVTNSINL
BnaC07.GH3-5.b    LPLVCTMYASSECYFGVNLRPLCKPSEVSYTLIPTMAYFEFLPV....YRNTGVTNSINL
BnaA03.GH3-6.a    LPLVCTMYASSECYFGVNLRPVCKPSEVSYTLIPTMAYFEFLPV....HRNSG.......
BnaAX.GH3-6.b     LPLVCTMYASSECYFGVNLRPLCKPSEVSYTLIPTMAYFEFLPV....HRNNGVTSSISL
BnaC03.GH3-6.a    LPLVCTMYASSECYFGVNLRPLCKPSEVSYTLIPTMAYFEFLPV....HRNSGVTSSISL
BnaCX.GH3-6.b     LPLVCTMYASSECYFGVNLRPLCKPSEVSYTLIPTMAYFEFLPV....HRNNGVTSSISL
BnaA06.GH3-7.a    VPLVSTIYASSESMFGINTDPLCQPENTSYTLMPNISYFEFIPT................
BnaA08.GH3-7.b    LPLVSTIYASSETTFGLNLNPLCKPEDVSYTIMPNVSYFEFIPL................
BnaC06.GH3-7.a    LPVVSTIYASSESIFGINTYPLCKPEDISYTLMPNISYFEFIPM................
BnaA03.GH3-8.a    LPLISSSYVSSETMFGININPLCKPQDVSYTFMPHFSYFEFLLV................
BnaA03.GH3-8.b    LPLVSPNYASSEAMFGVNLNPLCKPQDVSYTFLPNMSYFEFIPV................
BnaA03.GH3-8.c    LPLVSRNYMSSEAFFGVNLNPLCKPQDVSYTFMPNMSYFEFIPV................
BnaA06.GH3-8.d    LPLVSLRYASSESYFGLNLEPLSKPQHVSYTFLPNMSYFEFIDV................
```

```
BnaA06.GH3-8.e      LSLVSLRYASSEAFFGLNLEPLSK..NVSYTFLPNMSYFEFLDV................
BnaA09.GH3-8.f      LPIVSSIYGSSETFFGLNVNPLCKPQHVSYTFLPNMSYFEFIHV................
BnaA09.GH3-8.g      LPLVSRTYASSEAFFGINVNPLSKPQHVSYTFLPNMSYFEFIDV................
BnaAX.GH3-8.h       LPLISSSYVSSETMFGINMNPLCKPQDVSYTFMPNFSYFEFLLV................
BnaAX.GH3-8.i       LPLVSKNYASSETFFGINLNPLSKPEHVSYTFLPNMAYFEFIDV................
BnaC01.GH3-8.a      LPLYSPAYSSSESPFAVNMNPLCKPQDISYTFIPNMSYFEFLPI................
BnaC02.GH3-8.b      LPLISSSYVSSETMFGINMNPLCKPQDVSYTFMPNFSYFEFLLV................
BnaC02.GH3-8.c      LPLISSSYVSSETMFGINMNPLCKPQDVSYTFMPNFSYFEFLLV................
BnaC02.GH3-8.d      LPLISSSYVSSETMFGINMNPLCKPQDVSYTFMPNFSYFEFLLV................
BnaC07.GH3-8.e      LPLVSPNYASSEAMFGVNLNPLCKPQDVSYTFLPNMSYFEFIPV................
BnaC09.GH3-8.f      VHLFSPAYGSSETMFGVNVNPLCKPEDVSYTFMPNISYFEFILA................
BnaCX.GH3-8.g       LPLVSKSYASSEAFFGLNLEPLSK..HVSYTFLPNMSYFEFIDV................
BnaA05.GH3-9.a      IPLVCPMYASSETYFGVNVMPLSKPSDVVFTLLPNMCYFEFIPLGKNGTLSFDVEDEEVV
BnaC04.GH3-9.a      IPLVCPMYASSETYFGVNVKPLSKPSDVVFTLLPNMCYFEFISLGKNGTLSFDVEDEEVV
BnaA03.GH3-10.a     LPLVSGNYVSSESLIGVNVDPQLPPEDVSFAVIPTFSYFEFIPL................
BnaA09.GH3-10.b     LPLVSADYGSTESWIGVNVDPQLPPEDVSFAVIPTFSYFEFIPL................
BnaC09.GH3-10.a     LPLVSADYGSTESWIGVNVDPQLPPEDVSFAVIPTFSYFEFIPL................
BnaA03.GH3-11.a     LPLVSHDYGSSEGWIAANVRPRLSPEEATFAVVPNLGYFEFLPL................
BnaA04R.GH3-11.b    LPLVSHDYGSSEGWIAANVTPRLSPEEATFAVVPNLGYFEFLPV................
BnaA05.GH3-11.c     LPLVSHDYGSSEGWIAANVTPRLSPEEATFAVIPNLGYFEFLPV................
BnaC04.GH3-11.a     LPLVSHDYGSSEGWIAANVTPRLSPEEATFAVVPNLGYFEFLPV................
BnaCX.GH3-11.b      LPLVSHDYGSSEGWIAANVRPRLSPEEATFAVVPNLGYFEFLPL................
BnaA02.GH3-12.a     LPLVSTTYGSSETTFGINVDPLCKPEDVSYAFMPNMSYFEFITM................
BnaA03.GH3-12.b     LPLVSTTYGSSETTFGINVDPLCKPEDVSYAFMPNMSYFEFITM................
BnaA06.GH3-12.c     LPLVSTTYASSESIFGINIDPLCKPEDISYTLMPNISYFEFIPV................
BnaA10.GH3-12.d     LPLVSTTYGSSETTFGINVDPLCKPEDVSYAFMPNMSYFEFITM................
BnaC02.GH3-12.a     LPLVSTTYGSSETTFGINVDPLCKPEDVSYAFMPNMSYFEFITM................
BnaC03.GH3-12.b     LPLVSTTYGSSETTFGINVDPLCKPEDVSYAFMPNMSYFEFITM................
BnaC09.GH3-12.c     LPLVSTTYGSSETTFGINVDPLCKPEDVSYAFMPNMSYFEFITM................
BnaA10.GH3-14.a     LPVISLFYGSSECLFGLNVNPLCKPCDVSYTTVPSMAYFEFLEV................
BnaC09.GH3-14.a     LPVISLFYGSSECLFGLNINPLSKPCDVSYTTVPSMAYFEFLEV................
BnaA03.GH3-15.a     LPLVSSFYASSECFLGLNLNTLRKPSDAAYTIIPSMAYFEFIEV................
BnaC03.GH3-15.a     LPLISSFYASSECFLGLNLNTLRKPSDAAYTIIPSMAYFEFIEV................
BnaAX.GH3-16.a      LPLISSWYGASECFIGINVDPLSKPCDVSYTIVPSMAYFEFLEV................
BnaC02.GH3-16.a     LPLISSWYGASECFIGINVDPLTKPCDVSYTIVPSMAYFEFLEV................
BnaA07.GH3-17.a     LPLVSTMYASSECYFGINLNPLCDPSDVSYTLLPNMAYFEFLPVDDKSHEEIHFASHSNT
BnaA08.GH3-17.b     LPLVSVMYASSECFFGLNINPLCKPSDVSYTLLPNMAYFEFLPVDDKSHEETQLESH...
BnaA09.GH3-17.c     LPLVSTMYASSECYFGINLNPLCDPSDVSYTLLPNMAYFEFLPVDDKSHEEIHFASHSNT
BnaC08.GH3-17.a     LPLVSVMYASSECFFGLNINPLCKPSDVSYTLLPNMAYFEFLPVDDKSHEETQLESH...
BnaCX.GH3-17.b      LPLVSTMYASSECYFGINLNPLCDPSDVSYTLLPNMAYFEFLPVDDKSHEEIHFASHSNT
BnaCX.GH3-17.c      LPLVSTMYASSECYFGINLNPLCDPSDVSYTLLPNMAYFEFLPI........HFASHSNT
BnaA05GH3-18.a      LPLVSTVYGSSESIFGFNVDPLCKPQDVSYTFVPNTSYFEFLPV................
BnaCX.GH3-18.a      LPLVSKVYASSEAFFGMNVNPLSKPQHVSYTFLPNMSYFEFIEV................
BnaA06.GH3-19.a     LPLVSPFYGSSEAFFGLNLEPLCKPQHVSYTFLPNMSYFEFIEV................
BnaA09.GH3-19.b     LPLVSKVYASSEVFYGLNLNPLSKPQHVSYTFLPNMSYFEFIRV................
consensus>70        lplvs..Y.ssE..fg.N..pl.kp.dvs%t..P.m.YFEF...................
```

```
                                                       380       390       400
                                                          .         .         .
AtGH3-1            .................GGTK..........AVELVDVNIGKEYELVVTTYAG.LCRYR
AtGH3-2            GAAEAS...........LDETS..........LVELANVEVGKEYELVITTYAG.LYRYR
AtGH3-3            ..................EKSE..........LVELADVEVGKEYELVITTYAG.LNRYR
AtGH3-4            GGV...............EATS..........LVELADVEVGKEYELVITTYAG.LYRYR
AtGH3-5            PKALTE...........KEQQE..........LVDLVDVKLGQEYELVVTTYAG.LCRYR
AtGH3-6            PKALTE...........KEQQE..........LVDLVDVKLGQEYELVVTTYAG.LYRYR
AtGH3-7            .D...............GDKND..........VVDLADVKLGCCYEAVVTNFSG.LYRIR
AtGH3-8            .D...A...........GDKTE..........IVDLVDVKLGCYYEPLVTNHSG.LHRYK
AtGH3-9            PC...................DK..........VVDLVNVKLGRYYELVVTTFAG.LYRYR
AtGH3-10           .YRRQN.QSDICIDGDFVEDK...........PVPLSQVKLGQEYELVLTTFTG.LYRYR
AtGH3-11           .SETGE...........GEEK...........PVGLTQVKIGEEYEVVITNYAG.LYRYR
AtGH3-12           .D...G...........GDKND..........VVDLEDVKLGCTYEPVVTNFAG.LYRMR
AtGH3-13           .....K...........KDQQEAGLDPIENHVVVDLVDVKIGHDYEPVVTTFSG.LYRYR
AtGH3-14           .....E...........KDY.ESGHDPAENPVVVDLVDVKIGHDYEPVVTTFAG.LYRYR
AtGH3-15           .....E...........KDHQEAGHDPTEKPVVVDLVDVKIGHDYEPVVTTFSG.LYRYR
AtGH3-16           .....V...........KDRQEAGHVPA.DPVVVDLVDVKIGHDYELLVTTFSG.LYRYR
AtGH3-17           DDDDDA...........LKEDL..........IVNLVNVEVGQYYEIVITTFTG.LYRYR
AtGH3-18           .D..HK...........GDMAS..........IVDLVDVKLGCYYEPVVTNYFG.LHRYL
AtGH3-19           .D..HE...........EDMNT..........IVDLVGVKLGCYYETVVTSYFG.LHRYL
BraX.GH3-1.a       ..................GGSK..........AVELVDVKIGKEYELVVTTYAG.LCRYR
BraX.GH3-2.a       GAAG..............DETE..........LVELADVEVGKEYELVITTYAG.LCRYK
BraA09.GH3-3.a     ..................RTTE..........LVELADVEVGKEYELVITTYAG.LYRYR
BraA01.GH3-5.a     PKALTE...........KEQQE..........LVDLVDVKLGQEYELVVTTYAG.LCRYR
BraA03.GH3-5.b     PKALTE...........KEQQE..........LVDLVDVKLGQEYELVVTTYAG.LCRYR
BraA02.GH3-6.a     PKALTE...........KEQKE..........LVDLVDVKLGQEYELVVTTYAG.LYRYR
BraA03.GH3-6.b     PKALTE...........KEQQE..........LVDLVDVKLGQEYELVVTTYAG.LYRYR
BraA06.GH3-7.a     .E...............GGNGD..........VVDLADVKLGCSYQLLVTNLWG.LYRMR
BraA08.GH3-7.b     .D...............GDEND..........VVDLADLKLGCSYELVVTNFSG.LYRIR
BraA02.GH3-8.a     .D...A...........GDKVE..........IVDLVDVKLGSHYEPLVTNHSG.LHRHK
BraA03.GH3-8.b     .D...A...........GDEVE..........IVDLVDVKLGCHYETLVTNHSG.LHRYK
BraA03.GH3-8.c     .G...E...........G.KDT..........IFDLVNVKLGLYYELVVTNYAG.LHRYR
BraA03.GH3-8.d     .G...E...........G.DDT..........IVDLVNVKLGRYYELIVTNYAG.LHRYR
BraA06.GH3-8.e     .D...................GE..........IVDLVNVKLGHYYEPF.....G.LHRCR
BraA06.GH3-8.f     .D...G...........GAEGE..........IVDLVNVKLGRYYEILVTNFSG.LHRCR
BraA09.GH3-8.g     .D...K...........GNEGE..........IVDLVNVEIGSYYEPLITNYYG.LHRYR
BraA09.GH3-8.h     .D.ADG...........EATGE..........IVDLVDVKSGGYYEPLVTNYSG.LYRCR
BraA10.GH3-8.i     .D...E...........GNKGE..........IVDLGNVKIGSYYEPLITNYYG.LHRYR
BraA09.GH3-10.a    .YRQQTHQQDLCSEGDFVEEK...........PVPLSQVKLGQEYELVLTTFTG.LYRYR
BraA03.GH3-11.a    .SETGE...........TEQEP..........PVGLTEVKIGQEYEVVITNYAG.LYRYR
BraA04.GH3-11.b    .S...E...........TEKE...........PVGLTDVKIGEEYEVVITNYAG.LYRYK
BraA05.GH3-11.c    .SETGE...........GEQE...........PVGLTEVKVGQEYEVVLTNYAG.LYRYR
BraA02.GH3-12.a    .D...............GDKRD..........VVDLQDVKLGCTYEPVVTNFSG.LYRMR
BraA03.GH3-12.b    .D...............GDKRD..........VVDLQDVKLGCTYEPVVTNFSG.LYRMR
BraA06.GH3-12.c    .E...............EGSDD..........VVDLADVKLGCSYQLLITNLWG.LYRMR
BraA10.GH3-12.d    .D...............GEKRD..........VVDLHDVKIGCTYEPVVTNFAG.LYRMR
BraA10.GH3-14.a    .....K...........KD.QEAGHDPLVNPVVIDLVDVKVGHDYEPVVTTFSG.LYRYR
BraA03.GH3-15.a    .....E...........KDHQETSHDPTKN..IVDLVDVKVGHDYEPVITTFSG.LYRYR
BraA07.GH3-15.b    .....K...........KYHQETGHDPV....VVDLVDVKVGHDYEPVITTFSG.LYRYR
BraA02.GH3-16.a    .....E...........KDHQETGHNPTKNPVVVDLADVKIGHDYELIITTFSG.LYRYR
BraA07.GH3-17.a    .EDDDS...........IKEDL..........IVDLVNVEVGRYYEIVITTFTG.LYRYR
BraA09.GH3-17.b    DDDDDA...........LKEDL..........IVDLVNVEVGRYYEIVITTFTG.LYRYR
BraAX.GH3-17.c     .DDVSL...........TDKDR..........IVDLVSVEIGRYYELIITTFAG.LYRYR
BraA04.GH3-19.a    .D...E...........GNNAQ..........VVDLVDVKLGHLYDPV..........
BraA06.GH3-19.b    .DVEGG...........TTSGE..........VLDLVDVKLGRYYELLVTNFSG.LHRCR
BraA09.GH3-19.c    .D.ADG...........EDTSE..........IVDLVDVTLGYYYEPLVTNYSG.LHRCR
BraA09.GH3-19.d    .D.DDG...........ETTGE..........IVDLVNVKLGSYYEPLVTNFSG.LHRCR
BraAX.GH3-19.e     .D..HE...........GDMTS..........IVDLVNVKLGCYYEPVVTTCFG.LNRYL
BolC09.GH3-1.a     ..................GGSK..........AVELVDVKIGKEYELVVTTYAG.LCRYR
BolC01.GH3-2.a     .GAG..............DETE..........LVELADVEVGKEYELVITTYAG.LCRYK
BolC08.GH3-3.a     ..................ERTE..........LVELADVEVGKEYELVITTYAG.LYRYR
BolC01.GH3-5.a     PKALTE...........KEQQE..........LVDLVDVKLGQEYELVVTTYAG.LCRYR
BolC07.GH3-5.b     PKALTE...........KEQQE..........LVDLVDVKLGQEYELVVTTYAG.LCRYR
BolC02.GH3-6.a     PKALTE...........KEQQE..........LVDLVDVKLGQEYELVVTTYAG.LYRYR
BolC03.GH3-6.b     PKALTE...........KEQQE..........LVDLVDVKLGQEYELVVTTYAG.LYRYR
BolC06.GH3-7.a     .E...............GDNGD..........VLDLADVKLGSSYKLLVTNLWG.LYRMR
BolC01.GH3-8.a     .D...E...........GNDDA..........IVDLVDVKLGSYYELVVTTYSG.LHRCK
BolC03.GH3-8.b     .D...A...........GDEVK..........IVDLVDVKLGCHYESLVTNHS......
BolC07.GH3-8.c     .G...E...........G.NDT..........IVDLVNVKLGRYYELVVTNYAG.LHRYR
BolC08.GH3-8.d     .G.VDG...........GTEGE..........IVDLVDVKLGHYYEPLVTNYYG.LHRCR
BolC09.GH3-8.e     .D...E...........GNKGE..........IVDLVNVKIGSYYEPLITNYYG.LHRYR
BolC09.GH3-10.a    .YRQQT.QQDLCSEGDFVEEK...........PVPLSQVKLGQEYELVLTTFTG.LYRYR
BolC03.GH3-11.a    .SETGE...........TEQEP..........PVGLTEVKIGQEYEVVITNYAG.LYRYR
BolC04.GH3-11.b    .SETEE...........TEEE...........PVCLTEVKIGQEYEVVITNYAG.LYRYK
BolCX.GH3-11.c     .SETGE...........GEQE...........PVGLTEVKVGREYEVVLTNYAG.LYRYR
BolC03.GH3-12.a    .D...............GDKRD..........VVDLQDVKLGCTYEPVVTNFSG.LYRMR
BolC09.GH3-12.b    .D...............GDKRD..........VVDLHDVKVGCTYEPVVTNFAG.LYRMR
BolC09.GH3-14.a    .....K...........KD.QEAGHDPLVNPVVVDLVDVKVGHDYEPVVTTFSG.LYRYR
BolC03.GH3-15.a    .....E...........KDHQETSHDPTKN..IVDLVDVKVGHDYEPVITTFSG.LYRYR
BolCX.GH3-16.a     .....E...........KDHQETGHNPTKNPVVVDLADVKIGHDYELIITTFSG.LYRYR
BolC07.GH3-17.a    .EDDDS...........IKEDL..........IVDLVNVEVGRYYEIVITTFTG.LYRYR
BolC08.GH3-17.b    .DDVSL...........MDKDR..........IVDLVNVEIGRYYELIITTFAG.LYRYR
BolC08.GH3-19.a    .D.ADG...........EDTSE..........IVDLVDVTLGHYYEPLVTNYSG.LHRCK
BnaA09R.GH3-1.a    ..................GGSK..........AVELVDVKIGKEYELVVTTYAG.LCRYR
BnaCX.GH3-1.a      ..................GGSK..........AVELVDVKIGKEYELVVTTYAG.LCRYR
BnaA01.GH3-2.a     GAAG..............DETE..........LVELADVEVGKEYELVITTYAG.LCRYK
BnaC01.GH3-2.a     GGAG..............DETE..........LVELADVEVGKEYELVITTYAG.LCRYK
BnaA09.GH3-3.a     ..................RTTE..........LVELADVEVGKEYELVITTYAG.LYRYR
BnaC08.GH3-3.a     ..................ERTE..........LVELADVEVGKEYELVITTYAG.LYRYR
BnaA01.GH3-5.a     ..ALTE...........KEQQE..........LVDLVDVKLGQEYELVVTTYAG.LCRYR
BnaA03.GH3-5.b     PKALTE...........KEQQE..........LVDLVDVKLGQEYELVVTTYAG.LCRYR
BnaC01.GH3-5.a     PKALTE...........KEQQE..........LVDLVDVKLGQEYELVVTTYAG.LCRYR
BnaC07.GH3-5.b     PKALTE...........KEQQE..........LVDLVDVKLGQEYELVVTTYAG.LCRYR
BnaA03.GH3-6.a     ..ALTE...........KEQQD..........LVDLVDVKLGQEYELVVTTYAG.LYRYR
BnaAX.GH3-6.b      PKALTE...........KEQQE..........LVDLVDVKLGQEYELVVTTYAG.LYRYR
BnaC03.GH3-6.a     PKALTE...........KEQQE..........LVDLVDVKLGQEYELVVTTYAG.LYRYR
BnaCX.GH3-6.b      PKALTE...........KEQQE..........LVDLVDVKLGQEYELVVTTYAG.LYRYR
BnaA06.GH3-7.a     .E...............GGNGD..........VVDLADVKLGCSYQLLVTNLWG.LYRMR
BnaA08.GH3-7.b     .D...............GDEND..........VVDLADLKLGCSYELVVTNFSG.LYRIR
BnaC06.GH3-7.a     .E...............GDNGD..........VLDLADVKLGSSYKLLVTNLWG.LYRMR
BnaA03.GH3-8.a     .D...A...........GDEVE..........IVDLVDVKLGCHYETLVTNHS......
BnaA03.GH3-8.b     .G...E...........G.KDT..........IVDLVNVKLGLYYELVVTNYAG.LHRYR
BnaA03.GH3-8.c     .G...E...........G.DDT..........IVDLVNVKLGRYYELIVTNYAG.LHRYR
BnaA06.GH3-8.d     .D...................GE..........IVDLVNVKLGHYYEPLVTNFSG.LHRCR
```

```
BnaA06.GH3-8.e     .D...G...........GAEGE..........IVDLVNVKLGRY................
BnaA09.GH3-8.f     .D.ADG...........EATGE..........IVDLVDVKLGAYYEPLVTNYSGSLYRCR
BnaA09.GH3-8.g     .D.GD..............GE..........IVDLVNVKLGGYYEPLVTNYSG.LHRCR
BnaAX.GH3-8.h      .D...A...........GDKVE..........IVDLVDVKLGSHYEPLVTNHSG.LHRHK
BnaAX.GH3-8.i      .G.VDG...........GTEGE..........IVDLVDVKLGHYYEPLVTNYYG.LHRCR
BnaC01.GH3-8.a     .D...E...........GNDDA..........IVDLVDVKLGSYYELVVTTYSG.LHRCK
BnaC02.GH3-8.b     .D...A...........GDKVE..........IVDLVDVKLGSHYEPLVTNHSG.LHRHK
BnaC02.GH3-8.c     .D...A...........GDKVE..........IVDLVDVKLGSHYEPLVTNHSG.LHRHK
BnaC02.GH3-8.d     .D...A...........GDKVE..........IVDLVDVKLGSHYEPLVTNHSG.LHRHK
BnaC07.GH3-8.e     .G...E...........G.NDT..........IVDLVNVKLGRYYELVVTNYAG.LHRYR
BnaC09.GH3-8.f     .D...E...........GNKGE..........IVDLVHVEIGSYYEPLITNYYG.LHRYR
BnaCX.GH3-8.g      .D...G...........GTEGE..........IVDLVNVKLGHHYEPLVTNFSG.LHRCR
BnaA05.GH3-9.a     PC..................DK..........VVDLVDVKLGRYYELVVTTFAG.LYRYR
BnaC04.GH3-9.a     PC..................DK..........VVDLVDVKLGRYYELVVTTFAG.LYRYR
BnaA03.GH3-10.a    .HRQQN.QQDMTSDGDFVEEK...........PVPLSQVKLGQEYEVVLTTFTG.LYRYR
BnaA09.GH3-10.b    .YRQQTHQQDLCSEGDFVEEK...........PVPLSQVKLGQEYELVLTTFTG.LYRYR
BnaC09.GH3-10.a    .YRQQT.QQDLCSEGDFVEEK...........PVPLSQVKLGQEYELVLTTFTG.LYRYR
BnaA03.GH3-11.a    .SETGE...........TEERP..........PVGLTEVKVGQEYEVVITNYAG.LYRYR
BnaA04R.GH3-11.b   .S...E...........TEKE...........PVGLTDVKIGEEYEVVITNYAG.LYRYK
BnaA05.GH3-11.c    .SETGE...........GEQE...........PVGLTEVKVGQEYEVVLTNYAG.LYRYR
BnaC04.GH3-11.a    .SETEE...........TEEE...........PVCLTEVKIGQEYEVVITNYAG.LYRYK
BnaCX.GH3-11.b     .SETGD...........TEQEP..........PVGLTEVKIGQEYEVVITNYAG.LYRYR
BnaA02.GH3-12.a    .D...............GDKRD..........VVDLQDVKLGCTYEPVVTNFSG.LYRMR
BnaA03.GH3-12.b    .D...............GDKRD..........VVDLQDVKLGCTYEPVVTNFSG.LYRMR
BnaA06.GH3-12.c    .E...............EGSDD..........VVDLADVKLGCSYQLLITNLWG.LYRMR
BnaA10.GH3-12.d    .D...............GEKRD..........VVDLHDVKVGCTYEPVVTNFAG.LYRMR
BnaC02.GH3-12.a    .D...............GDKRD..........VVDLQDVKLGCTYEPVVTNFAG.LYRMR
BnaC03.GH3-12.b    .D...............GDKRD..........VVDLQDVKLGCTYEPVVTNFSG.LYRMR
BnaC09.GH3-12.c    .D...............GDKRD..........VVDLHDVKVGCTYEPVVTNFAG.LYRMR
BnaA10.GH3-14.a    .....K...........KD.QEAGHDPLVNPVVIDLVDVKVGHDYEPVVTTFSG.LYRYR
BnaC09.GH3-14.a    .....K...........KD.QEAGHDPLVNPVVVDLVDVKVGHDYEPVVTTFSG.LYRYR
BnaA03.GH3-15.a    .....E...........KDHQETSHDPTKN..IVDLVDVKVGHDYEPVITTFSG.LYRYR
BnaC03.GH3-15.a    .....E...........KDHQETSHDPTKN..IVDLVDVKVGHDYEPVITTFSG.LYRYR
BnaAX.GH3-16.a     .....E...........KDHQETGHNPTKNPVVVDLADVKIGHDYELIITTFSG.LYRYR
BnaC02.GH3-16.a    .....E...........KDHQETGHNPTKNPVVVDLADVKIGHDYELIITTFSG.LYRYR
BnaA07.GH3-17.a    DDDDDA...........LKEDL..........IVDLVNVEVGRYYEIVITTFTG.LYRYR
BnaA08.GH3-17.b    .....................R..........IVDLVSVEIGRYYELIITTFAG.LYRYR
BnaA09.GH3-17.c    DDDDDA...........LKEDL..........IVDLVNVEVGRYYEIVITTFTG.LYRYR
BnaC08.GH3-17.a    .....................R..........IVDLVNVEIGRYYELIITTFAG..QYR
BnaCX.GH3-17.b     DDDDDA...........LKEDL..........IVDLVNVEVGRYYEIVITTFTG.LYRYR
BnaCX.GH3-17.c     DDDDDA...........LKEDL..........IVDLVNVEVGRYYEIVITTFTG.LYRYR
BnaA05GH3-18.a     .D..HE...........GDMTS..........IVDLVNVKLGCYYEPVVTTCFG.LNRYL
BnaCX.GH3-18.a     .D.DDG...........GTTGE..........IVDLVNVKLGSYYELLVTNFSG.LHRCR
BnaA06.GH3-19.a    .DVDGG...........TTSGE..........VLDLVDVKLGRYYELLVTNFSG.LHRCR
BnaA09.GH3-19.b    .D.ADG...........EDTSE..........IVDLVDVTLGYYYEPLVTNYSGSLHRCR
consensus>70       ................................vdL.dvk.G..ye.vvt.y.g.l.r.r
```

```
                      410       420       430       440       450       460
AtGH3-1           VGDILRVTGFHNSAPQFHFVRRKNVLLSIDSDKTDESELQKAVENA.SSIL.HEECGSRV
AtGH3-2           VGDILRVTGFHNSAPQFKFIRRKNVLLSVESDKTDEAELQKAVENA.SRLF..AEQGTRV
AtGH3-3           VGDILQVTGFYNSAPQFKFVRRKNVLLSIESDKTDEAELQSAVENA.SLLL..GEQGTRV
AtGH3-4           VGDILRVTGFHNSAPQFKFIRRENVLLSIESDKTDEADLQKAVENA.SRLL..AEQGTRV
AtGH3-5           VGDLLRVTGFKNKAPQFSFICRKNVVLSIDSDKTDEVELQNAVKNA.VTHL..VPFDASL
AtGH3-6           VGDVLSVAGFKNNAPQFSFICRKNVVLSIDSDKTDEVELQNAVKNA.VTHL..VPFDASL
AtGH3-7           VGDILVVTGFHNKAPQFRFIRRDNVVLSIDLDKTNEDDLFKAVNNA.KLTL..DSSHLML
AtGH3-8           MGDILLVTGFYNNAPQFRFVRRGNLTLSIHLEITTDEDLLNAVTDA.KMVL..ESSNLML
AtGH3-9           IGDVLQVAGFYNGAPQFRFICRRNVVLSIDLDKTNEEDLHRSITLA.KKKL...GSNAFL
AtGH3-10          LGDVVEVTSFHKGTPKLSFIYRRKLILTINIDKNTEKDLQRVVDKA.SQLL.SRSTRAEV
AtGH3-11          LGDVVKVIGFYNNTPQLKFICRRNLILSINIDKNTERDLQLSVESA.AKRL..SEEKIEV
AtGH3-12          VGDIVLVTGFYNNAPQFKFVRRENVVLSIDSDKTNEEDLFKAVSQA.KLVL..ESSGLDL
AtGH3-13          VGDLLRVTGFYNNSPHFRFVGRQKVVLSLHMANTYEEDLLKAVTNA.KLLL..EPHDLML
AtGH3-14          LGDVLRVTGFYNNAPQFHFVGRQKVVLSIDMDKTYDEDLLKAVTNA.SLLL..EPHDLML
AtGH3-15          VGDVLRATGFYNNAPHFCFVGRQKVVLSIDMDKTYEDDLLKAVTNA.KLLL..EPHDLML
AtGH3-16          LGDVLRVTGFHNNAPQFYFVGRQNVVLSIDLSKTYEEDLLKAVKNA.SLLL..EPHDLML
AtGH3-17          VGDILKVTGFHNKAPQFRFVQRRNVVLSIDTDKTSEEDLLNAVTQA.KLNHLQHPSSLLL
AtGH3-18          IGDILQVTGFYNNTPQFRFVHRKNVVLSVRSETTTEEDILKALNHV.GLVL..ESSDLML
AtGH3-19          IGDILQVTGFYNNTPQFRFVRRKNIVLSVNSEATTEQDILKGLASA.TLVL..ESSNSML
BraX.GH3-1.a      VGDILRVTGFHNSAPQFHFVRRKNVLLSIDSDKTDESELQKAVENA.SKLL.LKECGTRV
BraX.GH3-2.a      VGDVLRVTGFHNSAPQFKFIRRKNVLLSIESDKTDEAELQKAVENA.SKLF..AAQGTRV
BraA09.GH3-3.a    VGDILQVTGFYNSAPQFKFVRRKNVLLSIESDKTDEAELQKAVENA.SVLL..EEQGTRV
BraA01.GH3-5.a    VGDLLKVTGFKNKAPQFSFICRKNVVLSIDSDKTDEVELQNAVKNA.VTHL..VPFDASV
BraA03.GH3-5.b    VGDLLRVTGFKNKAPQFSFICRKNVVLSIDSDKTDEVELQNAVKNA.MTHL..VPFDASL
BraA02.GH3-6.a    VGDVLRVAGFKNNAPQFSFICRKNVVLSIDSDKTDEVELQNAVKNA.VTHL..VPFDASL
BraA03.GH3-6.b    VGDVLRVAGFKNNAPQFSFICRKNVVLSIDSDKTDEVELQNAVKNA.VTHL..VPFDASL
BraA06.GH3-7.a    IGDIVKVTGFHNKAPKFRVIGRENTLLSIDTDRTTEEYLLKAVNRA.RLVL..ESSDLRL
BraA08.GH3-7.b    VGDILLVTGFYNKAPQLRFIRRDNVVLSIDMDKTNEEDLFNAVNRA.KLII..NSSGLML
BraA02.GH3-8.a    MGDVLQVTGFYNSAPQFRFVRRGNLVLSVHLEITTDEDLLNAVTHA.KMVL..ESSNLML
BraA03.GH3-8.b    MGDIVQVTGFYNNAPQFRFARRGNLVLSVHMEITTDQALLNAVTHA.KMVL..ESSNLML
BraA03.GH3-8.c    VGDVLQVTGFYNSAPQFKFIRRQNTVLSVYLEATTEEDLLKGVTRA.SQVL..KSSDIML
BraA03.GH3-8.d    VGDVLQVTGFYNSAPQFKFIRRQNVVLSVYLEATTEEDLLNAVTNA.AQLL..KSSDIML
BraA06.GH3-8.e    VGDVLQVTGFYNMAPQFRFVRRKNTVLSVYAEPTTEEDLLKALARA.TVIL..ESSDLMI
BraA06.GH3-8.f    VGDVLEVTGFYNMAPQLRFVRRKNAALSVHLESTTEEELLKALARA.TLVL..ESSDLVL
BraA09.GH3-8.g    MGDILQVTGFYNNAPQFRFVGRKKLVLSVNLEVTTEEDILKALNNA.TLVL..QRSDLLL
BraA09.GH3-8.h    VGDVLHVTGFYNNAPQFRFVRRKNTVLSIFVESTTEEDVLKALDRA.TVVL..ESSDLML
BraA10.GH3-8.i    MGDILQVSGFYNDAPQFRFVRRKNVVLSVSLEVTTEEDILKALNNA.TLVL..KSSDLVL
BraA09.GH3-10.a   LGDVVEVTGFHNGTPKLSFIYRRKLILTINIDKNTEKDLQRVVDKA.SQLL.SRTTHAEV
BraA03.GH3-11.a   LGDVVKIVGFYNKTPQLKFICRRNLILSINIDKNTERDLQMSVESA.AKRL..AEEKIEV
BraA04.GH3-11.b   LGDVVRIIGFYNKTPQLKFICRRNLILSINIDKNTERDLQLSVESA.AKRL..SEEKIEV
BraA05.GH3-11.c   LGDVVKIMSFYNKTPQLKFVCRRNLILSINIDKNTERDLQLSVESA.AKRL..SEEKIEV
BraA02.GH3-12.a   VGDVLLVTGFYNNAPQFKFVRRENVVLSIDSDKTNEEDLFKALSQA.KLVL..DSSDLTL
BraA03.GH3-12.b   VGDVLVVTGFYNNAPQFKFVRRENVVLSIDSDKTNEEDLFKALSQA.KLVL..ELSDLIL
BraA06.GH3-12.c   IGDMLKVTGFHNKAPQFRFMGRENTLLSIDTDRTNEKYLFKALNRA.TLVL..ESSDLRL
BraA10.GH3-12.d   VGDVLLVTGFYNNAPQFRFVRRENVVLSIDSDKTNEEDLFKAVSQA.KLVL..DSSDLFL
BraA10.GH3-14.a   LGDVLRVTGFYNNAPQFHFVGRNKVVLSIDMDKTYEEDLLNAVTNA.KLLL..EPHDFVL
BraA03.GH3-15.a   LGDVLRVTGFYNNAPEFQVAGRKKVVLSIDMDKTYEEDLLKAVTNA.KLLL..EPHDLML
BraA07.GH3-15.b   LGDVLRVTGFYNNAPQFRFVGRQKVVLSIDMDKTYEEDLLKAVANA.KLLL..EPHELML
BraA02.GH3-16.a   MGDVLRVTGFYNNAPQFYFVGRQKVVLSMDIDKTYEEDLHKAVTSA.THLL..EPHDLML
BraA07.GH3-17.a   VGDILKVTGFYNKAPQFRFVERRNVVLSIDTDKTSEEDLLNAVTQA.KLNHLQQPSSLLL
BraA09.GH3-17.b   VGDILKVTGFHNKAPQFRFVQRRNVVLSIDTDKTSEGDLLNAVTQA.KLNHLQKPSCPLL
BraAX.GH3-17.c    VGDILKVTGFHNKAPQFSFVERRNVVLSIDTDKTSEEDLMKAVTQA.KLNH..LQGLFLL
BraA04.GH3-19.a   ......VTGFYNKTPQFRFVRRKDTVISVHIEKTTEEDIVNAVNRV.TTVL..ESAGLML
BraA06.GH3-19.b   VGDVLEATGYYNKTPQFRFVRRKTTVLSVHLEPTTEEDLLKALARA.TVIL..ESLELML
BraA09.GH3-19.c   VGDVLQVTGFYNNTPQFRFVRRNNTVLCVDVEPTTEEDILKALARA.TVVL..ESSDFIL
BraA09.GH3-19.d   VGDVLQVTGFYNNTPQFRFVRRKDSVLCVDVEPTTEEDILKALARA.AVVL..ESSDLIL
BraAX.GH3-19.e    IGDILEVTGFYNNTPQFRFVRRKNMVLSVETETTTEDDILKALARA.TFVL..ESSNLML
BolC09.GH3-1.a    VGDILRVTGFHNSAPQFHFVRRKNVLLSIDSDKTDESELQKAVENA.SKLL.LKECGTRV
BolC01.GH3-2.a    VGDVLRVTGYHNSAPQFKFIRRKNVLLSIESDKTDEAELQKAVENA.SKLF..AAQGTRV
BolC08.GH3-3.a    VGDILQVTGFYNSAPQFKFVRRKNVLLSIESDKTDEAELQKAVENASSMLL..EERGTRV
BolC01.GH3-5.a    VGDLLKVTGFKNKAPQFSFICRKNVVLSIDSDKTDEVELQNAVKNA.VTHL..VPFDASV
BolC07.GH3-5.b    VGDLLRVTGFKNKAPQFSFICRKNVVLSIDSDKTDEVELQNAVKNA.MTHL..VPFDASL
BolC02.GH3-6.a    VGDVLRVAGFKNNAPQFSFICRKNVVLSIDSDKTDEVELQNAVKNA.VTHL..VPFDASL
BolC03.GH3-6.b    VGDVLRVAGFKNNAPQFNFICRKNVVLSIDSDKTDEVELQNAVKNA.VTHL..VPFDASL
BolC06.GH3-7.a    IGDMVKVTGFYNKAPQFRFLGRENALLSIDTDRTNEEYLFKAINRA.KLVL..ESSDLRL
BolC01.GH3-8.a    VGDVLQVTGFYNSAPQFKFVRRQNVVISVYLEATTEEELLKAVKRA.TEVI..ESSNIML
BolC03.GH3-8.b    ......VTGFYNSAPQFRFSRRGNLVLSVHMEITTDQVLLNAVTHG.KMVL..ESSDLML
BolC07.GH3-8.c    VGDVLQVTGFYNSVPQFKFIRRQNVVLSVYLEATTEEDLLKGVTHA.TQVL..KSSDIML
BolC08.GH3-8.d    VGDVLQVTGFYNKAPQFRSVRKKNTVLSIYVESTTEEDLLKALARA.AVVL..ESSDLIM
BolC09.GH3-8.e    MGDILQVSGFYNDAPQFRFVRRKNVALSVSLEVTTEEDILKALNNA.TLVL..KSSDLVL
BolC09.GH3-10.a   LGDVVEVTGFHHGTPKLSFIYRRKLILTINIDKNTEKDLQRVVDKA.SQLL.SRTTQAEV
BolC03.GH3-11.a   LGDVVKIVGFYNKTPQLKFICRRNLILSINIDKNTERDLQMSVESA.AKRL..AEEKIEV
BolC04.GH3-11.b   LGDVVKIIGFYNKTPQLKFICRRNLILSINIDKNTERDLQLSVESA.ARRL..SQEKIEV
BolCX.GH3-11.c    LGDVVKIMSFYNKTPQLKFVCRRNLILSINIDKNTERDLQLSVESA.AKRL..SEEKIEV
BolC03.GH3-12.a   VGDVLVVTGFYNNAPQFKFVRRENVVLSIDSDKTNEEDLFKALSQA.KLVL..ESSDLIL
BolC09.GH3-12.b   VGDVLLVTGFYNNAPQFRFVRRENVVLSIDSDKTNEEDLFKAVSQA.KLVL..DSSDLFL
BolC09.GH3-14.a   LGDVLRVTGFYNNAPQFHFVGRNKVVLSIDMDKTYEEDLLNAVTNA.KLLL..EPHDFVL
BolC03.GH3-15.a   LGDVLRVTGFYNNAPEFQVAGRKKVVLSIDMDKTYEEDLLKAVTNA.KLLL..EPHDLML
BolCX.GH3-16.a    LGDVLRVTGFYNNAPQFYFVGRQKVVLSMDIDKTYEEDLHKAVTSA.THLL..EPHDLML
BolC07.GH3-17.a   VGDILKVTGFYNKAPQFRFVERRNVVLSIDTDKTSEEDLLNAVTQA.KLNHLQQPSSLLL
BolC08.GH3-17.b   VGDILKVTGFHNKAPQFSFVERRNVVLSIDTDKTSEEDLMKAVTQA.KLIH..LQG.LLL
BolC08.GH3-19.a   VGDILQVTGFYNNTPQFRFVRRNNTVLCVDVEPTTEEDISKALARA.TVVL..ESSDLIL
BnaA09R.GH3-1.a   VGDILRVTGFHNSAPQFHFVRRKNVLLSIDSDKTDESELQKAVENA.SKLL.LKECGTRV
BnaCX.GH3-1.a     VGDILRVTGFHNSAPQFHFVRRKNVLLSIDSDKTDESELQKAVENA.SKLL.LKECGTRV
BnaA01.GH3-2.a    VGDVLRVTGFHNSAPQFKFIRRKNVLLSIESDKTDEAELQKAVENA.SKLF..AAQGTRV
BnaC01.GH3-2.a    VGDVLRVTGYHNSAPQFKFIRRKNVLLSIESDKTDEAELQKAVENA.SKLF..AAQGTRV
BnaA09.GH3-3.a    VGDILQVTGFYNSAPQFKFVRRKNVLLSIESDKTDEAELQKAVENA.SMLL..EEQGTRV
BnaC08.GH3-3.a    VGDILQVTGFYNSAPQFKFVRRKNVLLSIESDKTDEAELQKAVENASSMLL..EEQGTRV
BnaA01.GH3-5.a    VGDLLKVTGFKNKAPQFSFICRKNVVLSIDSDKTDEVELQNAVKNA.VTHL..VPFDASV
BnaA03.GH3-5.b    VGDLLRVTGFKNKAPQFSFICRKNVVLSIDSDKTDEVELQNAVKNA.MTHL..VPFDASL
BnaC01.GH3-5.a    VGDLLKVTGFKNKAPQFSFICRKNVVLSIDSDKTDEVELQNAVKNA.VTHL..VPFDASV
BnaC07.GH3-5.b    VGDLLRVTGFKNKAPQFSFICRKNVVLSIDSDKTDEVELQNAVKNA.MTHL..VPFDASL
BnaA03.GH3-6.a    VGDVLRVADFKNNAPQFSFICRKNVVLSIDSDKTDEVELQNAVKNA.VAHL..VPFDASL
BnaAX.GH3-6.b     VGDVLRVAGFKNNAPQFSFICRKNVVLSIDSDKTDEVELQNAVKNA.VTHL..VPFDASL
BnaC03.GH3-6.a    VGDVLRVAGFKNNAPQFNFICRKNVVLSIDSDKTDEVELQNAVKNA.VTHL..VPFDASL
BnaCX.GH3-6.b     VGDVLRVAGFKNNAPQFSFICRKNVVLSIDSDKTDEVELQNAVKNA.VTHL..VPFDASL
BnaA06.GH3-7.a    IGDIVKVTGFHNKAPKFRVIGRENTLLSIDTDRTTEEYLLKAVNRA.RLVL..ESSDLRL
BnaA08.GH3-7.b    VGDILLVTGFYNKAPQLRFIRRDNVVLSIDMDKTNEEDLFNAVNRA.KLII..NSSGLML
BnaC06.GH3-7.a    IGDMVKVTGFYNKAPQFRFLGRENALLSIDTDRTNEEYLFKAINRA.KLVL..ESSDLRL
BnaA03.GH3-8.a    ......VTGFYNNAPQFRFARRGNLVLSVHMEITTDQALLNAVTHA.KMVL..ESSNLML
BnaA03.GH3-8.b    VGDVLQVTGFYNSAPQLKFIRRQNTVLSVYLEATTEEDLLKGVTRA.SQVL..KSSDIML
BnaA03.GH3-8.c    VGDVLQVTGFYNSAPQFKFIRRQNVVLSVYLEATTEEDLLNAMTNA.AQLL..KSSDIML
BnaA06.GH3-8.d    VGDVLQVTGFYNMAPQFRFVRRKSTVLSVYAEPTTEEDLLKALARA.TVIL..ESSDLMI
```

```
BnaA06.GH3-8.e      ..............................MRSWSQ........................
BnaA09.GH3-8.f      VGDVLHVTGFYNNAPQFRFVRRKNTVLSIFVESTTEEDVLKALDRA.TVVL..ESSDLML
BnaA09.GH3-8.g      VGDVLQVTGFYNNTPQFRFARRKNTVLSVYVEPTTEEDLLNALTSA.TLAL..ESSDLIL
BnaAX.GH3-8.h       MGDVLQVTGFYNSAPQFRFVRRGNLVLSVHLEITTDEDLLNAVTHA.KMVL..ESSNLML
BnaAX.GH3-8.i       VGDVLQVTGFYNKAPQFRFVRRKNTVLSIYVESTTEEDLLKALA................
BnaC01.GH3-8.a      VGDVLQVTGFYNSAPQFKFVRRQNVVISVYLEATTEEELLKAVKRA.TEVI..ESSNIML
BnaC02.GH3-8.b      MGDVLQVTGFYNSAPQFRFVRRGNLVLSVHLEITTDEDLLNAVTHA.KMVL..ESSNLML
BnaC02.GH3-8.c      MGDVLQVTGFYNSAPQFRFVRRGNLVLSVHLEITTDEDLLNAVTHA.KMVL..ESSNLML
BnaC02.GH3-8.d      MGDVLQVTGFYNSAPQFRFVRRGNLVLSVHLEITTDEDLLNAVTHA.KMVL..ESSNLML
BnaC07.GH3-8.e      VGDVLQVTGFYNSVPQFKFIRRQNVVLSVYLEATTEEDLLKGVTHA.TQVL..KSSDIML
BnaC09.GH3-8.f      MGDILQVSGFYNNAPQFRFVGRKNLVLSVNLEVTTEEDILKALNNA.TLVL..QRSDLLL
BnaCX.GH3-8.g       VGDVLKVTGFYNMAPQFRFVRRKNAALSVHLESTTEEDLLKALARA.TLVL..ESSDLVI
BnaA05.GH3-9.a      IGDVLQVAGFYNKAPQFKFICRRNVVLSIDLDKTNEEDLHRSITLA.KKKL...ENKAFL
BnaC04.GH3-9.a      IGDVLQVAGFYNKAPQFRFICRRNVVLSIDLDKTNEEDLHRSITLA.KKKL...ENKAFL
BnaA03.GH3-10.a     LGDVVEVSGFHNGTPKISFIYRRKLILNINIDKNTEKDLQEVVDKA.SQLL.SKTTQAEV
BnaA09.GH3-10.b     LGDVVEVTGFHNGTPKLSFIYRRKLILTINIDKNTEKDLQRVVDKA.SQLL.SRTTHAEV
BnaC09.GH3-10.a     LGDVVEVTGFHHGTPKLSFIYRRKLILTINIDKNTEKDLQRVVDKA.SQLL.SRTTQAEV
BnaA03.GH3-11.a     LGDVVKIVGFYNKTPQLKFICRRNLILSINIDKNTERDLQMSVESA.AKRL..AEEKIEV
BnaA04R.GH3-11.b    LGDVVRIIGFYNKTPQLKFICRRNLILSINIDKNTERDLQLSVESA.AKRL..SEEKIEV
BnaA05.GH3-11.c     LGDVVKIMSFYNKTPQLKFVCRRNLILSINIDKNTERDLQLSVESA.AKRL..SEEKIEV
BnaC04.GH3-11.a     LGDVVKIIGFYNKTPQLKFICRRNLILSINIDKNTERDLQLSVESA.ARRL..SQEKIEV
BnaCX.GH3-11.b      LGDVVKIVGFYNKTPQLKFICRRNLILSINIDKNTERDLQMSVESA.AKRL..AEEKIEV
BnaA02.GH3-12.a     VGDVLLVTGFYNNAPQFKFVRRENVVLSIDSDKTNEEDLFKALSQA.KLVL..DSSDLTL
BnaA03.GH3-12.b     VGDVLVVTGFYNNAPQFKFVRRENVVLSIDSDKTNEEDLFKALSQA.KLVL..ELSDLIL
BnaA06.GH3-12.c     IGDMLKVTGFHNKAPQFRFMGRENTLLSIDTDRTNEKYLFKALNRA.TLVL..ESSDLRL
BnaA10.GH3-12.d     VGDVLLVTGFYNNAPQFRFVRRENVVLSIDSDKTNEEDLFKAVSQA.KLVL..DSSDLFL
BnaC02.GH3-12.a     VGDVLLVTGFYNNAPQFKFVRRENVVLSIDSDKTNEEDLFKALSQA.KLVL..DSSDLTL
BnaC03.GH3-12.b     VGDVLVVTGFYNNAPQFKFVRRENVVLSIDSDKTNEEDLFKALSQA.KLVL..ESSDLIL
BnaC09.GH3-12.c     VGDVLLVTGFYNNAPQFRFVRRENVVLSIDSDKTNEEDLFKAVSQA.KLVL..DSSDLFL
BnaA10.GH3-14.a     LGDVLRVTGFYNNAPQFHFVGRNKVVLSIDMDKTYEEDLLNAVTNA.KLLL..EPHDFVL
BnaC09.GH3-14.a     LGDVLRVTGFYNNAPQFHFVGRNKVVLSIDMDKTYEEDLLNAVTNA.KLLL..EPHDFVL
BnaA03.GH3-15.a     LGDVLRVTGFYNNAPEFQVAGRKKVVLSIDMDKTYEEDLLKAVTNA.KLLL..EPHDLML
BnaC03.GH3-15.a     LGDVLRVTGFYNNAPEFQVAGRKKVVLSIDMDKTYEEDLLKAVTNA.KLLL..EPHDLML
BnaAX.GH3-16.a      MGDVLRVTGFYNNAPQFYFVGRQKVVLSMDIDKTYEEDLHKAVTSA.THLL..EPHDLML
BnaC02.GH3-16.a     LGDVLRVTGFYNNAPQFYFVGRQKVVLSMDIDKTYEEDLHKAVTSA.THLL..EPHDLML
BnaA07.GH3-17.a     VGDILKVTGFHNKAPQFRFVQRRNVVLSIDTDKTSEEDLLNAVTQA.KLKHLQKPSCPLL
BnaA08.GH3-17.b     VGDILKVTGFHNKAPQFSFVERRNVVLSIDTDKTSEEDLMKAVTQA.KLNH..LQGLFLL
BnaA09.GH3-17.c     VGDILKVTGFHNKAPQFRFVQRRNVVLSIDTDKTSEGDLLNAVTQA.KLNHLQKPSCPLL
BnaC08.GH3-17.a     VGDILKVTGFHNKAPQFSFVERRNVVLSIDTDKTSEEDLMKAVTQA.KLIH..LQG.LLL
BnaCX.GH3-17.b      VGDILKVTGFHNKAPQFRFVQRRNVVLSIDTDKTSEEDLLNAVTQA.KLKHLQKPSCPLL
BnaCX.GH3-17.c      VGDILKVTGFHNKAPQFRFVQRRNVVLSIDTDKTSEEDLLNAVTQA.KLKHLQKPSCPLL
BnaA05GH3-18.a      IGDILEVTGFYNNTPQFRFVRRKNMVLSVETETTTEDDILKALARA.TFVL..ESSNLML
BnaCX.GH3-18.a      VGDVLQVTGFYNNTPQFRFVRRKNTVLCVDVEPTTEEDISKALARA.TVVL..ESSDLIL
BnaA06.GH3-19.a     VGDVLEATGYYNKTPQFRFVRRKTTVLSVHLEPTTEEDLLKALARA.TVIL..ESLDLML
BnaA09.GH3-19.b     VGDVLQVTGFYNNTPQFRFVRRNNTVLCVDVEPTTEEDILKALARA.TVVL..ESSDFIL
consensus>70        .gdvl.vtgf.n.apqf.fv.r.n.vlsid.d.t.#.dl..av..a....l.......l
```

```
                            470        480         490
                             .          .           .
AtGH3-1          AEYTS.......YADTSTI.P...GHYVLYWELLVR.....DGARQ........PSHETL
AtGH3-2          IEYTS.......YAETKTI.P...GHYVIYWELLGR.....DQSNAL.......MSEEVM
AtGH3-3          IEYTS.......YAETKTI.P...GHYVIYWELLVK.....DQTNP........PNDEVM
AtGH3-4          IEYTS.......YADTKTI.P...GHYVIYWELLSR.....DQSNAL.......PSDEVM
AtGH3-5          SEYTS.......YADTSSI.P...GHYVLFWELCLDGNTP.............IPPSVF
AtGH3-6          SEYTS.......YADTSSI.P...GHYVLFWELCLNGNTP.............IPPSVF
AtGH3-7          IDFTS.......YADISTI.P...GHYVVYWEVKNKN.....EDKKSKKHI..ELKEETF
AtGH3-8          MDFTS.......YADISTT.P...GHYVLYWELKAKY....RNDIVE.......IDKNVL
AtGH3-9          AEYTS.......YADTSSV.P...GHYVLFWEIQGH.................LEPKLM
AtGH3-10         VDFTS.......HADVIAR.P...GHYVIYWEIRGE.................ADDKAL
AtGH3-11         IDFSS.......YIDVSTD.P...GHYAIFWEISGE.................TNEDVL
AtGH3-12         KDFTS.......YADTSTF.P...GHYVVYLEVDTKE....GEEKET...AQFELDEEAL
AtGH3-13         MEFTS.......RVDSSSF.V...GHYVLYWELGSKV....KDAKLE.......PNRDVM
AtGH3-14         MDFTS.......RVDSSSY.P...GHYVLYWELGRKV....KDAKLE.......LDQNVL
AtGH3-15         MDFTS.......RVDSSSF.P...GHYVIYWELGSKV....KDAKFE.......PNRDVM
AtGH3-16         MDFTS.......RVDLSSL.P...GHYVLYWELGNKF....KNAKLD.......PKSNVL
AtGH3-17         TEYTS.......YADTSSI.P...GHYVLFWELKPRH....SNDPPK.......LDDKTM
AtGH3-18         MGFTC.......YADISTF.P...GHYVFYWELKAKD....VQDVFE.......LEEKVM
AtGH3-19         TGFTC.......YADISSF.P...GHYVFYWELKAKD....VDDVVE.......LDENVL
BraX.GH3-1.a     AEYTS.......YADTSTI.P...GHYVLYWELLVR.....EGERQ........PSHDTL
BraX.GH3-2.a     IEYTS.......YADTKTI.P...GHYVIYWEL..........TNAV.......PGDDVM
BraA09.GH3-3.a   IEYTS.......YADTKTI.P...GHYVIYWELLVK.....DQANP........PSDEVM
BraA01.GH3-5.a   SEYTS.......YADTSSI.P...GHYVLFWELCLDGNTP.............IPPSVF
BraA03.GH3-5.b   SEYTS.......YADTSSI.P...GHYVLFWELCLDGNTP.............IPPSVF
BraA02.GH3-6.a   SEYTS.......YADTSSI.P...GHYVLFWELCLNGNTP.............VPPSVY
BraA03.GH3-6.b   SEYTS.......YADTSSI.P...GHYVLFWELCLNGGTP.............VPPSVL
BraA06.GH3-7.a   VAFTS.......YADISSS.P...GHYVIYWEVKTKE.....EDMKE.......LDEKTF
BraA08.GH3-7.b   TDFTS.......HGDISTI.P...GHYVIYWEVEDKR.....EDKTK..HICMELKGDAF
BraA02.GH3-8.a   IDFTS.......YADVSTT.P...GHYVLYWELKGKY....NNDIAE.......IDNKVL
BraA03.GH3-8.b   MDFTG.......YADISTT.P...GHYVLYWELKGKY....SNDIAK.......IDDKVL
BraA03.GH3-8.c   RDFTC.......YPHVSDA.P...GHYVLYWELKGNN....DDGISE.......IDTNML
BraA03.GH3-8.d   KDFTC.......YPHISTV.P...GHYVLYWELKGNN....NDDINEL......IDTNVL
BraA06.GH3-8.e   TGFTC.......YADISTV.P...GHYVFYMELKAKV.NNTSIDDLV.......LDTKVL
BraA06.GH3-8.f   MGFTC.......YGDISTV.P...GHYVFYLELKSKV.DNNITNVLS.......LVNKVL
BraA09.GH3-8.g   MGFTS.......YADISTL.P...GHYIFYWELKAKN....ISDIVK.......PDNKVL
BraA09.GH3-8.h   TGFTC.......FGDVSTV.P...GHYVFYLELKAKV..NNDTNVLL.......LDEKVL
BraA10.GH3-8.i   MGFTC.......YADISTL.P...GHYIFYWELKSKT....ISDIVK.......LDNKVL
BraA09.GH3-10.a  VDFTS.......HADVKAR.P...GRYIIYWEIRGE.................AEDKAL
BraA03.GH3-11.a  IDFSS.......HVDVTTE.P...GHYVIYWEISGE.................TEDDVL
BraA04.GH3-11.b  IDFSS.......HVDVTTE.P...GHYVIFWEVSGE.................TEGDVL
BraA05.GH3-11.c  IDFSS.......HVDLSTE.P...GHYVIFWEISGE.................TNEDVL
BraA02.GH3-12.a  VDFTS.......YADTSTF.P...GHYVLFVEVKAKE....GNNRLE.......LNEEAF
BraA03.GH3-12.b  VDFTS.......YADTSTF.P...GHYVIYLEIKEKK....GENKKN....NVELSEEVF
BraA06.GH3-12.c  VAFTSYAHSATGYADTSSS.P...GHYVIYLEVKAKN.....EDIRD.....LEFDEKTF
BraA10.GH3-12.d  EDFTS.......YADTSTF.P...GHYVIYVEVKAKG....GNNHLE.......LNEEVF
BraA10.GH3-14.a  IDFTS.......RVDFSSY.P...GHYVLYWELGSKV....KDKKIE.......PERKVL
BraA03.GH3-15.a  IDFTS.......RVDSSSF.P...GHYVLYWELGSKV....NDAKLE.......PDAEVM
BraA07.GH3-15.b  IDFTS.......RVDSSSF.P...GHYVLYWELGCKV....KDVKVE.......PDPEVM
BraA02.GH3-16.a  IDFTS.......RVDSSSF.P...GHYVIYWELGRKL....KDAKVE.......PHRHVL
BraA07.GH3-17.a  TEYTS.......YADTSSI.P...GHYVLFWELKPRY....NNDPPK.......LDDKTM
BraA09.GH3-17.b  TEYTS.......YADTSSI.P...GHYVLFWELKPRH....NNEPPE.......LENKTM
BraAX.GH3-17.c   TEYTS.......YADTSSI.P...GHYVLFWELKPRY....DNDPPK.......LDKKMM
BraA04.GH3-19.a  MGFTC.......KSDMSTF.P...GHYVFYWEFKAKK....IDCIVK.......LDNNVM
BraA06.GH3-19.b  TGFTC.......YGDVSTV.P...GHYVFYLELKAKV.NNNSIDDEV.......LDNKVL
BraA09.GH3-19.c  TGFTC.......YGDISTV.P...GHYVFYLEVKAKV..NNGTNVLQ.......LDNKVL
BraA09.GH3-19.d  TGFTC.......YGDISTV.P...GHYVFYVELIAKV..NNGTNVLQ.......LDNKVL
BraAX.GH3-19.e   MDFTS.......SADISTF.P...GHYVFYWELKGKE....VNDVLV.......LDEKTL
BolC09.GH3-1.a   AEYTS.......YADTSTI.P...GHYVLYWELLVR.....DGERQ........PSHDTL
BolC01.GH3-2.a   IEYTS.......YADTRTI.P...GHYVIYWEL..........TNAV.......PGDDVM
BolC08.GH3-3.a   IEYTS.......YADTKTI.P...GHYVIYWELLVK.....DQANP........PSDEVM
BolC01.GH3-5.a   SEYTS.......YADTSSI.P...GHYVLFWELCLDGNTP.............IPPSVF
BolC07.GH3-5.b   SEYTS.......YADTSSI.P...GHYVLFWELCLDGNTP.............IPPSVF
BolC02.GH3-6.a   SEYTS.......YADTSSI.P...GHYVLFWELYLNGNTP.............VPPSVY
BolC03.GH3-6.b   SEYTS.......YADTSSI.P...GHYVLFWELCLNGGTP.............VPPSVL
BolC06.GH3-7.a   VDFTS.......YADISSSDP...GHYVIYWEVNVKN.....EDMKN.....LQFYKKTF
BolC01.GH3-8.a   RDFTC.......YPHIADT.P...GHYVLYWELKGNN....YDGISE.......LDPDMM
BolC03.GH3-8.b   MDFTG.......YADISTT.P...GHYVLYWELKGKY....SNDIAE.......IDDKVL
BolC07.GH3-8.c   RDFTC.......YPHVSDV.P...GHYVLYWELKGNN....EDGISE.......LNTNML
BolC08.GH3-8.d   TGFTC.......YADISTV.P...GHYVFYLELKAKV..NSGTNVLL.......LDNKVL
BolC09.GH3-8.e   MGFTC.......YADISTL.PGDSGHEVYT.......................LSHNLL
BolC09.GH3-10.a  VDFTS.......HADVTAR.P...GRYIIYWEIRGE.................AEDKAL
BolC03.GH3-11.a  IDFSS.......HVDVSTE.P...GHYVIYWEISGE.................TEDDVL
BolC04.GH3-11.b  IDFSS.......HVDVTTE.P...GHYVIFWEISGE.................TEGDVL
BolCX.GH3-11.c   IDFSS.......HVDLSTE.P...GHYVIFWEISGE.................TNEDVL
BolC03.GH3-12.a  VDFTS.......YADTSTF.P...GHYVIYLEIKEKE....GENKKN....NVELSEEVF
BolC09.GH3-12.b  EDFTS.......YADTSTF.P...GHYVLYVEVRAKE....GKKYLELNKEALDLNKEAF
BolC09.GH3-14.a  IDFTS.......RVDFSSY.P...GRYVLYWELGSKV....KDKKLE.......PKREVM
BolC03.GH3-15.a  IDFTS.......RVDSSSF.P...GHYVLYWELGSKV....KDAKLE.......PDAEVM
BolCX.GH3-16.a   IDFTS.......RVDSSSF.P...GHYVIYWELGRKL....KDAKVE.......PHRHVL
BolC07.GH3-17.a  TEYTS.......YADTSSI.P...GHYVLFWELKPRY....NNDPPK.......LDDKTM
BolC08.GH3-17.b  TEYTS.......YADTSSI.P...GHYVLFWELKPRY....DNDPPM.......LDKKMM
BolC08.GH3-19.a  TGFTC.......YGDISTV.P...GHYVFYVELKAKV..KNGTNVLQ.......LDNKVL
BnaA09R.GH3-1.a  AEYTS.......YADTSTI.P...GHYVLYWELLVR.....DGERQ........PSHDTL
BnaCX.GH3-1.a    AEYTS.......YADTSTI.P...GHYVLYWELLVR.....DGERQ........PSHDTL
BnaA01.GH3-2.a   IEYTS.......YADTKTI.P...GHYVIYWEL..........TNAV.......PGDDVM
BnaC01.GH3-2.a   IEYTS.......YADTRTI.P...GHYVIYWEL..........TNAV.......PGDDVM
BnaA09.GH3-3.a   IEYTS.......YADTKTI.P...GHYVIYWELLVK.....DQANP........PSDEVM
BnaC08.GH3-3.a   IEYTS.......YADTKTI.P...GHYVIYWELLVK.....DQANP........PSDEVM
BnaA01.GH3-5.a   SEYTS.......YADTSSI.P...GHYVLFWELCLDGNTP.............IPPSVF
BnaA03.GH3-5.b   SEYTS.......YADTSSI.P...GHYVLFWELCLDGNTP.............IPPSVF
BnaC01.GH3-5.a   SEYTS.......YADTSSI.P...GHYVLFWELCLDGNTP.............IPPSVF
BnaC07.GH3-5.b   SEYTS.......YADTSSI.P...GHYVLFWELCLDGNTP.............IPPSVF
BnaA03.GH3-6.a   SEYTS.......YADTSSI.P...GHYVLFWELCLNGDTP.............VPPSVL
BnaAX.GH3-6.b    SEYTS.......YADTSSI.P...GHYVLFWELCLNGNTP.............VPPSVY
BnaC03.GH3-6.a   SEYTS.......YADTSSI.P...GHYVLFWELCLNGGTP.............VPPSVL
BnaCX.GH3-6.b    SEYTS.......YADTSSI.P...GHYVLFWELYLNGNTP.............VPPSVY
BnaA06.GH3-7.a   VAFTS.......YADISSS.P...GHYVIYWEVKTKE.....EDMKE.......LDEKTS
BnaA08.GH3-7.b   TDFTS.......HGDISTI.P...GHYVIYWEVEDKR.....EDKTK..HICMELKGDAF
BnaC06.GH3-7.a   VDFTS.......YADISSSDP...GHYVIYWEVNVKN.....EDMKN.....LQFYKKTF
BnaA03.GH3-8.a   MDFTG.......YADISTT.P...GHYVLYWELKGKY....SNDIAK.......IDDK..
BnaA03.GH3-8.b   RDFTC.......YPHVSDA.P...GHYVLYWELKGNN....DDGISE.......IDTNML
BnaA03.GH3-8.c   KDFTC.......YPHISTV.P...GHYVLYWELKGNI....NDDINEL......IETNVL
BnaA06.GH3-8.d   TGFTC.......YADISTV.P...GHYVFYMELKAKV.NNTSIDDLV.......LDTKVL
```

```
BnaA06.GH3-8.e       .................TF.P...LIFFFLME...........................
BnaA09.GH3-8.f       TGFTC.......FGDVSTV.P...GHYVFYLELKAKV..NNDTNVLL.......LDEKVL
BnaA09.GH3-8.g       TGFTC.......YADVSTV.P...GHYVFYLELKAKV..NNSTGELP.......LDNNI.
BnaAX.GH3-8.h        IDFTS.......YADVSTT.P...GHYVLYWELKGKY....NNDIAE.......IDNKVL
BnaAX.GH3-8.i        ..................................PKV..NSGTNVLL.......LDNKVL
BnaC01.GH3-8.a       RDFTC.......YPHIADT.P...GHYVLYWELKGNN....YDGISE.......LDPDMM
BnaC02.GH3-8.b       IDFTS.......YADVSTT.P...GHYVLYWELKGKY....NNDIAE.......IDNKVL
BnaC02.GH3-8.c       IDFTS.......YADVSTT.P...GHYVLYWELKGKY....NNDIAE.......IDNKVL
BnaC02.GH3-8.d       IDFTS.......YADVSTT.P...GHYVLYWELKGKY....NNDIAE.......IDNKVL
BnaC07.GH3-8.e       RDFTC.......YPHVSDV.P...GHYVLYWELKGNN....EDGISE.......LNTNML
BnaC09.GH3-8.f       MGFTS.......YADISTL.P...GHYIFYWELKAKN....ISDTIK.......PDNKVL
BnaCX.GH3-8.g        MGFTC.......YADISTV.P...GHYVFYLELKSKVNNNNSTNVLS.......LDNKVL
BnaA05.GH3-9.a       AEYTS.......YADTSSV.P...GHYVLFWEIQ.W.....LEPDDE.......EEKLLM
BnaC04.GH3-9.a       AEYTS.......YADTSSV.P...GHYVLFWEIQ.W.....LEPDDE.......EEKLLM
BnaA03.GH3-10.a      VDFTS.......HADVKVS.P...GRYIIYWEIKGE..................AEDRAL
BnaA09.GH3-10.b      VDFTS.......HADVKAR.P...GRYIIYWEIRGE..................AEDKAL
BnaC09.GH3-10.a      VDFTS.......HADVTAR.P...GRYIIYWEIRGE..................AEDKAL
BnaA03.GH3-11.a      IDFSS.......HVDVKTE.P...GHYVIYWEISGE..................TEDDVL
BnaA04R.GH3-11.b     IDFSS.......HVDVTTE.P...GHYVIFWEVSGE..................TEEDVL
BnaA05.GH3-11.c      IDFSS.......HVDLSTE.P...GHYVIFWEISGE..................TNEDVL
BnaC04.GH3-11.a      IDFSS.......HVDVTTE.P...GHYVIFWEISGE..................TEGDVL
BnaCX.GH3-11.b       IDFSS.......HVDVSTE.P...GHYVIYWEISGE..................TGDDVL
BnaA02.GH3-12.a      VDFTS.......YADTSTF.P...GHYVLYVEVKAKE....GNSHLE.......LNEEAF
BnaA03.GH3-12.b      VDFTS.......YADTSTF.P...GHYVIYLEKKEKK....GENKKN....NVELSEEVF
BnaA06.GH3-12.c      VAFTS.......YADTSSS.P...GHYVIYLEVKAKN.....EDIRD.....LEFDEKTF
BnaA10.GH3-12.d      EDFTS.......YADTSTF.P...GHYVLYVEVKAKG....GNNHLE.......LNEEVF
BnaC02.GH3-12.a      VDFTS.......YADTSTF.P...GHYVLYVEVKAKE....GNNHLE.......LNEEAF
BnaC03.GH3-12.b      VDFTS.......YADTSTF.P...GHYVIYLEIKEKE....GENKKN....NVELSEEVF
BnaC09.GH3-12.c      EDFTS.......YADTSTF.P...GHYVLYVEVRAKE....GKKYLELNKEALDLNKEAF
BnaA10.GH3-14.a      IDFTS.......RVDFSSY.P...GHYVLYWELGSKV....KDKKIE.......PERKVL
BnaC09.GH3-14.a      IDFTS.......RVDFSSY.P...GRYVLYWELGSKV....KDKKLE.......PKREVM
BnaA03.GH3-15.a      IDFTS.......RVDSSSF.P...GHYVLYWELGSKV....KDAKLE.......PDAEVM
BnaC03.GH3-15.a      IDFTS.......RVDSSSF.P...GHYVLYWELG..........................
BnaAX.GH3-16.a       IDFTS.......RVDSSSF.P...GHYVIYWELGRKL....KDAKVE.......PHRHVL
BnaC02.GH3-16.a      IDFTS.......RVDSSSF.P...GHYVIYWELGRKL....KDAKVE.......PHRHVL
BnaA07.GH3-17.a      TEYTS.......YADTSSI.P...GHYVLFWELKPRY....NNDPPK.......LDDKTM
BnaA08.GH3-17.b      TEYTS.......YADTSSI.P...GHYVLFWELKPRY....DNDPPK.......LDKKMM
BnaA09.GH3-17.c      TEYTS.......YADTSSI.P...GHYVLFWELKPRH....NNEPPE.......LENKTM
BnaC08.GH3-17.a      TEYTS.......YADTSSI.P...GHYVLFWELKPRY....DNDPPM.......LDKKMM
BnaCX.GH3-17.b       TEYTS.......YADTSSI.P...GHYVLFWELKPRQ....NNEPPE.......LENKTM
BnaCX.GH3-17.c       TEYTS.......YADTSSI.P...GHYVLFWELKPRQ....NNEPPE.......LENKTM
BnaA05GH3-18.a       MDFTS.......SADISTF.P...GHYVFYWELKGKE....VNDVLV.......LDEKTL
BnaCX.GH3-18.a       TGFTC.......FGDISTV.P...GHYVFYVELKATV..NSGTTVLQ.......LDNKVL
BnaA06.GH3-19.a      TGFTC.......YGDVSTV.P...GHYVFYLELKSKD..NKSIDNVV.......LDNKVL
BnaA09.GH3-19.b      TGFTC.......YGDISTV.P...GHYVFYLEVKAKV..NNGTNVLQ.......LDNKVL
consensus>70         .dfts.......y.d.s...p...ghyv.ywe...........................l
```

```
                                500       510       520                 530       540
AtGH3-1          .......TRCCLGMEESL.NSVYRQSRVADNSVG.........PLEIRVVRNGTFEELMD
AtGH3-2          .......AKCCLEMEESL.NSVYRQSRVADKSIG.........PLEIRVVRNGTFEELMD
AtGH3-3          .......ARCCLEMEESL.NSVYRQSRVADKSIG.........PLEIRVVKNGTFEELMD
AtGH3-4          .......AKCCLEMEESL.NAVYRQSRVSDKSIG.........PLEIRVVQNGTFEELMD
AtGH3-5          .......EDCCLAVEESF.NTVYRQGRVSDKSIG.........PLEIKIVEPGTFDKLMD
AtGH3-6          .......EDCCLTIEESL.NSVYRQGRVSDKSIG.........PLEIKMVESGTFDKLMD
AtGH3-7          .......SECCLLMEDSL.DSVYKICRFKEESVG.........PLEIKVVRQGTFDSLMD
AtGH3-8          .......VECCYVVEESL.NNSYRVYRSKGGLIG.........ALEIRLVQQGTFDALME
AtGH3-9          .......EECCVAVEEEL.DYIYRQCRTKERSIG.........ALEIRVVKPGTFEKLMD
AtGH3-10         .......EECCREMDTAFVDYGYVVSR.RMNSIG.........PLELRVVERGTFGKVAE
AtGH3-11         .......QDCCNCLDRAFIDAGYVSSR.KCKTIG.........ALELRVVAKGTFRKIQE
AtGH3-12         .......STCCLVMEESL.DNVYKRCRFKDGSIG.........PLEIRVVRQGTFDSLMD
AtGH3-13         .......EECCFTVEKYL.DPLYRQERRKDKNIG.........PLEIKVVKPGAFDELMN
AtGH3-14         .......EECCFTIEESL.DAVYRKGRKNDKNIG.........PLEIKVVKPGAFDELMN
AtGH3-15         .......EECCFTVEESL.DAVYRKGRKNDKNIG.........PLEIKVVKPGAFDELMN
AtGH3-16         .......EECCLTVEESL.DSIYREGRKNDKIIG.........PLEIKVVKPGAFDELMN
AtGH3-17         .......EDCCSEVEDCL.DYVYRRCRNRDKSIG.........PLEIRVVSLGTFDSLMD
AtGH3-18         .......VKCCSLLEESF.DEVYRKNRSKDECIG.........PLEIRVVQQGTFDSLME
AtGH3-19         .......EECCYALEESF.DALYKRLRSKEGSIG.........ALEIKVVQQGTFDSLME
BraX.GH3-1.a     .......TRCCLEMEESL.NSVYRQCRVADNSVG.........PLEIRVVRNGTFEELMD
BraX.GH3-2.a     .......AKCCLEMEESL.NSVYRQSRVADKSIG.........PLEIRVVQSGTFEELMD
BraA09.GH3-3.a   .......AKCCLEMEESL.NSVYRQSRVADKSIG.........PLEIRVVKNGTFEELMD
BraA01.GH3-5.a   .......EDCCLALEESL.NTVYRQGRVSDKSIG.........PLEIKIVEPGTFDKLMD
BraA03.GH3-5.b   .......EDCCLAVEESF.NSVYRQGRVSDKSIG.........PLEIKIVEPGTFDKLMD
BraA02.GH3-6.a   .......EDCCLTIEESF.NSVYRQGRVSDKSIG.........PLEIKVVESGTFDKLMD
BraA03.GH3-6.b   .......EDCCLTIEESL.NSVYRQGRVSDKSIG.........PLEIKVVESGTFDKLMD
BraA06.GH3-7.a   .......LECCSVMEDTL.DEEYMYCR.ANEFIG.........PLEIRVVNDGTFDSLMN
BraA08.GH3-7.b   .......SECCLVMEDSF.DNVYKRCRFREKTVG.........PLEIKVVLHGTFDSLMD
BraA02.GH3-8.a   .......VECCYVVEESL.NNFYKEFRSKDGSIG.........ALEIRVVQQGTFDSLME
BraA03.GH3-8.b   .......VECCYVVEESL.DNFYKEYR.KNGSIG.........ALEIRVVQQGTFDSLME
BraA03.GH3-8.c   .......VECCSVVEESL.GALYKRYRSKERSIG.........ALEIRVVQQGTFDALME
BraA03.GH3-8.d   .......MECCSVVEESL.DALYRKFRSKDESIG.........ALEIRVVQQGTFDSLME
BraA06.GH3-8.e   .......VECCCVMEESL.NGSYRSFRRKNGWIG.........ALEIRVVQQGTFASLMD
BraA06.GH3-8.f   .......VECCCVIEESL.NRIYRHFRSKEGSIG.........ALEIRVVQEGTFGSLMD
BraA09.GH3-8.g   .......VECCCVMEESL.SALYREIRSKDGSIG.........PLEIRIVQQGTFNSLME
BraA09.GH3-8.h   .......VEYCCVMEESL.SSFYRSLRRKDGPIG.........PLEVRVVQQGTFDSLME
BraA10.GH3-8.i   .......VECCCVMEESL.CALYRELRSNDGSIG.........ALEIRVVQQETFNSLME
BraA09.GH3-10.a  .......EDCCREMDAGFVDHGYVVSR.RMKSIG.........PLELRVVGRGTFGKVAE
BraA03.GH3-11.a  .......QDCCNCLDKGFIDAGYMSSR.KCKTIG.........ALELRVVERGTFRKVQE
BraA04.GH3-11.b  .......QDCCNCLDKGFIDAGYMSSR.KCKTIG.........ALELRVVERGTFRKVQE
BraA05.GH3-11.c  .......QDCCNCLDKGFIDAGYMSSR.KCKTIG.........ALELRVLEKGTFKKIQE
BraA02.GH3-12.a  .......SKCCSVIEDSL.DNVYKRCRFKDGSVG.........PLEIRVVPQGTFDSLMD
BraA03.GH3-12.b  .......SKCCSVMEDSL.DNVYKRCRFKDGSVG.........PLEIRVVRQGMFDSLMD
BraA06.GH3-12.c  .......LECCLVMEDSF.DDEYRYCR.SNEFVG.........PLEIRVVNDGSFDSLMN
BraA10.GH3-12.d  .......SKCCSVMEDSL.DNVYKRCRFKDGSVG.........PLEIRVVDKGTFESLMD
BraA10.GH3-14.a  .......EECCFTVEESL.DSVYRKGRKNDKNIG.........PLEIKVVRRGAFDELMS
BraA03.GH3-15.a  .......EECCFTMEESL.DSIYRKGRKNDKNIG.........PLEIKVVKSGAFDELMN
BraA07.GH3-15.b  EECCFAVEECCFAVEECL.DSVYRNGRRNDKNIG.........PLEIKVVRSGAFDELMS
BraA02.GH3-16.a  .......EECCFTIEKSL.NSVYRKLRKNDGTVG.........PLEIKVVRRGAFDELMN
BraA07.GH3-17.a  .......EKCCSEVEDRL.DYVYRRCRNKDKSIG.........PLEIRVVSLGTFDLLMD
BraA09.GH3-17.b  .......ELCCSEVEDYL.DYVYRRCRNKDKSIG.........PLEIRVVSLGTFDLLMD
BraAX.GH3-17.c   .......EDCCSEVEDCL.DYVYRRCRNKDRSIG.........ALEIRVVSLGTFDALMD
BraA04.GH3-19.a  .......VKCCCVMEESF.NALYRRHRRKYGTIG.........PLEIRVMQQGTFDSLME
BraA06.GH3-19.b  .......VECCRVMEESL.NGTYRRFRRKNGSIG.........ALEIRVVQQGTFDSLMD
BraA09.GH3-19.c  .......VECCCIMEESL.SSLYRRLRGDEGSIG.........ALEIRVVQQGTFDSLME
BraA09.GH3-19.d  .......VEYCCVMEESL.SSLYRRLRVKDGPIG.........PLEVRVVEQGTFDSLME
BraAX.GH3-19.e   .......MGCCYVLEESF.GSTYRLKR.RTGLIG.........ALEIRVVEQGTFNSLTD
BolC09.GH3-1.a   .......TRCCLEMEESL.NSVYRQCRVADNSVG.........PLEIRVVRNGTFEELMD
BolC01.GH3-2.a   .......AKCCLEMEESL.NSVYRQSRVADKSIG.........PLEIRVVQSGTFEELMD
BolC08.GH3-3.a   .......AKCCLEMEESL.NSVYRQSRVADNSIG.........PLEIRVVKNGTFEELMD
BolC01.GH3-5.a   .......EDCCLALEESL.NTVYRQGRVSDKSIG.........PLEIKIVGAGTFDKLMD
BolC07.GH3-5.b   .......EDCCLAVEESF.NSVYRQGRVSDKSIG.........PLEIKIVEPGTFDKLMD
BolC02.GH3-6.a   .......EDCCLTIEESF.NSVYRQGRVSDKSIG.........PLEIKVVESGTFDKLMD
BolC03.GH3-6.b   .......EDCCLTIEESL.NSVYRQGRVSDKSIG.........PLEIKVVESGTFDKLMD
BolC06.GH3-7.a   .......LECCSVMEDSF.DDEYRYCR.SNEFVG.........PLEIRVVNDGTFDSLMN
BolC01.GH3-8.a   .......VECCSVIEESL.NALYRKFRSKEKTIG.........ALEIRVVQPGTFDSLME
BolC03.GH3-8.b   .......V..........................................................
BolC07.GH3-8.c   .......VECCSVVEESL.GALYKKYRSKEGSIG.........PLEIRVVQQGSFDALME
BolC08.GH3-8.d   .......VEYCCVMEESL.SSSYRTLRRQDGLIG.........ALEVRIVQKETFDSLME
BolC09.GH3-8.e   ...........LAHAEAV.EEFRKCAKCKDGNIGIVRSPMWFEPYEKKSSSEEIVKRALD
BolC09.GH3-10.a  .......EDCCREMDAGFVDHGYVVSR.RMKSIG.........PLELRVVERGTFGEVAE
BolC03.GH3-11.a  .......QDCCNCLDKGFIDAGYMSSR.KCKTIG.........ALELRVVERGTFRKVQE
BolC04.GH3-11.b  .......QDCCNCLDKGFIDAGYMSSR.KCETIG.........ALELRVVERGTFRKVQE
BolCX.GH3-11.c   .......QDCCNCLDKGFIDAGYMSSR.KCKTIG.........ALELRVLEKGTFKKIQE
BolC03.GH3-12.a  .......PKCCSVMEDSL.DNVYKRCRFKDGSVG.........PLEIRVVRQGMFDSLMD
BolC09.GH3-12.b  .......SKCCSVMEDSL.DNVYKRCRFKDGSVG.........PLEIRVVGKGTFDSLMN
BolC09.GH3-14.a  .......EECCFTVEESL.DSVYRKGRKNDKNIG.........PLEIKVVRRGAFDELMN
BolC03.GH3-15.a  .......EECCFTMEESL.DSIYRKGRKNDKNIG.........PLEIKVVKSGAFDELMN
BolCX.GH3-16.a   .......EECCFTIEKSL.NSVYRKLRMNDGTVG.........PLEIKVVKRGAFDELMN
BolC07.GH3-17.a  .......EKCCSEVEDRL.DYVYRRCRNKDKSIG.........PLEIRVVSLGTFDLLMD
BolC08.GH3-17.b  .......EDCCSEVEDCL.DYVYRRCRNKDGSIG.........ALEMRVVSLGTFDALMD
BolC08.GH3-19.a  .......VEYCCVMEESL.SGIYRRLRGDEGSIG.........PLEVRIVQQGTFDSLME
BnaA09R.GH3-1.a  .......TRCCLEMEESL.NSVYRQCRVADNSVG.........PLEIRVVRNGTFEELMD
BnaCX.GH3-1.a    .......TRCCLEMEESL.NSVYRQCRVADNSVG.........PLEIRVVRNGTFEELMD
BnaA01.GH3-2.a   .......AKCCLEMEESL.NSVYRQSRVADKSIG.........PLEIRVVQSGTFEELMD
BnaC01.GH3-2.a   .......AKCCLEMEESL.NSVYRQSRVADKSIG.........PLEIRVVQSGTFEELMD
BnaA09.GH3-3.a   .......AKCCLEMEESL.NSVYRQSRVADKSIG.........PLEIRVVKNGTFEELMD
BnaC08.GH3-3.a   .......AKCCLEMEESL.NSVYRQSRVADKSIG.........PLEIRVVKNGTFEELMD
BnaA01.GH3-5.a   .......EDCCLALEESL.NTVYRQGRVSDKSIG.........PLEIKIVEPGTFDKLMD
BnaA03.GH3-5.b   .......EDCCLAVEESF.NSVYRQGRVSDKSIG.........PLEIKIVEPGTFDKLMD
BnaC01.GH3-5.a   .......EDCCLALEESL.NTVYRQGRVSDKSIG.........PLEIKIVEAGTFDKLMD
BnaC07.GH3-5.b   .......EDCCLAVEESF.NSVYRQGRVSDKSIG.........PLEIKIVEPGTFDKLMD
BnaA03.GH3-6.a   .......EDCCLTIEESL.NSVYRQGRVSDKSIG.........PLEIKVVESGTFDKLMD
BnaAX.GH3-6.b    .......EDCCLTIEESF.NSVYRQGRVSDKSIG.........PLEIKVVESGTFDKLMD
BnaC03.GH3-6.a   .......EDCCLTIEESL.NSVYRQGRVSDKSIG.........PLEIKVVESGTFDKLMD
BnaCX.GH3-6.b    .......EDCCLTIEESF.NSVYRQGRVSDKSIG.........PLEIKVVESGTFDKLMD
BnaA06.GH3-7.a   .......LECCSVMEDTL.DEEYMYCR.ANEFIG.........PLEIRVVNDGTFDSLMN
BnaA08.GH3-7.b   .......SECCLVMEDSF.DNVYKRCRFREKTVG.........PLEIKVVLHGTFDSLMD
BnaC06.GH3-7.a   .......LECCSVMEDSF.DDEYRYCR.SNEFVG.........PLEIRVVNDGTFDSLMN
BnaA03.GH3-8.a   ...............ESL.DNFYKEYR.KNGSIG.........ALEIRVVQQGTFDSLME
BnaA03.GH3-8.b   .......VDCCFVVEESL.GALYKRYRSKERSIG.........ALEIRVVQQGTFDALME
BnaA03.GH3-8.c   .......MECCSVVEESL.DALYRKFRSKDESIG.........ALEIRVVQQGTFDSLME
BnaA06.GH3-8.d   .......VECCCVMEESL.NGSYRSFRRKNGWIG.........ALEIRVVQQGTFDSLMD
```

```
BnaA06.GH3-8.e      ...............................................RLVLSWT......
BnaA09.GH3-8.f      .......VEYCCVMEESL.SSFYRSLRRKDGPIG.........PLEVRVVQQGTFDSLME
BnaA09.GH3-8.g      ......................YRHFRAKDGFIG.........ALEIRVVEQGTFDSLME
BnaAX.GH3-8.h       .......VECCYVVEESL.NNFYKEFRSKDGSIG.........ALEIRVVQQGTFDSLME
BnaAX.GH3-8.i       .......VEYCCVMEESL.SSSYRTLRRQDGLIG.........ALEVRIVQKGTFDSLME
BnaC01.GH3-8.a      .......VECCSVIEESL.NALYRKFRSKEKTIG.........ALEIRVVQPGTFDSLME
BnaC02.GH3-8.b      .......VECCYVVEESL.NNFYKEFRSKDGSVG.........ALEIRVVQQGTFESLME
BnaC02.GH3-8.c      .......VECCYVVEESL.NNFYKEFRSKDGSVG.........ALEIRVVQQGTFESLME
BnaC02.GH3-8.d      .......VECCYVVEESL.NNFYKEFRSKDGSVG.........ALEIRVVQQGTFESLME
BnaC07.GH3-8.e      .......VECCSVVEESL.GALYKKYRSKEGSIG.........PLEIRVVQQGSFDALME
BnaC09.GH3-8.f      .......VECCCVMEESL.STLYREIRSKDGSIG.........PLEIRVVQQGTFNSLME
BnaCX.GH3-8.g       .......VECCCVMEESL.NSFYRLFRSKNGSIG.........APEIRVVQEGTFGSLME
BnaA05.GH3-9.a      .......EECCIAVEEEL.DYIYRQCRKRDRSVG.........PLEIRVVKAGTFEKLMD
BnaC04.GH3-9.a      .......EECCIAVEEEL.DYIYRQCRKRDRSVG.........PLEIRVVKPGTFEKLMD
BnaA03.GH3-10.a     .......EECCREIDAGFMDHGYVV...................................
BnaA09.GH3-10.b     .......EDCCREMDAGFVDHGYVVSR.RMKSIG.........PLELRVVERGTFGKVAE
BnaC09.GH3-10.a     .......EDCCREMDAGFVDHGYVVSR.RMKSIG.........PLELRVVERGTFGEVAE
BnaA03.GH3-11.a     .......QDCCNCLDKGFIDAGYMSSR.KCKTIG.........ALELRVVERGTFRKVQE
BnaA04R.GH3-11.b    .......QDCCNCLDKGFIDAGYMSSR.KCKTIG.........ALELRVVERGTFRKVQE
BnaA05.GH3-11.c     .......QDCCNCLDKGFIDAGYMSSR.KCKTIG.........ALELRVLEKGTFKKIQE
BnaC04.GH3-11.a     .......QDCCNCLDKGFIDAGYMSSR.KCETIG.........ALELRVVERGTFRKVQE
BnaCX.GH3-11.b      .......QDCCNCLDKGFIDAGYMSSR.KCKTIG.........ALELRVVERGTFRKVQE
BnaA02.GH3-12.a     .......SKCCSVIEDSL.DNVYKRCRFKDGSVG.........PLEIRVVPQGTFDSLMD
BnaA03.GH3-12.b     .......SKCCSVMEDSL.DNVYKRCRFKDGSVG.........PLEIRVVRQGMFDSLMD
BnaA06.GH3-12.c     .......LECCLVMEDSF.DDEYRYCR.SNEFVG.........PLEIRVVNDGSFDSLMN
BnaA10.GH3-12.d     .......SKCCSVMEDSL.DNVYKRCRFKDGSVG.........PLEIRVVDKGTFESLMD
BnaC02.GH3-12.a     .......SKCCSVIEDSL.DNVYKRCRFKDGSVG.........PLEIRVVRQGTFDSLMD
BnaC03.GH3-12.b     .......SKCCSVMEDSL.DNVYKRCRFKDGSVG.........PLEIRVVRQGMFDSLMD
BnaC09.GH3-12.c     .......SKCCSVMEDSL.DNVYKRCRFKDGSVG.........PLEIRVVGKGTFDSLMN
BnaA10.GH3-14.a     .......EECCFTVEESL.DSVYRKGRKNDKNIG.........PLEIKVVRRGAFDELMS
BnaC09.GH3-14.a     .......EECCFTVEESL.DSVYRKGRKNDKNIG.........PLEIKVVRRGAFDELMN
BnaA03.GH3-15.a     .......EECCFTMEESL.DSIYRKGRKNDKNIG.........PLEIKVVKSGAFDELMN
BnaC03.GH3-15.a     ..............EQS...........................................
BnaAX.GH3-16.a      .......EECCFTIEKSL.NSVYRKLRKNDGTVG.........PLEIKVVRRGAFDELMN
BnaC02.GH3-16.a     .......EECCFTIEKSL.NSVYRKLRMNDGTVG.........PLEIKVVKRGAFDELMN
BnaA07.GH3-17.a     .......EKCCSEVEDRL.DYVYRRCRNKDKSIG.........PLEIRVVSLGTFDLLMD
BnaA08.GH3-17.b     .......EDCCSEVEDCL.DYVYRRCRNKDRSIG.........ALEIRVVSLGTFDALMD
BnaA09.GH3-17.c     .......ELCCSEVEDYL.DYVYRRCRNKDKSIG.........PLEIRVVSLGTFDLLMD
BnaC08.GH3-17.a     .......EDCCSEVEDCL.DYVYRRCRNKDGSIG.........ALEMRVVSLGTFDALMD
BnaCX.GH3-17.b      .......ELCCSEVEDYL.DYVYRRCRNKDKSIG.........PLEIRVVSLGTFDLLMD
BnaCX.GH3-17.c      .......ELCCSEVEDCL.DYVYRRCRNKDKSIG.........PLEIRVVSLGTFDLLMD
BnaA05GH3-18.a      .......MGCCYVLEESF.GSTYRLKR.RTGLIG.........ALEIRVVEQGTFNSLTD
BnaCX.GH3-18.a      .......VDYCCVVEESM.SSLYRLRRVKDGPIG.........PLELRVVQQGTFDSLME
BnaA06.GH3-19.a     .......VECCCVMEESL.NGSYRRFRRKNGSIG.........ALEIRVVQQGTFDSLMD
BnaA09.GH3-19.b     .......VECCCIMEESL.SSLYRRLRGDEGSIG.........ALEIRVVQQGTFDSLME
consensus>70        .........cc...eesl.d..y...r..d..ig..........leirvv..gtfd.lmd
```

```
                          550                 560        570
                            .                    .          .
AtGH3-1           YAI.SRGASINQ.....YKVPRC.....VN.F.TPIVELLDSR.................
AtGH3-2           YAI.SRGASINQ.....YKVPRC.....VS.F.TPIMELLDSR.................
AtGH3-3           YAI.SRGASINQ.....YKVPRC.....VS.F.TPIMELLDSR.................
AtGH3-4           FSI.SRGSSINQ.....YKVPRC.....VS.L.TPIMKLLDSR.................
AtGH3-5           YAI.SLGASINQ.....YKTPRC.....VK.F.APIIELLNSR.................
AtGH3-6           YAI.SLGASINQ.....YKTPRC.....VK.F.APIIELLNSR.................
AtGH3-7           YFI.SQGASIGQ.....YKTPRC.....IK.S.GKALEVLEEN.................
AtGH3-8           FFI.TQGASSTQ.....YKTPIC.....IK.S.TEALVILEEN.................
AtGH3-9           LII.SQGGSFNQ.....YKTPRC.....VK.SNSATFKLLNGH.................
AtGH3-10          RCV.GKCGGLNQ.....FKTPRC.....T..TNSVMLDILNDS.................
AtGH3-11          HFL.GLGSSAGQ.....FKMPRC.....VKPSNAKVLQILCEN.................
AtGH3-12          FFI.SQGASTGQ.....YKTPRC.....IK.S.GKALQVLETC.................
AtGH3-13          FFL.SRGSSVSQ.....YKTPRS.....VK.T.EEAVKILEAN.................
AtGH3-14          FFL.SRGSSVSQ.....YKTPRS.....VT.N.EEALKILEAN.................
AtGH3-15          FFL.SRGSSVSQ.....YKTPRS.....VT.N.EEALKILEAN.................
AtGH3-16          FFL.SRGSSVSQ.....YKTPRS.....VT.H.EEALNILESN.................
AtGH3-17          FCV.SQGSSLNQ.....YKTPRC.....VK.S.GGALEILDSR.................
AtGH3-18          YFI.SQGGSIAQ.....YKTPIC.....IN.S.SEALAVLENK.................
AtGH3-19          YFI.SKGGSVAQ.....YKTPMC.....IN.S.SETLAVLEDK.................
BraX.GH3-1.a      YAI.SRGASINQ.....YKVPRC.....VN.F.TPIVELLDSR.................
BraX.GH3-2.a      YAI.SRGASINQ.....YKVPRC.....VS.F.TPIMELLDSR.................
BraA09.GH3-3.a    YAI.SRGASINQ.....YKVPRC.....VS.F.TPIMELLDSR.................
BraA01.GH3-5.a    YAI.SLGASINQ.....YKTPRC.....VK.F.APIIELLNSR.................
BraA03.GH3-5.b    YAI.SLGASINQ.....YKTPRC.....VK.F.APIIELLNSR.................
BraA02.GH3-6.a    YAI.SLGASINQ.....YKTPRC.....VK.F.APIIELLNSR.................
BraA03.GH3-6.b    YAI.SLGASINQ.....YKTPRC.....VK.F.APIIELLNSR.................
BraA06.GH3-7.a    LSI.SKGASITQ.....YKTPTC.....IT.S.EESLQVLETN.................
BraA08.GH3-7.b    FFI.SQGASIGQ.....YKTPRC.....IG.S.GKPLELLEKN.................
BraA02.GH3-8.a    FFI.TQGASSTQ.....YKTPIC.....IK.S.SEALAILEEK.................
BraA03.GH3-8.b    FFI.SQGASSTQ.....YKTPIC.....IK.S.TEAIAILERK.................
BraA03.GH3-8.c    YFI.SQGASLAQ.....YKTPRC.....IK.S.SEALQVLENR.................
BraA03.GH3-8.d    YFI.AQGASIGQ.....YKTPRC.....IK.S.SAALELRP...................
BraA06.GH3-8.e    FFV.CRGSSISQ.....YKTPMC.....LE.S.AEALKVLEDK.................
BraA06.GH3-8.f    FFV.SRGCSITQ.....YKTPIC.....LN.S.AEALKVLGDK.................
BraA09.GH3-8.g    FSI.SQGASPSQ.....YKTPMC.....IK.S.SEALVVLDNN.................
BraA09.GH3-8.h    FFV.SRGASITQ.....YKTPIC.....IN.S.VEASRVLEDK.................
BraA10.GH3-8.i    LAI.SQGASPSQ.....YKTPIC.....IN.S.SEASAVLESN.................
BraA09.GH3-10.a   RCV.GKCGGLNQ.....FKTPRC.....T..TNSVMLDILDGS.................
BraA03.GH3-11.a   HFL.GLGSSAGQ.....FKMPRC.....VKPSNVKVLQILCDN.................
BraA04.GH3-11.b   HFL.GLGSSAGQ.....FKMPRC.....VKPSNGKVLKILCEN.................
BraA05.GH3-11.c   HFL.GLGSSAGQ.....FKMPRC.....VKPSNAKVLQILCEN.................
BraA02.GH3-12.a   FFI.KQGASIGQ.....YKTPRC.....IK.S.GKALEFMEDY.................
BraA03.GH3-12.b   FFI.SQGASIGQ.....YKTPRC.....IK.S.VKALEFMEEC.................
BraA06.GH3-12.c   LSI.SKGTSVNQ.....YKTPTC.....IT.S.EKGVQVLETN.................
BraA10.GH3-12.d   FFI.NQGASTGQ.....YKTPRC.....IK.S.GKALELMEDR.................
BraA10.GH3-14.a   FFV.SRGTSVSQ.....YKTPRS.....VK.N.EEALKILEAT.................
BraA03.GH3-15.a   FFV.SRGSSVSQ.....YKTPRS.....VT.N.EEALKVLEAS.................
BraA07.GH3-15.b   FFV.SRGSSVSQ.....FKTPRS.....LT.N.EEALKILEAT.................
BraA02.GH3-16.a   LFL.SRGSSVSQ.....YKTPRS.....VT.S.EEAMKILEEA.................
BraA07.GH3-17.a   FCV.SQGSSVNQ.....YKTPRC.....VK.S.GGALEILDSR.................
BraA09.GH3-17.b   FCV.SQGSSVNQ.....YKTPRC.....VK.P.GGALEILDSR.................
BraAX.GH3-17.c    FSI.SQGSSVNQ.....YKTPRC.....VK.S.GGALQILDSR.................
BraA04.GH3-19.a   YFI.SQGAFAHQ.....YKTPLC...................K.................
BraA06.GH3-19.b   FFV.SRGSSISQ.....YKTPMC.....IN.S.AEALKVLEDK.................
BraA09.GH3-19.c   FFL.SRGSSISQ.....YKTPIC.....IK.S.TEALKLLEDK.................
BraA09.GH3-19.d   FFV.TRGASITQ.....YKTPIC.....IN.S.VEALKVLEDK.................
BraAX.GH3-19.e    YFI.SRGSSRAQ.....YKTPLC.....IN.S.SEASAVLENK.................
BolC09.GH3-1.a    YAI.SRGASINQ.....YKVPRC.....VN.F.TPIVELLDSR.................
BolC01.GH3-2.a    YAI.SRGASINQ.....YKVPRC.....VS.F.TPIMELLDSR.................
BolC08.GH3-3.a    YAI.SRGASINQ.....YKVPRC.....VT.F.TPIMELLDSR.................
BolC01.GH3-5.a    YAI.SLGASINQ.....YKTPRC.....VK.F.APIIELLNSR.................
BolC07.GH3-5.b    YAI.SLGASINQ.....YKTPRC.....VK.F.APIIELLNSR.................
BolC02.GH3-6.a    YAI.SLGASINQ.....YKTPRC.....VK.F.APIIELLNSR.................
BolC03.GH3-6.b    YAI.SLGASINQ.....YKTPRC.....VK.F.APIIELLNSR.................
BolC06.GH3-7.a    FSI.SKGTSITQ.....YKTPTC.....IT.S.EEGLQVLEIN.................
BolC01.GH3-8.a    YFI.AQGATLTQ.....YKTPRC.....IK.S.PEALQVLESKSGKLKIAKQEIKNGKHV
BolC03.GH3-8.b    ............................................................
BolC07.GH3-8.c    YFI.SQGASLAQ.....YKTPRC.....IK.S.SEALQVLENR.................
BolC08.GH3-8.d    FFV.SRGASITQ.....YKTPIC.....IN.S.VEALKVLEDK.................
BolC09.GH3-8.e    FTLGCTKSRWVQDWFSGYKETLG.....EKDVLQDALSDSNMK..........YYHMRHL
BolC09.GH3-10.a   RCV.GKCGGLNQ.....FKTPRC.....T..TNSVMLDILDGS.................
BolC03.GH3-11.a   HFL.GLGSSAGQ.....FKMPRC.....VKPNNVKVLQILCDN.................
BolC04.GH3-11.b   HFL.GLGSSAGQ.....FKMPRC.....VKPSNAKVLKILCEN.................
BolCX.GH3-11.c    HFL.GLGSSAGQ.....FKMPRC.....VKPSNAKVLQILCEN.................
BolC03.GH3-12.a   FFI.SQGASIGQ.....YKTPRC.....IK.S.VKALEFMEEC.................
BolC09.GH3-12.b   FFI.NQGASIGQ.....YKAPRC.....IK.S.GKALEFMEDR.................
BolC09.GH3-14.a   FFV.SRGTSVSQ.....YKTPRS.....VK.N.EEALKILEAT.................
BolC03.GH3-15.a   FFV.SRGSSVSQ.....YKTPRS.....VT.D.EEAVKVLEAS.................
BolCX.GH3-16.a    LFL.SRGSSVSQ.....YKTPRS.....VT.S.EEAMKILEEA.................
BolC07.GH3-17.a   FCV.SQGSSVNQ.....YKTPRC.....VK.S.GGALEILDSR.................
BolC08.GH3-17.b   FSI.SQGSSVNQ.....YKTPRC.....VK.S.GGALQILDSR.................
BolC08.GH3-19.a   FFV.SQGSSISQ.....YKTPIC.....IK.S.TEALKVLDDK.................
BnaA09R.GH3-1.a   YAI.SRGASINQ.....YKVPRC.....VN.F.TPIVELLDSR.................
BnaCX.GH3-1.a     YAI.SRGASINQ.....YKVPRC.....VN.F.TPIVELLDSR.................
BnaA01.GH3-2.a    YAI.SRGASINQ.....YKVPRC.....VS.F.TPIMELLDSR.................
BnaC01.GH3-2.a    YAI.SRGASINQ.....YKVPRC.....VS.F.TPIMELLDSR.................
BnaA09.GH3-3.a    YAI.SRGASINQ.....YKVPRC.....VS.F.TPIMELLDSR.................
BnaC08.GH3-3.a    YAI.SRGASINQ.....YKVPRC.....VS.F.TPITELLDSR.................
BnaA01.GH3-5.a    YAI.SLGASINQ.....YKTPRC.....VK.F.APIIELLNSR.................
BnaA03.GH3-5.b    YAI.SLGASINQ.....YKTPRC.....VK.F.APIIELLNSR.................
BnaC01.GH3-5.a    YAI.CLGASINQ.....YKTPRC.....VK.F.APIIELLNSR.................
BnaC07.GH3-5.b    YAI.SLGASINQ.....YKTPRC.....VK.F.APIIELLNSR.................
BnaA03.GH3-6.a    YAI.SLGASINQ.....YKTPRC.....VK.F.APIIELLNSR.................
BnaAX.GH3-6.b     YAI.SLGASINQ.....YKTPRC.....VK.F.APIIELLNSR.................
BnaC03.GH3-6.a    YAI.SLGASINQ.....YKTPRC.....VK.F.APIIELLNSR.................
BnaCX.GH3-6.b     YAI.SLGASINQ.....YKTPRC.....VK.F.APIIELLNSR.................
BnaA06.GH3-7.a    LSI.SKGASITQ.....YKTPTC.....IT.S.EEGLQVLETN.................
BnaA08.GH3-7.b    FFI.SQGASIGQ.....YKTPRC.....IG.S.GKPLELLEKN.................
BnaC06.GH3-7.a    LSI.SKGTSITQ.....YKTPTC.....IT.S.EEGLQVLEIN.................
BnaA03.GH3-8.a    FFI.SQGASSTQ.....YKTPIC.....IK.S.TEAIAILERK.................
BnaA03.GH3-8.b    YFI.SQGASLAQ.....YKTPRC.....IK.S.SEALQVLENR.................
BnaA03.GH3-8.c    YFI.AQGASLGQ.....YKTPRC.....IK.S.SKALEVLENR.................
BnaA06.GH3-8.d    FFV.SRGSSISQ.....YKTPMC.....LE.S.AEALKVLEDK.................
```

```
BnaA06.GH3-8.e      FFV.SRGCSITQ.....YKTPIC.....IS.S.AEALKVLEDK.................
BnaA09.GH3-8.f      FFILSRLKFVFV.....HLLACCGLDTKLD.T.ISPS.......................
BnaA09.GH3-8.g      YYVVSRGSSISQ.....YKTPIC.....IN.S.AEALEVLEEK.................
BnaAX.GH3-8.h       FFI.TQGASSTQ.....YKTPIC.....IK.S.SEALAILEEK.................
BnaAX.GH3-8.i       FFV.SRGASITQ.....YKTPIC.....IN.S.VEALKVLEDK.................
BnaC01.GH3-8.a      YFI.AQGATLTQ.....YKTPRC.....IK.S.PEALQVLESKSGKLKIAKQEIKNGKHV
BnaC02.GH3-8.b      FFI.TQGASSTQ.....YKTPIC.....IK.S.SEALAILEEK.................
BnaC02.GH3-8.c      FFI.TQGASSTQ.....YKTPIC.....IK.S.SEALAILEEK.................
BnaC02.GH3-8.d      FFI.TQGASSTQ.....YKTPIC.....IK.S.SEALAILEEK.................
BnaC07.GH3-8.e      YFI.SQGASLAQ.....YKTPRC.....IK.S.SEALQVLENR.................
BnaC09.GH3-8.f      FSI.SQGASPSQ.....YKTPMC.....IK.S.SEALVVLDNN.................
BnaCX.GH3-8.g       FFV.SRGASITQ.....YKTPIC.....IN.S.AEALKVLEDK.................
BnaA05.GH3-9.a      MII.SQGGSLNQ.....YKTPRC.....VK.SNSAMLKLLDGH.................
BnaC04.GH3-9.a      MII.SQGGSLNQ.....YKTPRC.....VK.SNSAMLKLLDGH.................
BnaA03.GH3-10.a     ......................C.....T..TNSVMLDILDAS.................
BnaA09.GH3-10.b     RCV.GKCGGLNQ.....FKTPRC.....T..TNSVMLDILDGS.................
BnaC09.GH3-10.a     RCV.GKCGGLNQ.....FKTPRC.....T..TNSVMLDILDGS.................
BnaA03.GH3-11.a     HFL.GLGSSAGQ.....FKMPRC.....VKPSNVKVLQILCDN.................
BnaA04R.GH3-11.b    HFL.GLGSSAGQ.....FKMPRC.....VKPSNGKVLKILCEN.................
BnaA05.GH3-11.c     HFL.GLGSSAGQ.....FKMPRC.....VKPSNAKVLQILCEN.................
BnaC04.GH3-11.a     HFL.GLGSSAGQ.....FKMPRC.....VKPSNAKVLKILCEN.................
BnaCX.GH3-11.b      HFL.GLGSSAGQ.....FKMPRC.....VKPNNVKVLQILCDN.................
BnaA02.GH3-12.a     FFI.KQGASIGQ.....YKTPRC.....IK.S.GKALEFMENY.................
BnaA03.GH3-12.b     FFI.TQGASIGQ.....YKTPRC.....IK.S.VKALEFMEEC.................
BnaA06.GH3-12.c     LSI.SKGTSVNQ.....YKTPTC.....IT.S.EKGVQVLETN.................
BnaA10.GH3-12.d     FFI.NQGASTGQ.....YKTPRC.....IK.S.GKALELMEDR.................
BnaC02.GH3-12.a     FFI.KQGASIGQ.....YKTPRC.....IK.S.GKALEFMEDY.................
BnaC03.GH3-12.b     FFI.SQGASIGQ.....YKTPRC.....IK.S.VKALEFMEEC.................
BnaC09.GH3-12.c     FFI.NQGASIGQ.....YKAPRC.....IK.S.GKALEFMEDR.................
BnaA10.GH3-14.a     FFV.SRGTSVSQ.....YKTPRS.....VK.N.EEALKILEAT.................
BnaC09.GH3-14.a     FFV.SRGTSVSQ.....YKTPRS.....VK.N.EEALKILEAT.................
BnaA03.GH3-15.a     FFV.SRGSSVSQ.....YKTPRS.....VT.N.EEALKVLEAS.................
BnaC03.GH3-15.a     ............................................................
BnaAX.GH3-16.a      LFL.SRGSSVSQ.....YKTPRS.....VT.S.EEAMKILEEA.................
BnaC02.GH3-16.a     LFL.SRGSSVSQ.....YKTPRS.....VT.S.EEAMKILEEA.................
BnaA07.GH3-17.a     FCV.SQGSSVNQ.....YKTPRC.....VK.S.GGALEILDSR.................
BnaA08.GH3-17.b     FSI.SQGSSVNQ.....YKTPRC.....VK.S.GGALQILDSR.................
BnaA09.GH3-17.c     FCV.SQGSSVNQ.....YKTPRC.....VK.P.GGALEILDSR.................
BnaC08.GH3-17.a     FSI.SQGSSVNQ.....YKTPRC.....VK.S.GGALQILDSR.................
BnaCX.GH3-17.b      FCV.SQGSSVNQ.....YKTPRC.....VK.P.GGALEILDSR.................
BnaCX.GH3-17.c      FCV.SQGSSVNQ.....YKTPRC.....VK.P.GGALEILDSR.................
BnaA05GH3-18.a      YFI.SRGSSRAQ.....YKTPLC.....IN.S.SEASAVLENK.................
BnaCX.GH3-18.a      FFV.ARGSSISQ.....YKTPMC.....IN.S.AEALKVLEDK.................
BnaA06.GH3-19.a     FFV.SRGSSISQ.....YKTPMC.....IN.S.AEALKVLEDK.................
BnaA09.GH3-19.b     FFL.SRSSSISQ.....YKTPIC.....IK.S.TEALKLLEDK.................
consensus>70        f.i.s.g.s..q.....yktprc.....v.......l..le...................
```

```
                                                   580       590
                                                    .         .
AtGH3-1           ..............V..........VSAHFSPSLPHWTPERRRR................
AtGH3-2           ..............V..........VSAHFSPSLPHWSPERRR.................
AtGH3-3           ..............V..........VSTHFSPALPHWSPERRR.................
AtGH3-4           ..............V..........VSAHFSPSLPHWSPERRH.................
AtGH3-5           ..............V..........VDSYFSPKCPKWVPGHKQWGSN.............
AtGH3-6           ..............V..........VDSYFSPKCPKWSPGHKQWGSN.............
AtGH3-7           ..............V..........VATFFST............................
AtGH3-8           ..............V..........HACFFTDKSLSLNFSS...................
AtGH3-9           ..............V..........MASFFSPRDPTWVP.....................
AtGH3-10          ..............T..........IKRFRSSAYD.........................
AtGH3-11          ..............V..........VSSYFSTAF..........................
AtGH3-12          ..............V..........VAKFFSI............................
AtGH3-13          ..............V..........VSEFLSQETPPWG......................
AtGH3-14          ..............V..........VSEFLSQKTPSWELHELHSSR..............
AtGH3-15          ..............V..........ISEFLSRKIPSWELHELHSGR..............
AtGH3-16          ..............V..........VSEFLSRKTPSWELHELHSSR..............
AtGH3-17          ..............V..........IGRFFSKRVPQWEPLGLDS................
AtGH3-18          ..............V..........LARFFSEKSPPLDS.....................
AtGH3-19          ..............V..........IARFYSQKSPPLNL.....................
BraX.GH3-1.a      ..............V..........VSAHFSPALPHWTPERRRR................
BraX.GH3-2.a      ..............V..........VSAHFSPSLPHWSPERRR.................
BraA09.GH3-3.a    ..............V..........ASTHFSPALPQWSPERRR.................
BraA01.GH3-5.a    ..............V..........VDSYFSPKCPKWVPGHKQWGSN.............
BraA03.GH3-5.b    ..............V..........VDSYFSPKCPKWVPGHKQWGSN.............
BraA02.GH3-6.a    ..............V..........VKSYFSPKCPKWTPGHKQWGSN.............
BraA03.GH3-6.b    ..............V..........VKTYFSPKCPKWIPGHKQWGK..............
BraA06.GH3-7.a    ..............V..........VARFFSTLRASHVLKP...................
BraA08.GH3-7.b    ..............V..........VATFFSTGDCSFKN.....................
BraA02.GH3-8.a    ..............V..........RSRFFTDKVPFL.......................
BraA03.GH3-8.b    ..............V..........RARFFTDKSLSLKFSS...................
BraA03.GH3-8.c    ..............V..........LARFFSEKLP.........................
BraA03.GH3-8.d    ............................................................
BraA06.GH3-8.e    ..............V..........LARFFSDKSPPI.......................
BraA06.GH3-8.f    ..............V..........LARFFNDKSPTI.......................
BraA09.GH3-8.g    ..............V..........LARFFSDKSPPF.......................
BraA09.GH3-8.h    ..............V..........LARFFSDKSPTI.......................
BraA10.GH3-8.i    ..............V..........LACFFSDKSPTL.......................
BraA09.GH3-10.a   ..............T..........VKRFSSSAYN.........................
BraA03.GH3-11.a   ..............V..........VSRFFSTAFE.........................
BraA04.GH3-11.b   ..............A..........VSRFFSTAFD.........................
BraA05.GH3-11.c   ..............V..........VSRFFSTAFE.........................
BraA02.GH3-12.a   ..............V..........VARFFST............................
BraA03.GH3-12.b   ..............V..........VARFFST............................
BraA06.GH3-12.c   ..............V..........VAKFFSTLSASHFA.....................
BraA10.GH3-12.d   ..............V..........VAKFFSS............................
BraA10.GH3-14.a   ..............V..........VSKSFSRKVPSWELHELHSSR..............
BraA03.GH3-15.a   ..............V..........VSKFVSKKTPSWELHELHSSR..............
BraA07.GH3-15.b   ..............V..........VSKFVSRKVPSWELHELHSSR..............
BraA02.GH3-16.a   ..............V..........VSEFLSTKTPSWEPREMHSGR..............
BraA07.GH3-17.a   ..............V..........IGRFFSQRVPQWEPLGLDS................
BraA09.GH3-17.b   ..............V..........IGRFFSQRVPQWQPLGLDS................
BraAX.GH3-17.c    ..............V..........IGRFFTKRVPQWEPLGLDS................
BraA04.GH3-19.a   ..............V.............................................
BraA06.GH3-19.b   ..............V..........LARFFSDKSPPI.......................
BraA09.GH3-19.c   ..............V..........LARFFSDRSPTL.......................
BraA09.GH3-19.d   ..............V..........LARFFSDKSPPI.......................
BraAX.GH3-19.e    ..............V..........LARFHSEKSPPLDL.....................
BolC09.GH3-1.a    ..............V..........VSAHFSPALPHWTPERRRR................
BolC01.GH3-2.a    ..............V..........VSAHFSPSLPHWSPERRR.................
BolC08.GH3-3.a    ..............V..........ASTHFSPALPQWSPERRR.................
BolC01.GH3-5.a    ..............V..........VDSYFSPKCPKWVPGHKQWGSN.............
BolC07.GH3-5.b    ..............V..........VDSYFSPKCPKWVPGHKQWGSN.............
BolC02.GH3-6.a    ..............V..........VKSYFSPKCPKWTPGHKQWGSN.............
BolC03.GH3-6.b    ..............V..........VKTYFSPKCPKWIPGHKQWGSN.............
BolC06.GH3-7.a    ..............V..........VTKFFSTLSASHFA.....................
BolC01.GH3-8.a    LLVDPKSVTDINGAVQ........LTNMFLMDRNSSVMEFLPKGWLKLAGVGQLVYQWVA
BolC03.GH3-8.b    ............................................................
BolC07.GH3-8.c    ..............V..........LARFFSEKLP.........................
BolC08.GH3-8.d    ..............V..........LARFFSDKSPPI.......................
BolC09.GH3-8.e    RTLGGAFRDTCRHFV..........IMVFLTR............................
BolC09.GH3-10.a   ..............T..........VKRFSSSAYN.........................
BolC03.GH3-11.a   ..............V..........VSRFFSTAFE.........................
BolC04.GH3-11.b   ..............V..........VSRFFSTAFD.........................
BolCX.GH3-11.c    ..............V..........VSRFFSTAFE.........................
BolC03.GH3-12.a   ..............V..........VARFFST............................
BolC09.GH3-12.b   ..............V..........VAKFSSA............................
BolC09.GH3-14.a   ..............V..........VSKSFSQKVPSWELHELHSSR..............
BolC03.GH3-15.a   ..............V..........VSKFVSRKTPSWELHELHSSR..............
BolCX.GH3-16.a    ..............V..........VSEFLSTKTPSWEPREMHSGR..............
BolC07.GH3-17.a   ..............V..........IGSFFSKRVPQWEPLGLDS................
BolC08.GH3-17.b   ..............V..........IGKFFTKRVPQWEPLGLDS................
BolC08.GH3-19.a   ..............V..........LARFFSDRSPTL.......................
BnaA09R.GH3-1.a   ..............V..........VSAHFSPALPHWTPERRRR................
BnaCX.GH3-1.a     ..............V..........VSAHFSPALPHWTPERRRR................
BnaA01.GH3-2.a    ..............V..........VSAHFSPSLPHWSPERRR.................
BnaC01.GH3-2.a    ..............V..........VSAHFSPSLPHWSPERRR.................
BnaA09.GH3-3.a    ..............V..........ASTHFSPALPQWSPERRR.................
BnaC08.GH3-3.a    ..............V..........ASTHFSPALPQWSPERRR.................
BnaA01.GH3-5.a    ..............V..........VDSYFSPKCPKWVPGHKQWGSN.............
BnaA03.GH3-5.b    ..............V..........VDSYFSPKCPKWVPGHKQWGSN.............
BnaC01.GH3-5.a    ..............V..........VDSYFSPKCPKWVPGHKQWGSN.............
BnaC07.GH3-5.b    ..............V..........VDSYFSPKCPKWVPGHKQWGSN.............
BnaA03.GH3-6.a    ..............V..........VKTYFSPKCPKWIPGHKQWGSN.............
BnaAX.GH3-6.b     ..............V..........VKSYFSPKCPKWTPGHKQWGSN.............
BnaC03.GH3-6.a    ..............V..........VKTYFSPKCPKWIPGHKQWGSN.............
BnaCX.GH3-6.b     ..............V..........VKSYFSPKCPKWTPGHKQWGSN.............
BnaA06.GH3-7.a    ..............V..........VARFFSTLRASHVLKP...................
BnaA08.GH3-7.b    ..............V..........VATFFSTGDCSFKN.....................
BnaC06.GH3-7.a    ..............V..........VTKFFSTLSASHFA.....................
BnaA03.GH3-8.a    ..............V..........RARFFTDKSLSLKFSS...................
BnaA03.GH3-8.b    ..............V..........LARFFSEKLP.........................
BnaA03.GH3-8.c    ..............V........MAHERFIAY............................
BnaA06.GH3-8.d    ..............V..........LARFFSDKSPPI.......................
```

```
BnaA06.GH3-8.e      ..............V..........LARFFNDKSPTI.......................
BnaA09.GH3-8.f      ........................ ..SYISKRIPVWLV.....................
BnaA09.GH3-8.g      ..............V..........LARFFSDKSPPL.......................
BnaAX.GH3-8.h       ..............V..........RSRFFTDKVPFL.......................
BnaAX.GH3-8.i       ..............V..........LARFFSDKSPPI.......................
BnaC01.GH3-8.a      LLVDPKSVTDINGAVQVRCVNYVLITNMFLMDRNSSVMEFLPKGWLKLAGVGQLVYQWVA
BnaC02.GH3-8.b      ..............V..........RSRFFTDKVPFL.......................
BnaC02.GH3-8.c      ..............V..........RSRFFTDKVPFL.......................
BnaC02.GH3-8.d      ..............V..........RSRFFTDKVPFL.......................
BnaC07.GH3-8.e      ..............V..........LARFFSEKLP.........................
BnaC09.GH3-8.f      ..............V..........LARFFSDKSPPF.......................
BnaCX.GH3-8.g       ..............V..........LARFFSDKSPPYLILA...................
BnaA05.GH3-9.a      ..............V..........TGSFFSPRDPTWSA.....................
BnaC04.GH3-9.a      ..............V..........TGSFFSPRDPTWSP.....................
BnaA03.GH3-10.a     ..............T..........VKIFCSSAYD.........................
BnaA09.GH3-10.b     ..............T..........VKRFSSSAYN.........................
BnaC09.GH3-10.a     ..............T..........VKRFSSSAYN.........................
BnaA03.GH3-11.a     ..............V..........VSRFFSTAFE.........................
BnaA04R.GH3-11.b    ..............A..........VSRFFSTAFD.........................
BnaA05.GH3-11.c     ..............V..........VSRFFSTAFE.........................
BnaC04.GH3-11.a     ..............V..........VSRFFSTAFD.........................
BnaCX.GH3-11.b      ..............V..........VSRFFSTAFE.........................
BnaA02.GH3-12.a     ..............V..........VARFFST............................
BnaA03.GH3-12.b     ..............V..........VARFFST............................
BnaA06.GH3-12.c     ..............V..........VAKFFSTLSASHFA.....................
BnaA10.GH3-12.d     ..............V..........VAKFFSS............................
BnaC02.GH3-12.a     ..............V..........VASFFST............................
BnaC03.GH3-12.b     ..............V..........VARFFST............................
BnaC09.GH3-12.c     ..............V..........VAKFSSA............................
BnaA10.GH3-14.a     ..............V..........VSKSFSRKVPSWELHELHSSR..............
BnaC09.GH3-14.a     ..............V..........VSKSFSQKVPSWELHELHSSR..............
BnaA03.GH3-15.a     ..............V..........VSKFVSKKTPSWELHELHSSR..............
BnaC03.GH3-15.a     ............................................................
BnaAX.GH3-16.a      ..............V..........VSEFLSTKTPSWEPREMHSGR..............
BnaC02.GH3-16.a     ..............V..........VSEFLSTKTPSWEPREMHSGR..............
BnaA07.GH3-17.a     ..............V..........IGRFFSQRVPQWEPLGLDS................
BnaA08.GH3-17.b     ..............V..........IGRFFTKRVPQWEPLGLDS................
BnaA09.GH3-17.c     ..............V..........IGRFFSQRVPQWQPLGLDS................
BnaC08.GH3-17.a     ..............V..........IGKFFTKRVPQWEPLGLDS................
BnaCX.GH3-17.b      ..............V..........IGRFFSQRVPQWQPLGLDS................
BnaCX.GH3-17.c      ..............V..........IGRFFSQRVPRWQPLGLDS................
BnaA05GH3-18.a      ..............V..........LARFHSEKSPPLDL.....................
BnaCX.GH3-18.a      ..............V..........LARFFSDKSPPI.......................
BnaA06.GH3-19.a     ..............V..........LARFFSDKSPPI.......................
BnaA09.GH3-19.b     ..............V..........LARFFSNRSPTL.......................
consensus>70        ..............v.............ffs.............................
```

```
AtGH3-1             ............................................................
AtGH3-2             ............................................................
AtGH3-3             ............................................................
AtGH3-4             ............................................................
AtGH3-5             ............................................................
AtGH3-6             ............................................................
AtGH3-7             ............................................................
AtGH3-8             ............................................................
AtGH3-9             ............................................................
AtGH3-10            ............................................................
AtGH3-11            ............................................................
AtGH3-12            ............................................................
AtGH3-13            ............................................................
AtGH3-14            ............................................................
AtGH3-15            ............................................................
AtGH3-16            ............................................................
AtGH3-17            ............................................................
AtGH3-18            ............................................................
AtGH3-19            ............................................................
BraX.GH3-1.a        ............................................................
BraX.GH3-2.a        ............................................................
BraA09.GH3-3.a      ............................................................
BraA01.GH3-5.a      ............................................................
BraA03.GH3-5.b      ............................................................
BraA02.GH3-6.a      ............................................................
BraA03.GH3-6.b      ............................................................
BraA06.GH3-7.a      ............................................................
BraA08.GH3-7.b      ............................................................
BraA02.GH3-8.a      ............................................................
BraA03.GH3-8.b      ............................................................
BraA03.GH3-8.c      ............................................................
BraA03.GH3-8.d      ............................................................
BraA06.GH3-8.e      ............................................................
BraA06.GH3-8.f      ............................................................
BraA09.GH3-8.g      ............................................................
BraA09.GH3-8.h      ............................................................
BraA10.GH3-8.i      ............................................................
BraA09.GH3-10.a     ............................................................
BraA03.GH3-11.a     ............................................................
BraA04.GH3-11.b     ............................................................
BraA05.GH3-11.c     ............................................................
BraA02.GH3-12.a     ............................................................
BraA03.GH3-12.b     ............................................................
BraA06.GH3-12.c     ............................................................
BraA10.GH3-12.d     ............................................................
BraA10.GH3-14.a     ............................................................
BraA03.GH3-15.a     ............................................................
BraA07.GH3-15.b     ............................................................
BraA02.GH3-16.a     ............................................................
BraA07.GH3-17.a     ............................................................
BraA09.GH3-17.b     ............................................................
BraAX.GH3-17.c      ............................................................
BraA04.GH3-19.a     ............................................................
BraA06.GH3-19.b     ............................................................
BraA09.GH3-19.c     ............................................................
BraA09.GH3-19.d     ............................................................
BraAX.GH3-19.e      ............................................................
BolC09.GH3-1.a      ............................................................
BolC01.GH3-2.a      ............................................................
BolC08.GH3-3.a      ............................................................
BolC01.GH3-5.a      ............................................................
BolC07.GH3-5.b      ............................................................
BolC02.GH3-6.a      ............................................................
BolC03.GH3-6.b      ............................................................
BolC06.GH3-7.a      ............................................................
BolC01.GH3-8.a      NWSRIRHEGTWHDPVGETC.........................................
BolC03.GH3-8.b      ............................................................
BolC07.GH3-8.c      ............................................................
BolC08.GH3-8.d      ............................................................
BolC09.GH3-8.e      ............................................................
BolC09.GH3-10.a     ............................................................
BolC03.GH3-11.a     ............................................................
BolC04.GH3-11.b     ............................................................
BolCX.GH3-11.c      ............................................................
BolC03.GH3-12.a     ............................................................
BolC09.GH3-12.b     ............................................................
BolC09.GH3-14.a     ............................................................
BolC03.GH3-15.a     ............................................................
BolCX.GH3-16.a      ............................................................
BolC07.GH3-17.a     ............................................................
BolC08.GH3-17.b     ............................................................
BolC08.GH3-19.a     ............................................................
BnaA09R.GH3-1.a     ............................................................
BnaCX.GH3-1.a       ............................................................
BnaA01.GH3-2.a      ............................................................
BnaC01.GH3-2.a      ............................................................
BnaA09.GH3-3.a      ............................................................
BnaC08.GH3-3.a      ............................................................
BnaA01.GH3-5.a      ............................................................
BnaA03.GH3-5.b      ............................................................
BnaC01.GH3-5.a      ............................................................
BnaC07.GH3-5.b      ............................................................
BnaA03.GH3-6.a      ............................................................
BnaAX.GH3-6.b       ............................................................
BnaC03.GH3-6.a      ............................................................
BnaCX.GH3-6.b       ............................................................
BnaA06.GH3-7.a      ............................................................
BnaA08.GH3-7.b      ............................................................
BnaC06.GH3-7.a      ............................................................
BnaA03.GH3-8.a      ............................................................
BnaA03.GH3-8.b      ............................................................
BnaA03.GH3-8.c      ............................................................
BnaA06.GH3-8.d      ............................................................
```

```
BnaA06.GH3-8.e       ............................................................
BnaA09.GH3-8.f       ............................................................
BnaA09.GH3-8.g       ............................................................
BnaAX.GH3-8.h        ............................................................
BnaAX.GH3-8.i        ............................................................
BnaC01.GH3-8.a       NWSRIRHEGTWHDPVGETLAASLLMAIMFSAMITSSRATKQLTKKQTLKPKFFGQPFPKP
BnaC02.GH3-8.b       ............................................................
BnaC02.GH3-8.c       ............................................................
BnaC02.GH3-8.d       ............................................................
BnaC07.GH3-8.e       ............................................................
BnaC09.GH3-8.f       ............................................................
BnaCX.GH3-8.g        ............................................................
BnaA05.GH3-9.a       ............................................................
BnaC04.GH3-9.a       ............................................................
BnaA03.GH3-10.a      ............................................................
BnaA09.GH3-10.b      ............................................................
BnaC09.GH3-10.a      ............................................................
BnaA03.GH3-11.a      ............................................................
BnaA04R.GH3-11.b     ............................................................
BnaA05.GH3-11.c      ............................................................
BnaC04.GH3-11.a      ............................................................
BnaCX.GH3-11.b       ............................................................
BnaA02.GH3-12.a      ............................................................
BnaA03.GH3-12.b      ............................................................
BnaA06.GH3-12.c      ............................................................
BnaA10.GH3-12.d      ............................................................
BnaC02.GH3-12.a      ............................................................
BnaC03.GH3-12.b      ............................................................
BnaC09.GH3-12.c      ............................................................
BnaA10.GH3-14.a      ............................................................
BnaC09.GH3-14.a      ............................................................
BnaA03.GH3-15.a      ............................................................
BnaC03.GH3-15.a      ............................................................
BnaAX.GH3-16.a       ............................................................
BnaC02.GH3-16.a      ............................................................
BnaA07.GH3-17.a      ............................................................
BnaA08.GH3-17.b      ............................................................
BnaA09.GH3-17.c      ............................................................
BnaC08.GH3-17.a      ............................................................
BnaCX.GH3-17.b       ............................................................
BnaCX.GH3-17.c       ............................................................
BnaA05GH3-18.a       ............................................................
BnaCX.GH3-18.a       ............................................................
BnaA06.GH3-19.a      ............................................................
BnaA09.GH3-19.b      ............................................................
consensus>70         ............................................................
```

```
AtGH3-1             ............................................................
AtGH3-2             ............................................................
AtGH3-3             ............................................................
AtGH3-4             ............................................................
AtGH3-5             ............................................................
AtGH3-6             ............................................................
AtGH3-7             ............................................................
AtGH3-8             ............................................................
AtGH3-9             ............................................................
AtGH3-10            ............................................................
AtGH3-11            ............................................................
AtGH3-12            ............................................................
AtGH3-13            ............................................................
AtGH3-14            ............................................................
AtGH3-15            ............................................................
AtGH3-16            ............................................................
AtGH3-17            ............................................................
AtGH3-18            ............................................................
AtGH3-19            ............................................................
BraX.GH3-1.a        ............................................................
BraX.GH3-2.a        ............................................................
BraA09.GH3-3.a      ............................................................
BraA01.GH3-5.a      ............................................................
BraA03.GH3-5.b      ............................................................
BraA02.GH3-6.a      ............................................................
BraA03.GH3-6.b      ............................................................
BraA06.GH3-7.a      ............................................................
BraA08.GH3-7.b      ............................................................
BraA02.GH3-8.a      ............................................................
BraA03.GH3-8.b      ............................................................
BraA03.GH3-8.c      ............................................................
BraA03.GH3-8.d      ............................................................
BraA06.GH3-8.e      ............................................................
BraA06.GH3-8.f      ............................................................
BraA09.GH3-8.g      ............................................................
BraA09.GH3-8.h      ............................................................
BraA10.GH3-8.i      ............................................................
BraA09.GH3-10.a     ............................................................
BraA03.GH3-11.a     ............................................................
BraA04.GH3-11.b     ............................................................
BraA05.GH3-11.c     ............................................................
BraA02.GH3-12.a     ............................................................
BraA03.GH3-12.b     ............................................................
BraA06.GH3-12.c     ............................................................
BraA10.GH3-12.d     ............................................................
BraA10.GH3-14.a     ............................................................
BraA03.GH3-15.a     ............................................................
BraA07.GH3-15.b     ............................................................
BraA02.GH3-16.a     ............................................................
BraA07.GH3-17.a     ............................................................
BraA09.GH3-17.b     ............................................................
BraAX.GH3-17.c      ............................................................
BraA04.GH3-19.a     ............................................................
BraA06.GH3-19.b     ............................................................
BraA09.GH3-19.c     ............................................................
BraA09.GH3-19.d     ............................................................
BraAX.GH3-19.e      ............................................................
BolC09.GH3-1.a      ............................................................
BolC01.GH3-2.a      ............................................................
BolC08.GH3-3.a      ............................................................
BolC01.GH3-5.a      ............................................................
BolC07.GH3-5.b      ............................................................
BolC02.GH3-6.a      ............................................................
BolC03.GH3-6.b      ............................................................
BolC06.GH3-7.a      ............................................................
BolC01.GH3-8.a      ..................................................QFLDMG....
BolC03.GH3-8.b      ............................................................
BolC07.GH3-8.c      ............................................................
BolC08.GH3-8.d      ............................................................
BolC09.GH3-8.e      ............................................................
BolC09.GH3-10.a     ............................................................
BolC03.GH3-11.a     ............................................................
BolC04.GH3-11.b     ............................................................
BolCX.GH3-11.c      ............................................................
BolC03.GH3-12.a     ............................................................
BolC09.GH3-12.b     ............................................................
BolC09.GH3-14.a     ............................................................
BolC03.GH3-15.a     ............................................................
BolCX.GH3-16.a      ............................................................
BolC07.GH3-17.a     ............................................................
BolC08.GH3-17.b     ............................................................
BolC08.GH3-19.a     ............................................................
BnaA09R.GH3-1.a     ............................................................
BnaCX.GH3-1.a       ............................................................
BnaA01.GH3-2.a      ............................................................
BnaC01.GH3-2.a      ............................................................
BnaA09.GH3-3.a      ............................................................
BnaC08.GH3-3.a      ............................................................
BnaA01.GH3-5.a      ............................................................
BnaA03.GH3-5.b      ............................................................
BnaC01.GH3-5.a      ............................................................
BnaC07.GH3-5.b      ............................................................
BnaA03.GH3-6.a      ............................................................
BnaAX.GH3-6.b       ............................................................
BnaC03.GH3-6.a      ............................................................
BnaCX.GH3-6.b       ............................................................
BnaA06.GH3-7.a      ............................................................
BnaA08.GH3-7.b      ............................................................
BnaC06.GH3-7.a      ............................................................
BnaA03.GH3-8.a      ............................................................
BnaA03.GH3-8.b      ............................................................
BnaA03.GH3-8.c      ............................................................
BnaA06.GH3-8.d      ............................................................
```

```
BnaA06.GH3-8.e      ............................................................
BnaA09.GH3-8.f      ............................................................
BnaA09.GH3-8.g      ............................................................
BnaAX.GH3-8.h       ............................................................
BnaAX.GH3-8.i       ............................................................
BnaC01.GH3-8.a      GLPQFPRPGGFPTNPMPFPQFPKPGFPQLPGQGFPKNPMPFPQFPKPGFPQFPGFGFPKF
BnaC02.GH3-8.b      ............................................................
BnaC02.GH3-8.c      ............................................................
BnaC02.GH3-8.d      ............................................................
BnaC07.GH3-8.e      ............................................................
BnaC09.GH3-8.f      ............................................................
BnaCX.GH3-8.g       ............................................................
BnaA05.GH3-9.a      ............................................................
BnaC04.GH3-9.a      ............................................................
BnaA03.GH3-10.a     ............................................................
BnaA09.GH3-10.b     ............................................................
BnaC09.GH3-10.a     ............................................................
BnaA03.GH3-11.a     ............................................................
BnaA04R.GH3-11.b    ............................................................
BnaA05.GH3-11.c     ............................................................
BnaC04.GH3-11.a     ............................................................
BnaCX.GH3-11.b      ............................................................
BnaA02.GH3-12.a     ............................................................
BnaA03.GH3-12.b     ............................................................
BnaA06.GH3-12.c     ............................................................
BnaA10.GH3-12.d     ............................................................
BnaC02.GH3-12.a     ............................................................
BnaC03.GH3-12.b     ............................................................
BnaC09.GH3-12.c     ............................................................
BnaA10.GH3-14.a     ............................................................
BnaC09.GH3-14.a     ............................................................
BnaA03.GH3-15.a     ............................................................
BnaC03.GH3-15.a     ............................................................
BnaAX.GH3-16.a      ............................................................
BnaC02.GH3-16.a     ............................................................
BnaA07.GH3-17.a     ............................................................
BnaA08.GH3-17.b     ............................................................
BnaA09.GH3-17.c     ............................................................
BnaC08.GH3-17.a     ............................................................
BnaCX.GH3-17.b      ............................................................
BnaCX.GH3-17.c      ............................................................
BnaA05GH3-18.a      ............................................................
BnaCX.GH3-18.a      ............................................................
BnaA06.GH3-19.a     ............................................................
BnaA09.GH3-19.b     ............................................................
consensus>70        ............................................................
```

```
AtGH3-1            ................................
AtGH3-2            ................................
AtGH3-3            ................................
AtGH3-4            ................................
AtGH3-5            ................................
AtGH3-6            ................................
AtGH3-7            ................................
AtGH3-8            ................................
AtGH3-9            ................................
AtGH3-10           ................................
AtGH3-11           ................................
AtGH3-12           ................................
AtGH3-13           ................................
AtGH3-14           ................................
AtGH3-15           ................................
AtGH3-16           ................................
AtGH3-17           ................................
AtGH3-18           ................................
AtGH3-19           ................................
BraX.GH3-1.a       ................................
BraX.GH3-2.a       ................................
BraA09.GH3-3.a     ................................
BraA01.GH3-5.a     ................................
BraA03.GH3-5.b     ................................
BraA02.GH3-6.a     ................................
BraA03.GH3-6.b     ................................
BraA06.GH3-7.a     ................................
BraA08.GH3-7.b     ................................
BraA02.GH3-8.a     ................................
BraA03.GH3-8.b     ................................
BraA03.GH3-8.c     ................................
BraA03.GH3-8.d     ................................
BraA06.GH3-8.e     ................................
BraA06.GH3-8.f     ................................
BraA09.GH3-8.g     ................................
BraA09.GH3-8.h     ................................
BraA10.GH3-8.i     ................................
BraA09.GH3-10.a    ................................
BraA03.GH3-11.a    ................................
BraA04.GH3-11.b    ................................
BraA05.GH3-11.c    ................................
BraA02.GH3-12.a    ................................
BraA03.GH3-12.b    ................................
BraA06.GH3-12.c    ................................
BraA10.GH3-12.d    ................................
BraA10.GH3-14.a    ................................
BraA03.GH3-15.a    ................................
BraA07.GH3-15.b    ................................
BraA02.GH3-16.a    ................................
BraA07.GH3-17.a    ................................
BraA09.GH3-17.b    ................................
BraAX.GH3-17.c     ................................
BraA04.GH3-19.a    ................................
BraA06.GH3-19.b    ................................
BraA09.GH3-19.c    ................................
BraA09.GH3-19.d    ................................
BraAX.GH3-19.e     ................................
BolC09.GH3-1.a     ................................
BolC01.GH3-2.a     ................................
BolC08.GH3-3.a     ................................
BolC01.GH3-5.a     ................................
BolC07.GH3-5.b     ................................
BolC02.GH3-6.a     ................................
BolC03.GH3-6.b     ................................
BolC06.GH3-7.a     ................................
BolC01.GH3-8.a     ................................
BolC03.GH3-8.b     ................................
BolC07.GH3-8.c     ................................
BolC08.GH3-8.d     ................................
BolC09.GH3-8.e     ................................
BolC09.GH3-10.a    ................................
BolC03.GH3-11.a    ................................
BolC04.GH3-11.b    ................................
BolCX.GH3-11.c     ................................
BolC03.GH3-12.a    ................................
BolC09.GH3-12.b    ................................
BolC09.GH3-14.a    ................................
BolC03.GH3-15.a    ................................
BolCX.GH3-16.a     ................................
BolC07.GH3-17.a    ................................
BolC08.GH3-17.b    ................................
BolC08.GH3-19.a    ................................
BnaA09R.GH3-1.a    ................................
BnaCX.GH3-1.a      ................................
BnaA01.GH3-2.a     ................................
BnaC01.GH3-2.a     ................................
BnaA09.GH3-3.a     ................................
BnaC08.GH3-3.a     ................................
BnaA01.GH3-5.a     ................................
BnaA03.GH3-5.b     ................................
BnaC01.GH3-5.a     ................................
BnaC07.GH3-5.b     ................................
BnaA03.GH3-6.a     ................................
BnaAX.GH3-6.b      ................................
BnaC03.GH3-6.a     ................................
BnaCX.GH3-6.b      ................................
BnaA06.GH3-7.a     ................................
BnaA08.GH3-7.b     ................................
BnaC06.GH3-7.a     ................................
BnaA03.GH3-8.a     ................................
BnaA03.GH3-8.b     ................................
BnaA03.GH3-8.c     ................................
BnaA06.GH3-8.d     ................................
```

**BnaA06.GH3-8.e**	....
**BnaA09.GH3-8.f**	....
**BnaA09.GH3-8.g**	....
**BnaAX.GH3-8.h**	....
**BnaAX.GH3-8.i**	....
**BnaC01.GH3-8.a**	PQFPKPGSPSFPPATPTISTPPSIPVTPTLSN
**BnaC02.GH3-8.b**	....
**BnaC02.GH3-8.c**	....
**BnaC02.GH3-8.d**	....
**BnaC07.GH3-8.e**	....
**BnaC09.GH3-8.f**	....
**BnaCX.GH3-8.g**	....
**BnaA05.GH3-9.a**	....
**BnaC04.GH3-9.a**	....
**BnaA03.GH3-10.a**	....
**BnaA09.GH3-10.b**	....
**BnaC09.GH3-10.a**	....
**BnaA03.GH3-11.a**	....
**BnaA04R.GH3-11.b**	....
**BnaA05.GH3-11.c**	....
**BnaC04.GH3-11.a**	....
**BnaCX.GH3-11.b**	....
**BnaA02.GH3-12.a**	....
**BnaA03.GH3-12.b**	....
**BnaA06.GH3-12.c**	....
**BnaA10.GH3-12.d**	....
**BnaC02.GH3-12.a**	....
**BnaC03.GH3-12.b**	....
**BnaC09.GH3-12.c**	....
**BnaA10.GH3-14.a**	....
**BnaC09.GH3-14.a**	....
**BnaA03.GH3-15.a**	....
**BnaC03.GH3-15.a**	....
**BnaAX.GH3-16.a**	....
**BnaC02.GH3-16.a**	....
**BnaA07.GH3-17.a**	....
**BnaA08.GH3-17.b**	....
**BnaA09.GH3-17.c**	....
**BnaC08.GH3-17.a**	....
**BnaCX.GH3-17.b**	....
**BnaCX.GH3-17.c**	....
**BnaA05GH3-18.a**	....
**BnaCX.GH3-18.a**	....
**BnaA06.GH3-19.a**	....
**BnaA09.GH3-19.b**	....
consensus>70	....
