# Supplementary material for: The Gene Structure and Expression Level Changes of the GH3 Gene Family in Brassica napus Relative to Its Diploid Ancestors
Source: Genes (Basel). 2019 Jan 17;10(1):58. doi: 10.3390/genes10010058 (PMC6356818; doi:10.3390/genes10010058)
Supplement: Supplementary file 1 [file genes-10-00058-s001.zip › Supplementary files/Supplementary Figures/Figure S2.docx]

Figure S2 The 3-dimensional structures of 15 orthologous protein pairs.

| BraA01.GH3-5.a | BnaA01.GH3-5.a |
| --- | --- |
| 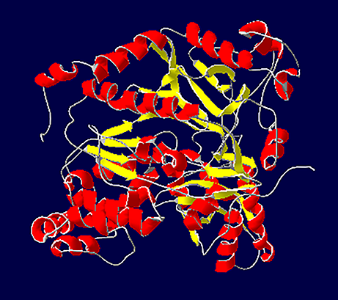 | 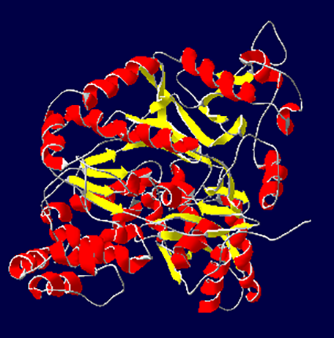 |
| BraA03.GH3-5.b | BnaA03.GH3-5.b |
| 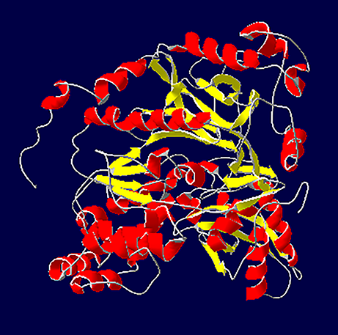 | 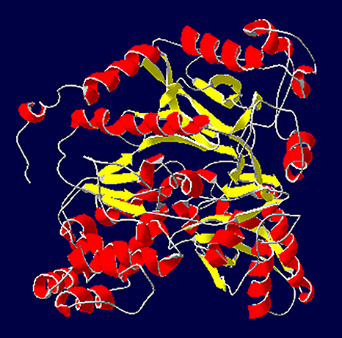 |
| BolC01.GH3-5.a | BnaC01.GH3-5.a |
| 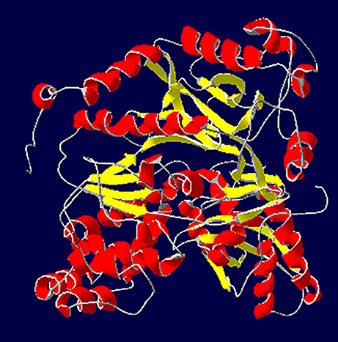 | 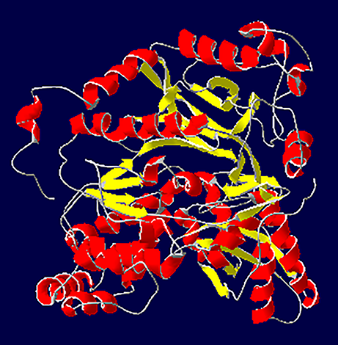 |
| BolC07.GH3-5.b | BnaC07.GH3-5.b |
| 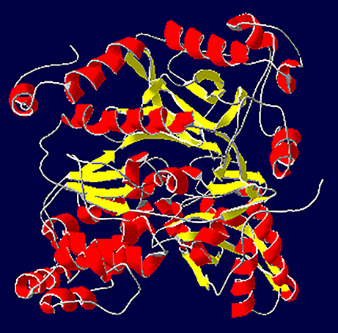 | 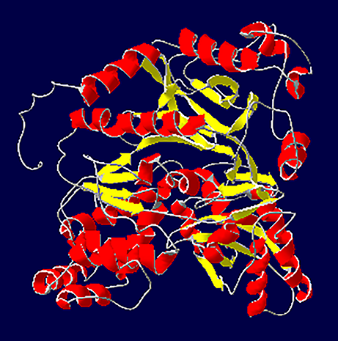 |
| BraA03.GH3-11.a | BnaA03.GH3-11.a |
| 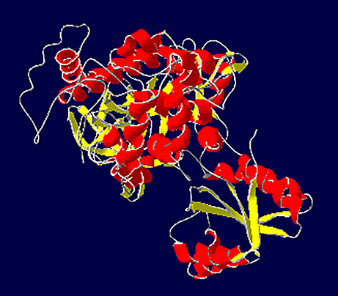 | 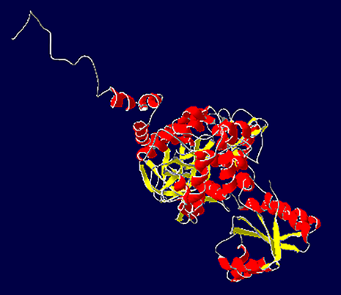 |
| BraA04.GH3-11.b | BnaA04R.GH3-11.b |
| 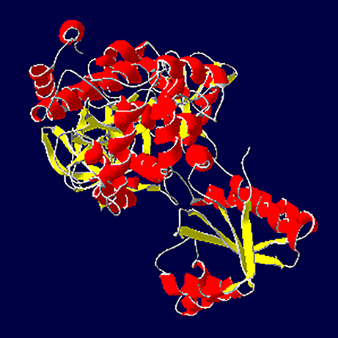 | 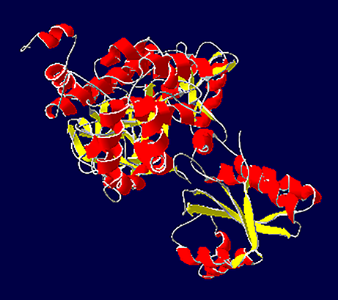 |
| BraA05.GH3-11.c | BnaA05.GH3-11.c |
| 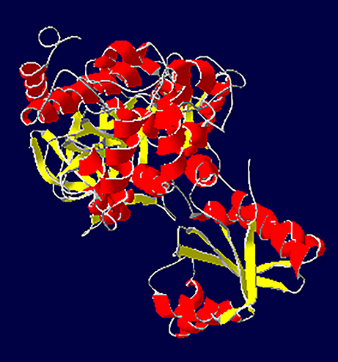 | 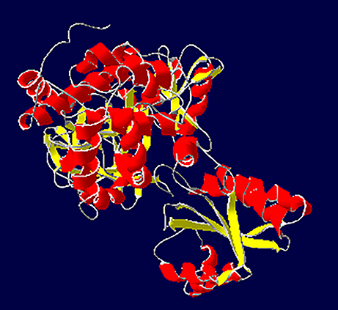 |
| BolC03.GH3-11.a | BnaCX.GH3-11.b |
| 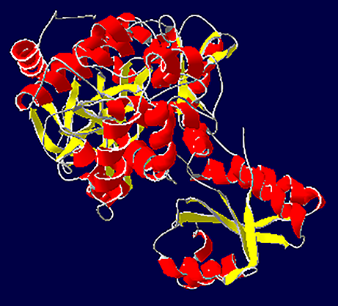 | 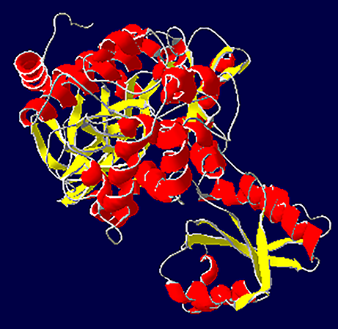 |
| BraA02.GH3-12.a | BnaA02.GH3-12.a |
| 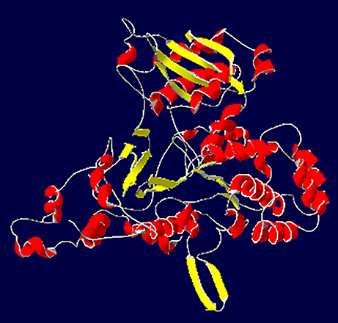 | 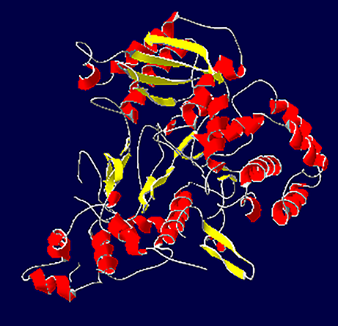 |
| BraA03.GH3-12.b | BnaA03.GH3-12.b |
| 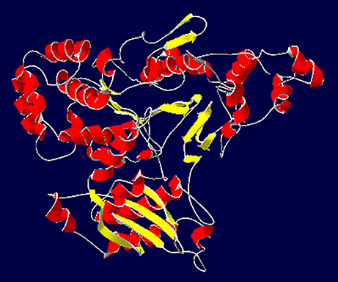 | 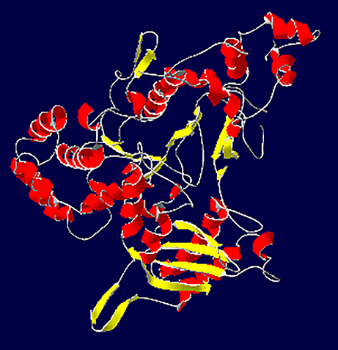 |
| BraA06.GH3-12.c | BnaA06.GH3-12.c |
| 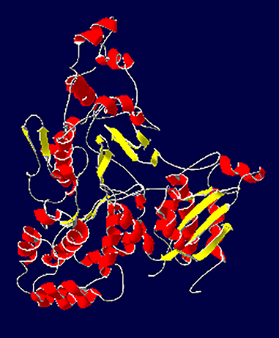 | 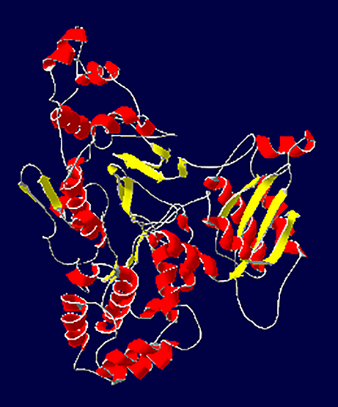 |
| BraA10.GH3-12.d | BnaA10.GH3-12.d |
| 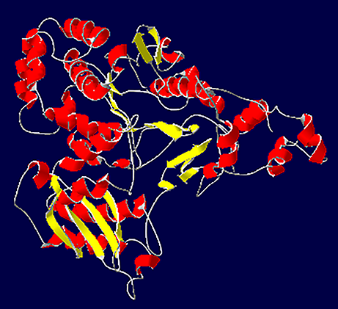 | 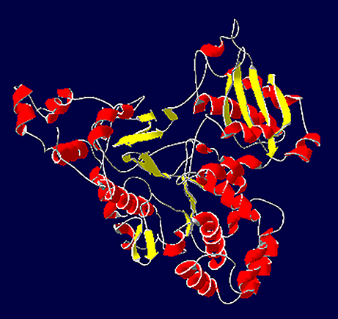 |
| BolC03.GH3-12.a | BnaC03.GH3-12.b |
| 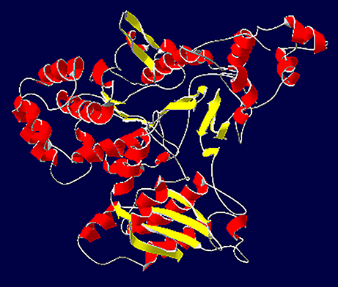 | 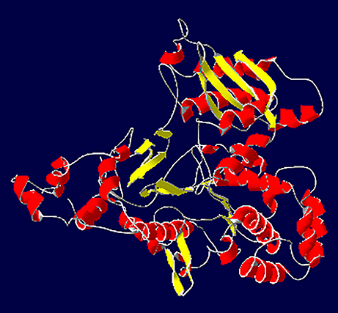 |
| BolC09.GH3-12.b | BnaC09.GH3-12.c |
| 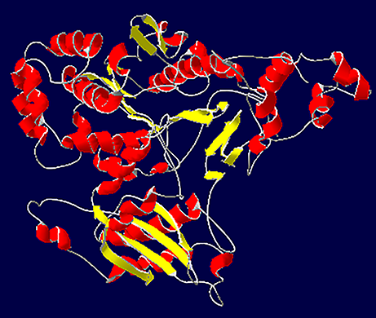 | 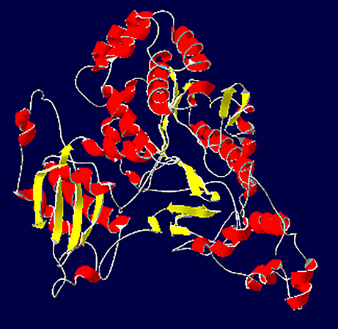 |
| BraA03.GH3-15.a | BnaA03.GH3-15.a |
| 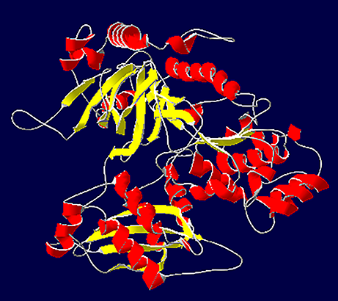 | 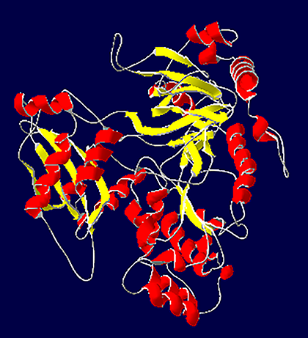 |
