# Supplementary material for: The Gene Structure and Expression Level Changes of the GH3 Gene Family in Brassica napus Relative to Its Diploid Ancestors
Source: Genes (Basel). 2019 Jan 17;10(1):58. doi: 10.3390/genes10010058 (PMC6356818; doi:10.3390/genes10010058)

Figure S3 The time of WGT and allopolyploidization events and the number of *GH3* genes that were missing or new in the different time. The genes colored by green were missing *GH3* genes, and the genes colored by red were newly produced *GH3* genes.


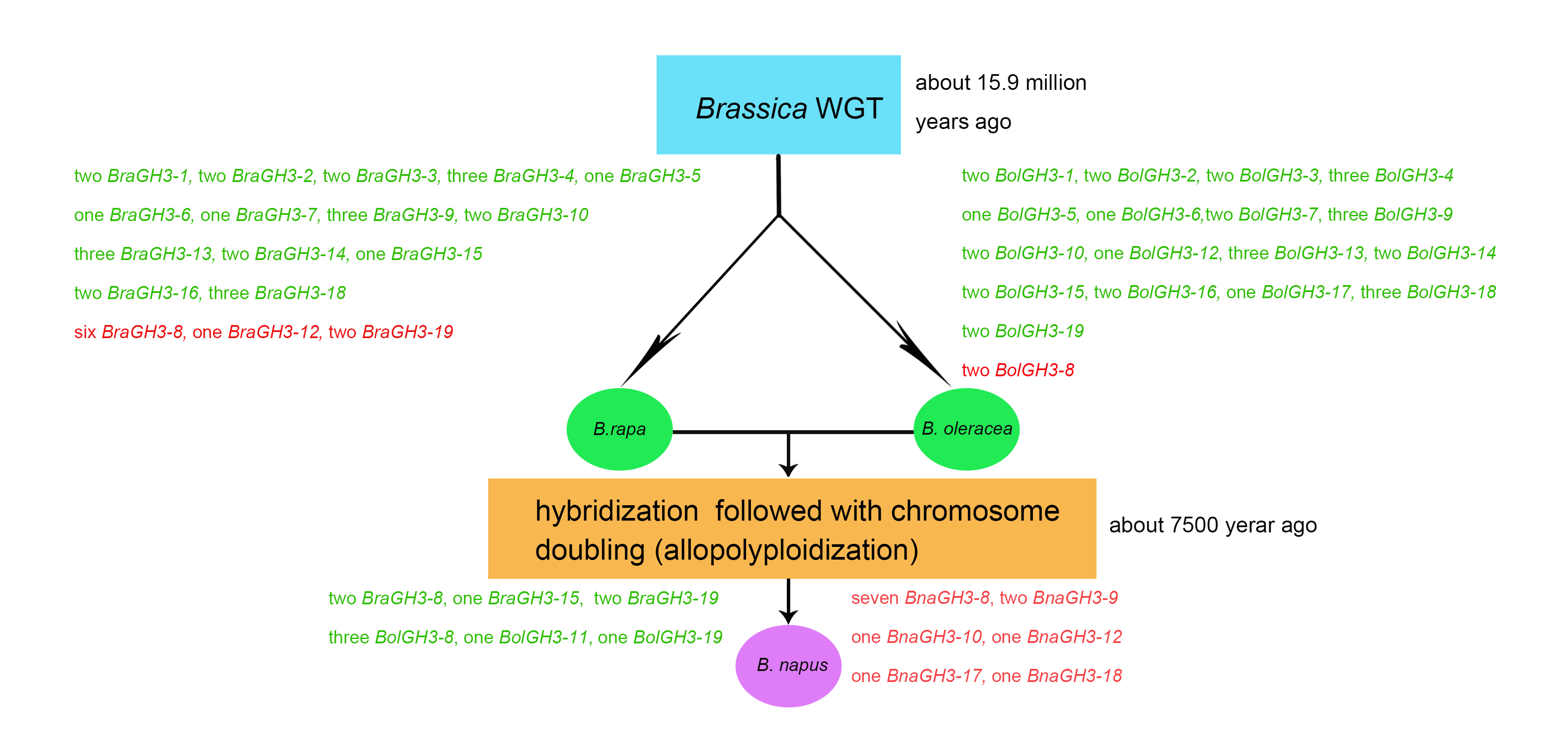

Supplement: Supplementary file 1 [file genes-10-00058-s001.zip › Supplementary files/Supplementary Figures/Figure S3.docx]
